# Orthogonal Optimization Research on Various Nozzles of High-Speed Centrifugal Spinning

**DOI:** 10.3389/fbioe.2022.884316

**Published:** 2022-05-17

**Authors:** Zhiming Zhang, Kang Liu, Wenhui Li, Qiaoling Ji, Qiao Xu, Zilong Lai, Changjin Ke

**Affiliations:** ^1^ Hubei Digital Textile Equipment Key Laboratory, Wuhan Textile University, Wuhan, China; ^2^ School of Mechanical Engineering and Automation, Wuhan Textile University, Wuhan, China; ^3^ Hubei Province Fiber Inspection Bureau, Wuhan, China

**Keywords:** centrifugal spinning, stepped nozzle, conical-straight nozzle, conical nozzle, curved-tube nozzle, orthogonal optimization

## Abstract

High-speed centrifugal spinning is a burgeoning method of fabricating nanofibers by use of the centrifugal force field. This article studied four different spinning nozzles, which were called stepped nozzle, conical-straight nozzle, conical nozzle, and curved-tube nozzle, to explore the optimal nozzle structures for fabricating nanofibers. According to the principle of centrifugal spinning, the spinning solution flow states within the four nozzles were analyzed, and the solution outlet velocity model was established. Then, the structural parameters of the four kinds of nozzles were optimized with the spinning solution outlet velocity as the test index by combining the orthogonal test and numerical simulation. Based on the orthogonal test results, the influence of nozzle structure parameters on the solution outlet velocity was analyzed, and the best combination of parameters of the centrifugal spinning nozzle structure was obtained. Subsequently, the four kinds of nozzles were used to fabricate nanofibers in the laboratory, under different solution concentration, motor rotation speed, and outlet diameters. Finally, the scanning electron microscope (SEM) was applied to observe the morphology and surface quality of nanofibers. It was found that the surface of nanofibers manufactured by the conical-straight nozzle and curved-tube nozzle was smoother than that by stepped and conical nozzles, and the fiber diameter by the conical-straight nozzle was minimal, followed by curved-tube nozzles, stepped nozzles, and conical nozzles in the diameter distribution of nanofibers.

## Introduction

Nanofiber referring to a fiber below 1000 nm diameter is regarded as a one-dimensional linear material (Kenry and Lim, 2017) with great application potential for its large specific surface area, high length–diameter ratio, and high porosity (Bergshoef and Vancso, 2010; Wu et al., 2016) and also has great commercial and industrial application value in sensors (Zhu and Wang, 2017; Unal et al., 2018; Yang et al., 2019), airborne filtration (Riyadh et al., 2018; Huang et al., 2019), drug delivery (Wang et al., 2016; Hu et al., 2014; Sang et al., 2017), and energy storage system (Ponnamma et al., 2020), due to its unique conductive properties, high porosity, low density, and biocompatibility. Moreover, inorganic nanofibers can be utilized to make gas sensors that detect the concentration of nitrogen dioxide, ozone, and sulfur dioxide in the air (Gong and Wan, 2015). In addition, it can be also used in biomedical aspects, such as human tissue stents made by polymer (Heidari et al., 2019; Li et al., 2020), conducive to the attachment and reproduction of human cells. For the increasing demand of high-quality nanofibers, various techniques have been applied to fabricate multifarious polymer nanofibers, such as self-assembly (Malfatti and Innocenzi, 2011), melt-blown (Ellison et al., 2007), phase separation (Wang et al., 2010), and electrospinning (Cheng et al., 2010; Wang F. et al., 2021).

However, the aforementioned conventional methods for spinning nanofibers have the inherent drawback. For instance, the extra high electric field is utilized between the nozzle and collecting plate during electrospinning, which results in the danger during operation, and for melt spinning, although this technology has been very mature and the process is highly operable, its further application is limited due to the fibers with poor mechanical properties and heat resistance. High-speed centrifugal spinning is a novel method to fabricate fiber through inertial force which is generated by rotating nozzles (Sun et al., 2018). This method has many distinctive advantages compared with the conventional ways to prepare nanofibers. First, it has high production efficiency. Second, the spinning materials and solvent materials have a wide range of choices to fabricate different polymer nanofibers. Third, spinning equipment is relatively simple, and the energy consumption is also low. Thus, it has huge commercial application potential. A growing number of researchers are using centrifugal spinning to prepare nanofibers.

Mehmet and Ali, (2020) compared the preparation of nanofiber silica (SiO_2_) felt by simple and innovative nonelectrospinning routes, solution blowing, and centrifugal spinning (CS) and found that the felt produced by CS was conducive to thermal insulation application. Meanwhile, the TiO_2_ and the self-polarized polyvinylidene fluorides (PVDF) nanofibers were manufactured by centrifugal spinning (Aminirastabi et al., 2020; Ibtehaj et al., 2020). The sucrose-based microfibers prepared from centrifuge spinning have the properties that enhance drug dissolution and oral absorption (Stefania et al., 2016). Composite nanofibers prepared from multicomponent materials also show excellent performance. The carboxymethyl chitosanpolyethylene oxide (CMCS/PEO) composite nonwoven mats by centrifugal spinning were available for wound dressings (Li et al., 2020; Wang Y. et al., 2021). Chen et al. (2019) researched the polyacrylonitrile/polyethylene glycol phase-change material fibers prepared *via* centrifugal spinning and found that these nanofibers had good thermophysical properties. Subsequently, more scholars focused on the electrical effect of the nanofibers. Orrostieta et al. (2021) fabricated the composite nanofibers as the anode material of lithium ion and sodium ion batteries and analyzed their morphology and structure. Also, some researchers had combined centrifugal spinning with other techniques to prepare fibers with a smaller diameter distribution (Fang et al., 2016). The multidimensional public opinion process was modeled on the basis of a complex network dynamics model in the context of derived topics (Chen et al., 2021).

There are many studies working on revealing the relationship between the process parameters and the morphology and the diameter distribution of the prepared nanofibers. The diameter model of the nanofibers was established, and the key parameters of the centrifugal spinning system were studied (Li et al., 2019). The effect of five important dimensionless groups on the steady-state trajectory and reduced fiber radius was analyzed when the spinning solution was subjected to multiple forces (Noroozi et al., 2017). Natarajan and Bhargava (2018) discussed the effect of rotational speed of the spinneret and viscosity of solution on the spinning fiber quality. Nozzle is an important part that has a direct influence on fiber morphology and diameter. Subsequent researches had studied the influence of nozzle length, outlet diameter, and outlet direction on fiber morphology and diameter distribution (Lu et al., 2013; Zhmayev et al., 2015; Zhang et al., 2020). Therefore, the optimal process parameters for fabricating nanofibers could be obtained by optimizing these parameters influencing nanofibers (Elena et al., 2018).

There are many factors affecting the quality of nanofibers fabricated by centrifugal spinning, including solution parameters such as material types and concentration, nozzle structure parameters, and process parameters such as rotation velocity. Therefore, the most suitable nozzle parameters need to be explored in order to improve the efficiency and quality preparation of nanofibers by centrifugal spinning. For this reason, Lai et al. (2021) proposed four different nozzle structures to explore the optimal structures for centrifugal spinning, but the other three nozzles (stepped nozzle, conical-straight nozzle, and conical nozzle) were not analyzed systematically and optimized and nor compared with the optimized curved-tube nozzles.

In this article, these four nozzle structures are studied to obtain the optimal nozzle structure, which can fabricate nanofibers with better surface quality and are more uniform in diameter. All the four nozzles were optimized by orthogonal test and simulation, and then, the nozzles with the best parameter structures were used for centrifugal spinning experiments. Finally, the morphology and surface quality of nanofibers were observed by SEM. The results showed that the conical-straight nozzle and curved-tube nozzle could fabricate nanofibers with better surface quality and smaller diameter, and the conical-straight nozzle could manufacture nanofibers with minimal diameter.

## The Principle of Centrifugal Spinning

**FIGURE 1:**
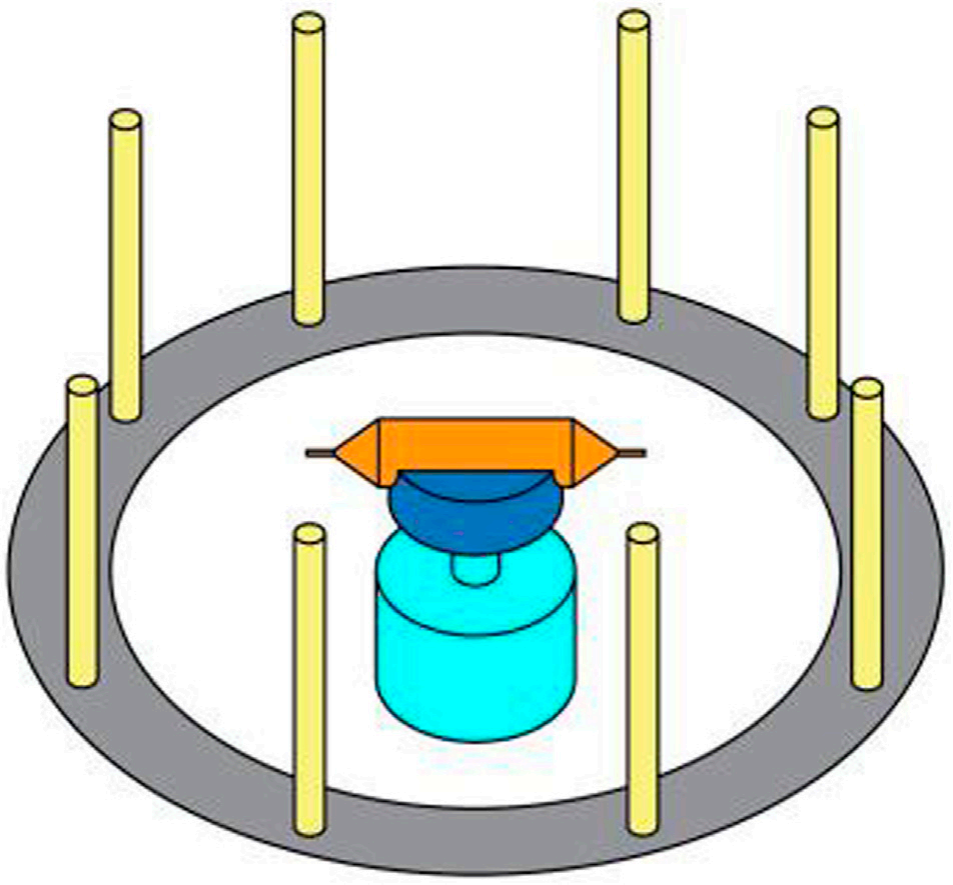
Device of centrifugal spinning.

**FIGURE 2:**
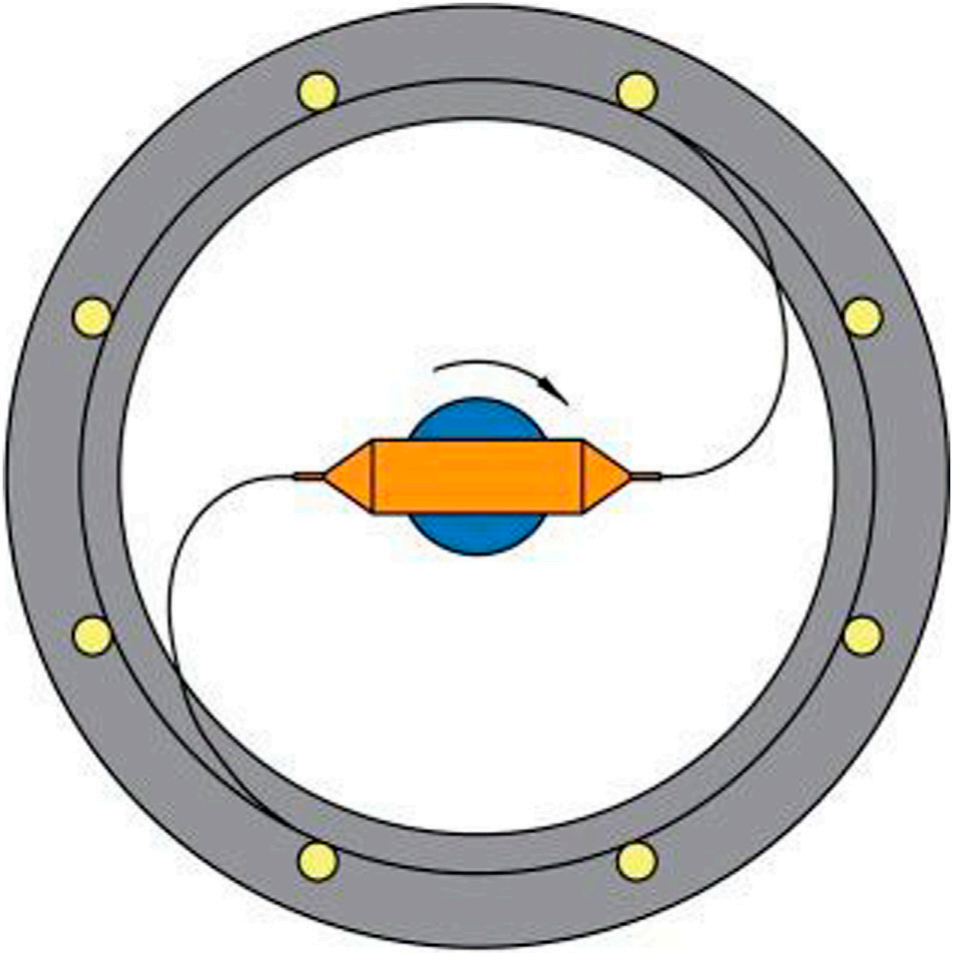
Process of centrifugal spinning.

The drive motor shaft is connected to the fastener, and the solution container is connected with spinning nozzles by screw threads and secured to the fastener. The collectors are stainless steel rods. The device of centrifugal is shown in Figure 1. The high-speed centrifugal spinning process is shown in Figure 2. When the device works, the nozzle rotates at a high speed with the drive motor rotating. The spinning solution flows to the nozzle under centrifugal force and is ejected from the nozzle outlet to form a spinning jet. Then, as the solvent evaporates, the jet moves along the curve in the air and stretches into nanofibers under the inertial force. Finally, the nanofibers are collected by the collectors.

### The Solution Flow in the Container

**FIGURE 3:**
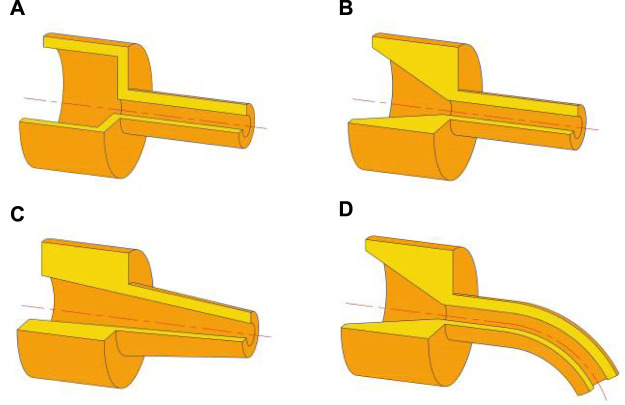
Four different kinds of spinning nozzles. **(A)** Stepped nozzle; **(B)** conical-straight nozzle; **(C)** conical nozzle; and **(D)** curved-tube nozzle.

At present, the research studies on high-speed centrifugal spinning are mainly focused on the property of fibers spun, the spinning solution parameters, and solution jet motion trajectory, but the optimization of the centrifugal spinning device, especially on the structural design of the nozzle, still requires further exploration. According to the principle of high-speed centrifugal spinning, this article studied four different nozzle structures called ladder nozzle, conical-straight nozzle, conical nozzle, and curved-tube nozzle, as shown in Figure 3.

**FIGURE 4:**
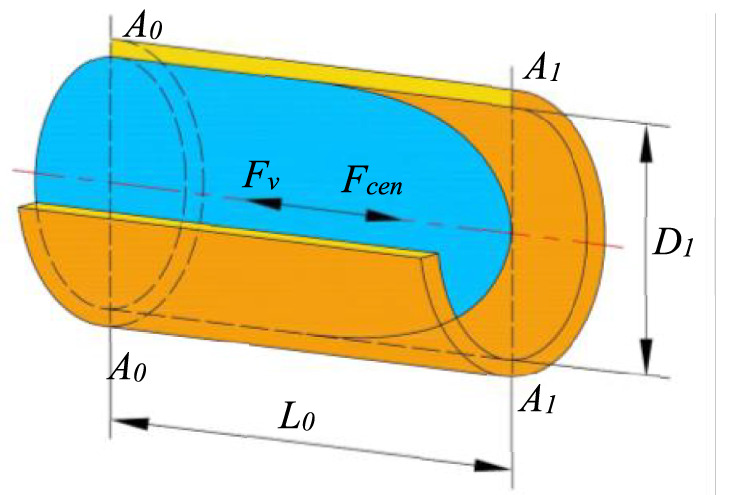
Movement of spinning solution in the container.

During the process of high-speed centrifugal spinning, the movement of the solution in the container is shown in Figure 4. *A*_*0*_ is the cross section of the rotation shaft of the solution container, and *A*_*1*_ is the inlet cross section of the nozzle. The distance between the cross-section *A*_*0*_ to the inlet cross-section *A*_*1*_ is *L*_*0*_. The diameter of the solution container pipe is *D*_*1*_.

The movement of the spinning solution flowing to the stepped nozzle inlet is mainly influenced by the viscous force *F*_*v*_ and the centrifugal force *F*_*cen*_, and the equation of motion of the spinning solution is as follows:

$$\frac{d}{dt}\left(m\vec{V_{1}}\right)=\vec{F_{v}}+\vec{F_{cen}},$$ (1)

where *V*_*1*_ is the average velocity of the spinning solution at the cross-section *A*_*1*_.

The formulas of the viscosity force *F*_*1*_ and the centrifugal force *F*_*2*_ are expressed as:

$$\{\begin{array}{c} F_{v}=k{\left(\frac{du}{dr}\right)}^{n};\\ F_{cen}=mL_{0}\omega^{2}, \end{array}$$ (2)

where *k* is the viscosity coefficient of the spinning solution, *du/dr* is the velocity gradient of the spinning solution, *n* is the rheological index of the spinning solution, indicating the rheological characteristics of the spinning solution, *m* is the mass of the spinning solution, and *ω* is the rotation angular velocity of the solution container.

Substituting Eq. 2 into Eq. 1, the average velocity of the spinning solution at the nozzle inlet cross-section *A*_*1*_ can be written as:

$$V_{1}=\frac{mL_{0}\omega^{2}-k{\left(\frac{du}{dr}\right)}^{n}}{m}t_{0},$$ (3)

where *t*_*0*_ is the rotation time of the solution container.

### The Stepped Nozzle

The stepped nozzle flow channel structure is composed of two cylindrical pipes of different diameters, including the inlet and outlet pipes, as shown in Supplementary Figure S1A. The total length of the stepped nozzle is *L*_*2*_; the length of the inlet section pipe is *L*_*1*_; the inlet diameter is *D*_*1*_; and the diameter of the nozzle outlet is *D*_*2*_ *.* Supplementary Figure S1B shows the motion of the spinning solution in the stepped nozzle. Cross-section *A*_*1*_ to cross-section *A*_*2*_ is the stepped nozzle inlet area, and cross-section *A*_*2*_ to cross-section *A*_*3*_ is the stepped nozzle outlet area. The average velocity of the solution at cross-sections *A*_*1*_ *, A*_*2*_, and *A*_*3*_ is *V*_*1*_, *V*_*2*_, and *V*_*3*_, respectively, during the fabrication of the fiber.

The motion of the spinning solution in the nozzle can be seen as a constant flow, and the outlet velocity can be obtained according to the continuity equation of the spinning solution in the nozzle (Lai et al., 2021):

$$V_{3}=\frac{\left[m\left(L_{0}+L_{1}\right)\omega^{2}-k{\left(\frac{du}{dr}\right)}^{n}\right]D_{1}^{2}}{mD_{2}^{2}}t_{0}.$$ (4)

The outlet velocity of the spinning solution in the stepped nozzle is related to the length of the inlet section, the inlet diameter, and the outlet diameter of the stepped nozzle.

### The Conical-Straight Nozzle

The conical-straight nozzle structure consists of conical inlet and cylindrical outlet pipes, as shown in Supplementary Figure S2A. The total length of the conical-straight nozzle is *L*_*2*_; the length of the conical inlet area is *L*_*1*_; and the inlet area shrinkage angle is *α*. The nozzle inlet diameter is *D*_*1*_, and the outlet diameter is *D*_*2*_. The motion of the spinning solution in the conical-straight nozzle is shown in Supplementary Figure S2B. Cross-section *A*_*1*_ to cross-section *A*_*2*_ is the conical nozzle inlet area, and cross-section *A*_*2*_ to cross-section *A*_*3*_ is the conical nozzle outlet area.

Therefore, the average velocity of cross-section *A*_*3*_ of the conical-straight nozzle is obtained as follows (Lai et al., 2021):

$$V_{3}=V_{2}=\frac{\left[mL_{0}\omega^{2}-k{\left(\frac{du}{dr}\right)}^{n}\right]D_{1}^{2}}{m{\left(\frac{D_{1}}{2}-L_{1}\tan\frac{\alpha }{2}\right)}^{2}}t_{0}.$$ (5)

The outlet velocity is related to the inlet length, inlet diameter, and the shrinkage angle of the conical-straight nozzle. The shrinkage angle of the conical-straight nozzle is determined by the nozzle inlet diameter, outlet diameter, and the length of the inlet area.

### The Conical Nozzle

The conical nozzle structure is a conical pipe, as shown in Supplementary Figure S3A. The total length of the conical nozzle is *L*_*2*_, the nozzle inlet diameter is *D*_*1*_, the outlet diameter is *D*_*2*_, and the conical pipe shrinkage angle is α. The motion of the spinning solution in the conical nozzle is shown in Supplementary Figure S3B. Cross-section *A*_*1*_ is the conical nozzle inlet section, and cross-section face *A*_*2*_ is the conical nozzle outlet section. During high-speed centrifugal spinning, the inlet and outlet velocity of the spinning solution is *V*_*1*_ and *V*_*2*_ in the conical nozzle, respectively.

According to Eq. 8, the average velocity of the spinning solution at the conical nozzle outlet *A*_*2*_ surface is (Lai et al., 2021):

$$V_{2}=\frac{\left[mL_{0}\omega^{2}-k{\left(\frac{du}{dr}\right)}^{n}\right]D_{1}^{2}}{m{\left(\frac{D_{1}}{2}-L_{2}\tan\frac{\alpha }{2}\right)}^{2}}t_{0}.$$ (6)

The outlet velocity of the spinning solution in the conical nozzle is related to the total length, the inlet diameter, and the shrinkage angle of the conical pipe.

### The Curved-Tube Nozzle

The curved-tube nozzle structure is shown in Supplementary Figure S4A. The total length of the curved-tube nozzle is *L*_*2*_; the inlet diameter is *D*_*1*_; and the outlet diameter is *D*_*2*_. The shrinkage angle of the conical inlet pipe is α, and the length of this area is *L*_*1*_. The curvature radius of the nozzle outlet area is *R*, and the bent center corner is θ. Supplementary Figure S4B shows the motion of the spinning solution in the curved-tube nozzle. Cross-section *A*_*3*_ to cross-section *A*_*4*_ is the curved nozzle section. *V*_*3*_ is the average velocity of the spinning solution at cross-section *A*_*3*_ in the curved-tube nozzle. *V*_*4*_ is the average velocity of the spinning solution at the cross-section face *A*_*4*_ of the curved nozzle outlet.

Substituting Eq. 11 into Eq. 12, the average velocity of the spinning solution at the curved-tube nozzle outlet cross-section *A*_*4*_ is (Lai et al., 2021):

$$V_{4}=\frac{\left[mL_{0}\omega^{2}-k{\left(\frac{du}{dr}\right)}^{n}\right]D_{1}^{2}\cos\theta }{m{\left(\frac{D_{1}}{2}-L_{1}\tan\frac{\alpha }{2}\right)}^{2}}t_{0}.$$ (7)

The outlet velocity of the spinning solution in the curved-tube nozzle is related to the inlet section length, inlet diameter, radius of the bending curvature, and shrinkage angle of the inlet area. The shrinkage angle of the curved-tube nozzle is determined by the nozzle inlet diameter, outlet diameter, and the length of the inlet area.

## Different High-Speed Centrifugal Spinning Nozzle Orthogonal Simulation Optimization

According to the aforementioned formula of the outlet velocity of the spinning solution in the centrifugal spinning, the relationship between the centrifugal spinning nozzle structural parameter and the solution outlet velocity was found. Therefore, in order to maximize the outlet velocity of the spinning solution, the nozzle structural parameters were optimized. The combination of numerical simulation and orthogonal tests was used for the nozzle structural parameter optimization. Various test schemes were obtained in this chapter through combining the structural parameters of the stepped nozzle, conical-straight nozzle, conical nozzle, and curved-tube nozzle by the orthogonal test design. Through numerical simulation of the flow field in each test scheme, the velocity distribution and turbulent kinetic energy of the spinning solution were analyzed, and the optimal nozzle structural parameters were obtained.

### The Orthogonal and Simulation Optimization of the Stepped Nozzle

During high-speed centrifugal spinning, the spinning solution flows from the container to the nozzle outlet and is finally ejected. Thus, the spinning solution flow field model is composed of the solution container flow channel and the nozzle flow channel. The solution container and the spinning nozzle at both ends together form the centrifugal spinning spinneret, and the 3D structure of the stepped nozzle spinning spinneret is shown in Supplementary Figure S5A. The length of the solution container is 60 mm, and the diameter of the container pipe is the same as the nozzle inlet. The unstructured grids are used in dividing the channel model of the stepped nozzle, as shown in Supplementary Figure S5B. The maximum mesh size of the overall mesh is set to 0.1 mm to ensure the mesh division quality at the nozzle and the accuracy of the simulation results. The boundary layer grid structure of the stepped nozzle is a hexahedral grid, and the boundary layer is divided into three layers.

In order to obtain the optimal combination of the structural parameters of the stepped nozzle, orthogonal tests are required, and the exit velocity of the spinning solution in the stepped nozzle is used as an important indicator. The total length, the length of inlet section, inlet diameter, and outlet diameter of the stepped nozzle are selected as four factors of the orthogonal test, indicated by A, B, C, and D, respectively. Orthogonal numerical simulation tests were designed for four factors, each with three levels, and the orthogonal factor level table is shown in Table 1.

**TABLE 1:** Factors and levels of the orthogonal test of the stepped nozzle.

Level	Factor (mm)			
	A	B	C	D
	Total length	Length of the inlet section	Inlet diameter	Outlet diameter
1	15	6	10	**0.6**
2	**20**	**8**	12	0.8
3	25	10	**14**	1.0

Note: The bold values represents the best combinations of parameters.

Nine test schemes of different structural parameters were obtained, and numerical simulation of the flow field of the stepped nozzle spinneret in nine groups was carried out. The outlet velocity of the spinning solution at the stepped nozzle at 4000 rpm speed is shown in Table 2.

**TABLE 2:** Results of the orthogonal test of the stepped nozzle.

Number	A	B	C	D	Outlet velocity (m/s)
1	15	6	10	0.6	91.2005
2	15	8	12	0.8	80.8389
3	15	10	14	1.0	74.6527
4	20	6	12	1.0	54.3471
5	**20**	**8**	**14**	**0.6**	**201.2442**
6	20	10	10	0.8	54.4257
7	25	6	14	0.8	115.3712
8	25	8	10	1.0	39.6422
9	25	10	12	0.6	140.7523

Note: The bold values represents the best combinations of parameters.

The flow velocity cloud distribution of nine test schemes is shown in Supplementary Figure S6. The flow velocity increases as the spinning solution flows from the right inlet to the left outlet. Supplementary Figure S7 shows the cloud map of the flow velocity distribution of cross-section of the stepped nozzle outlet in nine test schemes. The high-speed area of the spinning solution flow velocity is concentrated on the left side of the nozzle outlet pipe wall in the nine test schemes, and the solution flow speed gradually decreases from the left wall of the nozzle outlet to the right.

Differential analysis is used to study the influence of the parameter combination on the outlet velocity, and the extreme differential analysis of the stepped nozzle outlet speed is shown in Supplementary Table S1, where *K*_*i*_ is the average of the solution outlet speed of a stepped nozzle at i level. The *K*_*i*_ value can judge the merits of this factor at *i* level; *R* is the difference between the maximum and the minimum of the three levels of a ladder nozzle factor *K*_*i*_ value; and the *R* value size can determine the impact of this factor on the solution outlet speed.

The extreme difference analysis result of the orthogonal test in Supplementary Table S1 shows that the main order of the influence on the solution outlet velocity is DCAB. The stepped nozzle outlet diameter has the greatest impact on the solution outlet velocity, followed by the inlet diameter, total nozzle length, and inlet section length, and the best combination is A_2_B_2_C_3_D_1_ which includes the solution outlet diameter being 20 mm, inlet length being 8 mm, inlet diameter being 14 mm, and outlet diameter being 0.6 mm.

### The Orthogonal and Simulation Optimization of the Conical-Straight Nozzle

The 3D structure of the conical-straight nozzle is shown in Supplementary Figure S8A. The length of the solution container is 60 mm, and the diameter of the container pipe is consistent with the nozzle inlet section. The unstructured grid mesh model of the conical-straight nozzle is shown in Supplementary Figure S8B. The maximum mesh size of the overall mesh is set to 0.1 mm to ensure the mesh division quality at the nozzle and the accuracy of the simulation results. The boundary layer grid structure of the conical-straight nozzle is a hexahedral prismatic grid, and the boundary layer is divided into three layers.

In order to obtain the optimal structural parameters of the conical-straight nozzle, orthogonal tests are required, and the outlet velocity of the spinning solution in the conical-straight nozzle is used as an important indicator. The total nozzle length, inlet section length, inlet diameter, and outlet diameter of the nozzle are indicated by codes A, B, C, and D, respectively, each with three levels. The orthogonal factor level table is also shown in Table 1.

The orthogonal test scheme of nine groups of different parameters can be obtained. The numerical simulation results of the conical nozzle are shown in Table 3.

**TABLE 3:** Results of the orthogonal test of the conical-straight nozzle.

	A	B	C	D	
Test number	Total length (mm)	Inlet length (mm)	Inlet diameter (mm)	Outlet diameter (mm)	Outlet velocity (m/s)
1	15	6	10	0.6	90.6152
2	15	8	12	0.8	80.6852
3	15	10	14	1.0	74.6929
4	20	6	12	1.0	54.3569
5	**20**	**8**	**14**	**0.6**	**201.4941**
6	20	10	10	0.8	54.4203
7	25	6	14	0.8	115.2517
8	25	8	10	1.0	39.6406
9	25	10	12	0.6	140.7652

Note: The bold values represents the best combinations of parameters.

Supplementary Figure S9 shows a cloud map of the solution flow velocity distribution in nine test schemes. During the spinning solution flowing from the conical inlet section to the nozzle outlet, the flow speed of the spinning solution gradually increases, reaching the maximum value at the conical nozzle outlet. Supplementary Figure S10 shows a cloud map of the solution flow velocity distribution at the outlet section of the conical nozzle in the nine test schemes.

The extreme difference analysis of the outlet speed of the spinning solution in nine groups of orthogonal test schemes is required to study the influence of various factors of the conical-straight nozzle on the outlet speed of the spinning solution and the best combination of factors, and the extreme difference analysis results are shown in Supplementary Table S2.

The results show that the influence on the outlet velocity of the spinning solution is DCAB. The nozzle outlet diameter has the greatest impact on the solution outlet speed, followed by the inlet diameter, the nozzle total length, and the inlet section length. The best test combination of the conical nozzle is A_2_B_2_C_3_D_1_, which includes the total length of the nozzle being 20 mm, inlet section length being 8 mm, inlet diameter being 14 mm, and outlet diameter being 0.6 mm, and they were indicated in Tables with bold style.

### The Orthogonal and Simulation Optimization of the Conical Nozzle

The 3D structure and grid model of the conical nozzle are shown in Supplementary Figure S11. The unstructured tetrahedral mesh division with the maximum mesh size of the overall mesh set to 0.1 mm is needed. The boundary layer grid of the conical nozzle is hexahedral prismatic grid, and the boundary layer is divided into three layers.

The outlet velocity of the spinning solution in the conical nozzle is taken as the orthogonal test index, and the total nozzle length, the inlet diameter, and the outlet diameter are used as factors of the orthogonal test, respectively, represented by codes A, B, and C. Each factor has three levels, and the orthogonal factor level table is shown in Table 4. The orthogonal test scheme and numerical calculation results of the conical nozzle are shown in Table 5.

**TABLE 4:** Factors and levels of the orthogonal test of the conical nozzle.

Level	Factor (mm)		
	A	B	C
	Total length	Inlet diameter	Outlet diameter
1	15	10	**0.6**
2	**20**	12	0.8
3	25	**14**	1.0

Note: The bold values represents the best combinations of parameters.

**TABLE 5:** Results of the orthogonal test of the conical nozzle.

Test number	A	B	C	Outlet velocity (m/s)
	Total length (mm)	Inlet diameter (mm)	Outlet diameter (mm)	
1	15	10	0.6	92.1146
2	15	12	1.0	54.1519
3	15	14	0.8	117.1940
4	20	10	1.0	38.3559
5	20	12	0.8	81.8241
6	**20**	**14**	**0.6**	**203.7750**
7	25	10	0.8	55.2153
8	25	12	0.6	141.6399
9	25	14	1.0	76.4016

Note: The bold values represents the best combinations of parameters.

Supplementary Figure S12 shows a cloud map of the solution flow velocity distribution in the conical nozzle in the nine test schemes. The flow rate of the spinning solution flowing from the nozzle inlet to the nozzle outlet gradually increases, with the maximum speed at the nozzle outlet. Supplementary Figure S13 shows a cloud map of the solution flow velocity distribution at the outlet section of the conical nozzle. It can be seen that the flow velocity distribution at the outlet is uneven during the nozzle rotating at high speed.

The extreme differential analysis of the outlet speed of the spinning solution in nine orthogonal test schemes is required to study the influence of the factors of the conical nozzle on the outlet speed and the best combination of factors. The extreme differential analysis results are shown in Supplementary Table S3, and the results show that the order of the influence of the solution outlet velocity is CBA. The conical nozzle outlet diameter has the greatest impact on the solution outlet velocity, followed by the inlet diameter, and the inlet section length. The best structural parameters of the conical nozzle are A_2_B_3_C_1_, which includes the total length being 20 mm, nozzle inlet diameter being 14mm, and nozzle outlet diameter being 0.6 mm.

### The Orthogonal and Simulation Optimization of the Curved-Tube Nozzle

The 3D structure of the curved-tube nozzle is shown in Supplementary Figure S14A. The length of the solution container is 60 mm, and the diameter of the container is consistent with the nozzle inlet. The curved-tube nozzle mesh model is shown in Supplementary Figure S14B. The unstructured grid division is adopted, and the grid structure is a tetrahedral mesh. The maximum mesh size of the overall grid is set to 0.1 mm. The boundary layer grid structure of the curved nozzle is a hexahedral prismatic grid, and the boundary layer is divided into three layers.

The total length of the curved-tube nozzle is 20 mm, and the length of the inlet section, inlet diameter, outlet diameter, bent radius of the curvature, and bent core angle are selected as five factors of orthogonal test design, represented by A, B, C, D, and E, respectively. Each factor has four levels, as shown in Table 6. Taking the outlet velocity of the spinning solution in the curved tube nozzle as the test index, the L_16_(4^5^) orthogonal test design form is selected; 16 groups of test schemes were obtained; and numerical calculation results are shown in Table 7.

**TABLE 6:** Factors and levels of the orthogonal test of the curved-tube nozzle.

Level	Factor				
	A	B	C	D	E
	Inlet length (mm)	Inlet diameter (mm)	Outlet diameter (mm)	Bent radius (mm)	Bent core angle (°)
1	4	10	**0.6**	5	30
2	6	12	0.8	6	**45**
3	8	14	1.0	**7**	60
4	**10**	**16**	1.2	8	75

Note: The bold values represents the best combinations of parameters.

**TABLE 7:** Results of the orthogonal test of the curved-tube nozzle.

Test number	A	B	C	D	E	Outlet velocity (m/s)
1	4	10	0.6	5	30	79.2964
2	4	12	0.8	6	45	66.9487
3	4	14	1.0	7	60	57.4808
4	4	16	1.2	8	75	51.0895
5	6	10	0.8	7	75	33.0431
6	6	12	0.6	8	60	124.7408
7	6	14	1.2	5	45	41.0784
8	6	16	1.0	6	30	91.3322
9	8	10	1.0	8	45	25.4458
10	8	12	1.2	7	30	32.4417
11	8	14	0.6	6	75	182.1220
12	8	16	0.8	5	60	138.1333
13	10	10	1.2	6	60	15.5535
14	10	12	1.0	5	75	33.1257
15	10	14	0.8	8	30	105.3173
16	**10**	**16**	**0.6**	**7**	**45**	**260.3505**

The flow velocity distribution of the curved-tube nozzle in combination of different structural parameters in the 16 sets of test schemes is shown in Supplementary Figure S15. The flow rate in the curved-tube nozzle is increasing from the right inlet to the left outlet.

The flow velocity distribution at the curved nozzle outlet in the 16 test schemes is shown in Supplementary Figure S16. The nozzle outlet section flow distributions of Test 1, Test 7, Test 8, Test 10, and Test 15 are even, and the flow velocity area of the spinning solution is close to the center of the nozzle outlet, indicating that the bending nozzle with a center angle of 30 and 45° is easy to form a stable spinning jet. According to Test 3, Test 4, Test 6, Test 6, Test 11, and Test 12, the high-speed area of the nozzle outlet flow velocity with a core angle of 60 and 75° tends to the nozzle outlet pipe wall, which causes the flow velocity distribution uneven and is difficult to sustain the stability of the spinning jet.

The extreme differential analysis of the outlet speed of the spinning solution in the 16 orthogonal test schemes is required to study the influence degree of the curve-tubed nozzle on the outlet speed of the spinning solution and the best combination of factors, and the extreme differential analysis results are shown in Supplementary Table S4.

The extreme differential analysis results of the orthogonal test of the curved nozzle show that the order of the test factors on the solution outlet velocity is CBAED. The outlet diameter of the curved nozzle has the greatest influence on the outlet speed of the spinning solution, followed by the inlet diameter, the length of the inlet section, the corner of the bent, and the radius of the curvature. The best combination of the curved nozzle is A_4_B_4_C_1_D_3_E_2_, which includes the total length being 20 mm, nozzle inlet section being 10mm, inlet diameter being 16 mm, outlet diameter being 0.6 mm, the radius of curvature of the bent being 7 mm, and bent core angle being 45°, and the spinning solution has the maximum outlet velocity in the curved nozzle.

In this part, according to the orthogonality, some representative points that had the characteristics of “evenly dispersed, neat, and comparable” were selected from the comprehensive test. By using the orthogonal test method, the time and cost of the test can be reasonably reduced, and the effectiveness of the test case is improved.

## High-Speed Centrifugal Spinning Experiment

### High-Speed Centrifugal Spinning Equipment and Preparation of Spinning Solution

**FIGURE 5:**
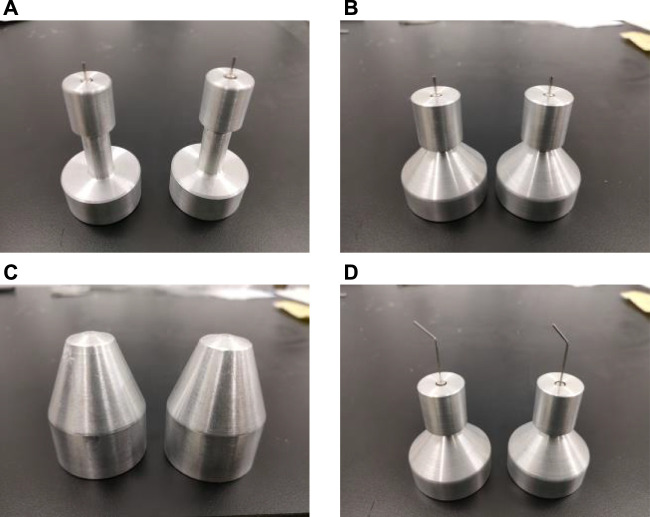
High-speed centrifugal spinning nozzles. **(A)** Stepped nozzle. **(B)** Conical-straight nozzle. **(C)** Conical nozzle. **(D)** Curved-tube nozzle.

According to the optimization results of different centrifugal spinning nozzle, the optimal structural parameters of the stepped, conical-straight, conical, and curved-tube nozzles are obtained, as shown in Table 8, and the high-speed centrifugal spinning nozzles combining the optimal structural parameters are shown in Figure 5. All four nozzles were made of aluminum alloy with anti-oxidation and corrosion resistance and machined on the lathe.

**TABLE 8:** Structural parameters of centrifugal spinning nozzles.

	Stepped nozzle	Conical-straight nozzle	Conical nozzle	Curved-tube nozzle
L_2_ (mm)	20	20	20	20
L_1_ (mm)	8	8	—	10
D_1_ (mm)	14	14	14	16
D_2_ (mm)	0.6	0.6	0.6	0.6
θ (°)	—	—	—	45
R (mm)	—	—	—	7

**FIGURE 6:**
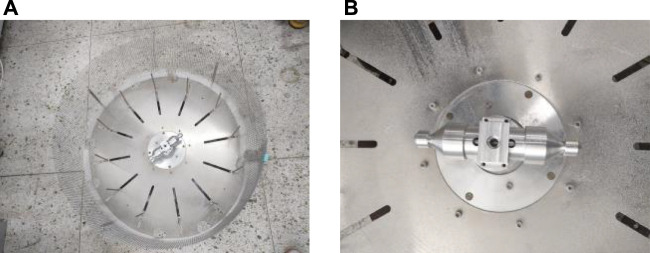
High-speed centrifugal spinning device and spinneret. **(A)** High-speed centrifugal spinning device; **(B)** centrifugal spinning spinneret.

The high-speed centrifugal spinning device used in the experiment is independently developed and assembled, mainly composed of drive motor, drive mechanism, spinneret, and collection device, as shown in Figure 6A. The spinneret is composed of a solution container and two spinning nozzles, as shown in Figure 6B. Polyvinyl oxide (PEO) is a water-soluble, crystalline, and thermoplastic polymer which is completely soluble in water and non-toxic and non-stimulating and has less environmental pollution. The PEO aqueous solution has high viscosity and good spinnability at the low concentration, thus becoming a common spinning material for high-speed centrifugal spinning. The high-speed centrifugal spinning experiment prepares nanofiber with PEO solution, with full stirring through a magnetic stirrer at normal temperature and normal pressure, with an average stirring time of 6 h, and the stirring time is slightly extended with the increase of PEO solution concentration.

This article conducts the high-speed centrifugal spinning experiment with stepped, conical-straight, conical, and curved-tube nozzles and compares the morphology and diameter distribution of fibers fabricated by different nozzles under scanning electron microscope (SEM) to determine the optimal nozzle structure.

### The Comparison of Fiber Preparation at Different Spinning Solution Concentration

The high-speed centrifugal spinning experiment was conducted at 4000 rpm with different PEO solution concentration, and the morphology and quality of the nanofibers prepared from the four centrifugal spinning nozzles were observed and compared. Due to the low concentration of the PEO spinning solution, the viscosity force between the solutions cannot guarantee the stability of the spinning jet in the air, when the concentration of the PEO solution is 2wt%. Therefore, all the stepped, conical-straight, conical, and curved nozzles could not produce polymer fibers.

**FIGURE 7:**
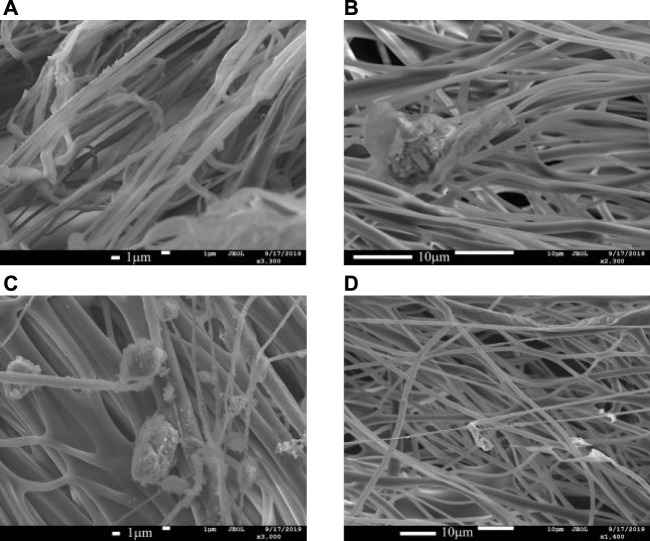
SEM diagram of nanofibers fabricated by different nozzles with a concentration of 4wt.%. **(A)** Stepped nozzle. **(B)** Conical-straight nozzle. **(C)** Conical nozzle. **(D)** Curved-tube nozzle.

When high-speed centrifugal spinning is performed using a PEO solution of 4wt% concentration, all the four centrifugal spinning nozzles began to produce fiber filaments. The scanning electron microscope (SEM) images of PEO nanofibers prepared with different centrifugal spinning nozzles are shown in Figure 7. Fiber masses formed by fiber tangles appeared in the polymer fibers prepared from the stepped nozzle; spherical fibers appeared in the polymer fibers prepared from conical- straight and conical nozzles; the polymer fibers prepared by the curved nozzle showed a tangled fiber mass. The diameter of these clumps and spherical fibers is much larger than the diameter of single fibers, resulting in an uneven distribution of polymer fiber diameter, reducing the production quality and application functionality of nanofibers.

**FIGURE 8:**
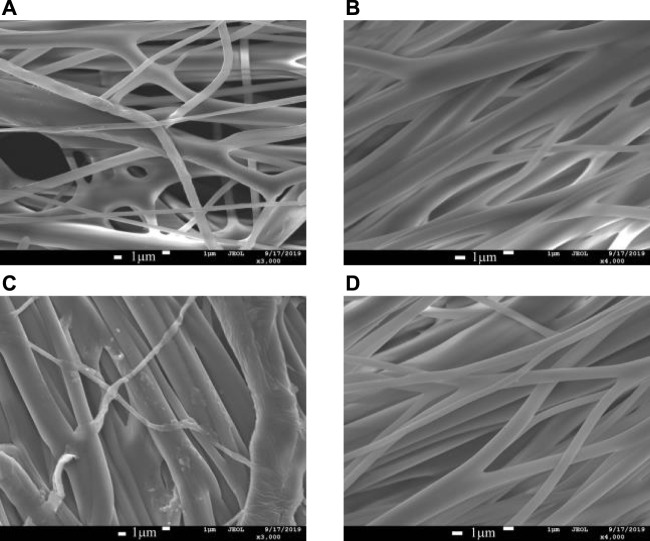
SEM diagram of nanofibers fabricated by different nozzles with a concentration of 6wt.%. **(A)** Stepped nozzle; **(B)** conical-straight nozzle; **(C)** conical nozzle; **(D)** curved-tube nozzle.

When high-speed centrifugal spinning was performed with a PEO solution of 6wt%, the nanofibers prepared from the stepped, straight, conical, and curved nozzle are shown in Figure 8. The morphological quality of nanofibers prepared from four centrifugal spinning nozzles improved, without spherical or mass fiber production. The polymer fiber diameter prepared from the ladder nozzle is evenly distributed with single fiber bifurcation. The nanofibers prepared by conical nozzles have a rough appearance and show a little granular impurity on the fiber surface. The nanofibers prepared by the conical-straight nozzle and the curved-tube nozzle have better morphological quality and better smooth fiber appearance.

### The Comparison of Fibers Prepared at Different Motor Speeds

**FIGURE 9:**
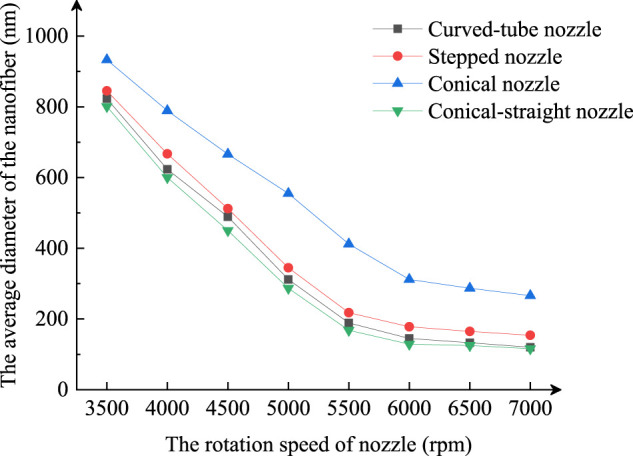
Average diameter of nanofibers fabricated at different rotating speeds.

With other centrifugal spinning experiment parameters unchanged, the motor speed was changed to perform high-speed centrifugal spinning with different centrifugal spinning nozzles to compare the average diameter of nanofibers prepared from different nozzles. Figure 9 shows the average diameter of nanofibers prepared from a stepped, straight, conical, and curved nozzle at different motor speeds.

The average diameter of nanofibers prepared by the centrifugal spinning nozzle decreases with the motor speed. The average diameter of nanofibers prepared by the conical nozzle is much greater than that prepared by stepped, conical-straight, and curved-tube nozzles at different motor speeds. The average diameter of the conical and curved nozzle is not much different. The results show that the nanofiber diameter prepared by the conical-straight nozzle and the curved-tube nozzle is better than that prepared by the stepped nozzle and the conical nozzle.

### The Comparison of Fiber at Different Outlet Diameters

**FIGURE 10:**
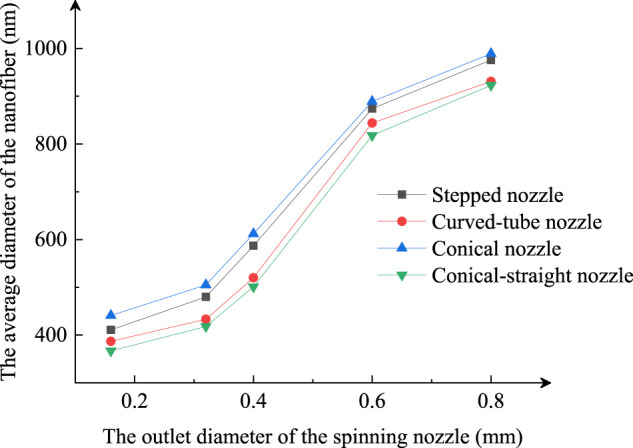
Average diameter of nanofibers fabricated by different outlet diameter of nozzles.

Keeping the other parameters constant and changing the outlet diameter of the nozzle, the average diameter of the nanofibers prepared by different centrifugal spinning nozzles is compared. This experiment performed PEO nanofiber preparation with a spinning solution of 6wt% at 4000 rpm, with the average diameter of the stepped, conical, conical-straight, and curved nozzles at different outlet diameters as shown in Figure 10.

The results show that the smaller the nozzle outlet diameter is, the smaller the average diameter of the prepared nanofibers is. Under the same outlet diameter conditions, the average of the nanofibers prepared by the conical-straight nozzle had the smallest diameter among the other three nozzles.

## Conclusion

This article proposed four different nozzle structures based on the principle of high-speed centrifugal spinning. The relationships between structural parameters of the centrifugal spinning nozzle and the outlet velocity of the spinning solution were obtained through deriving the formula of the outlet velocity. The four centrifugal spinning nozzle structural parameters were optimized using numerical simulation and orthogonal test, and the results are as follows:

(1) The optimal structural parameter combination of the stepped nozzle is: the total length of the nozzle is 20 mm; the inlet section length is 8 mm; the inlet diameter is 14 mm, and the outlet diameter is 0.6 mm.
(2) The optimal structural parameters of the conical-straight nozzle are: the total length of the nozzle is 20 mm; the inlet section length is 8 mm; the inlet diameter is 14 mm, and the outlet diameter is 0.6 mm.
(3) The optimal structural parameters of the conical nozzle are: the total length of the nozzle is 20 mm; the nozzle inlet diameter is 14 mm, and the nozzle outlet diameter is 0.6 mm.
(4) The optimal structural parameters of the curved-tube nozzle are the total nozzle length is 20 mm, inlet section length is 10 mm, inlet diameter is 16 mm, outlet diameter is 0.6 mm, curvature radius of is 7 mm, and bent center angle is 45°.

Finally, the high-speed centrifugal spinning experiment was carried out with four optimized nozzles, and the morphology and diameter distribution of nanofibers were compared. The results showed that the conical-straight nozzle and curved-tube nozzle performed well. The fiber surface was smooth with fewer spherical and mass fibers in the morphological quality of nanofibers. The diameter of fibers prepared by the conical-straight nozzle was minimal, followed by curved-tube nozzles, stepped nozzles, and conical nozzles in the diameter distribution of nanofibers. Therefore, the conical-straight and curved-tube nozzles can be studied further in future research studies.

## Data Availability

The original contributions presented in the study are included in the article/Supplementary Material, further inquiries can be directed to the corresponding author.
