# Supplementary material for: Orthogonal Optimization Research on Various Nozzles of High-Speed Centrifugal Spinning
Source: Front Bioeng Biotechnol. 2022 May 17;10:884316. doi: 10.3389/fbioe.2022.884316 (PMC9152320; doi:10.3389/fbioe.2022.884316)
Supplement: Supplementary file 1 [file DataSheet1.doc]

| **A** 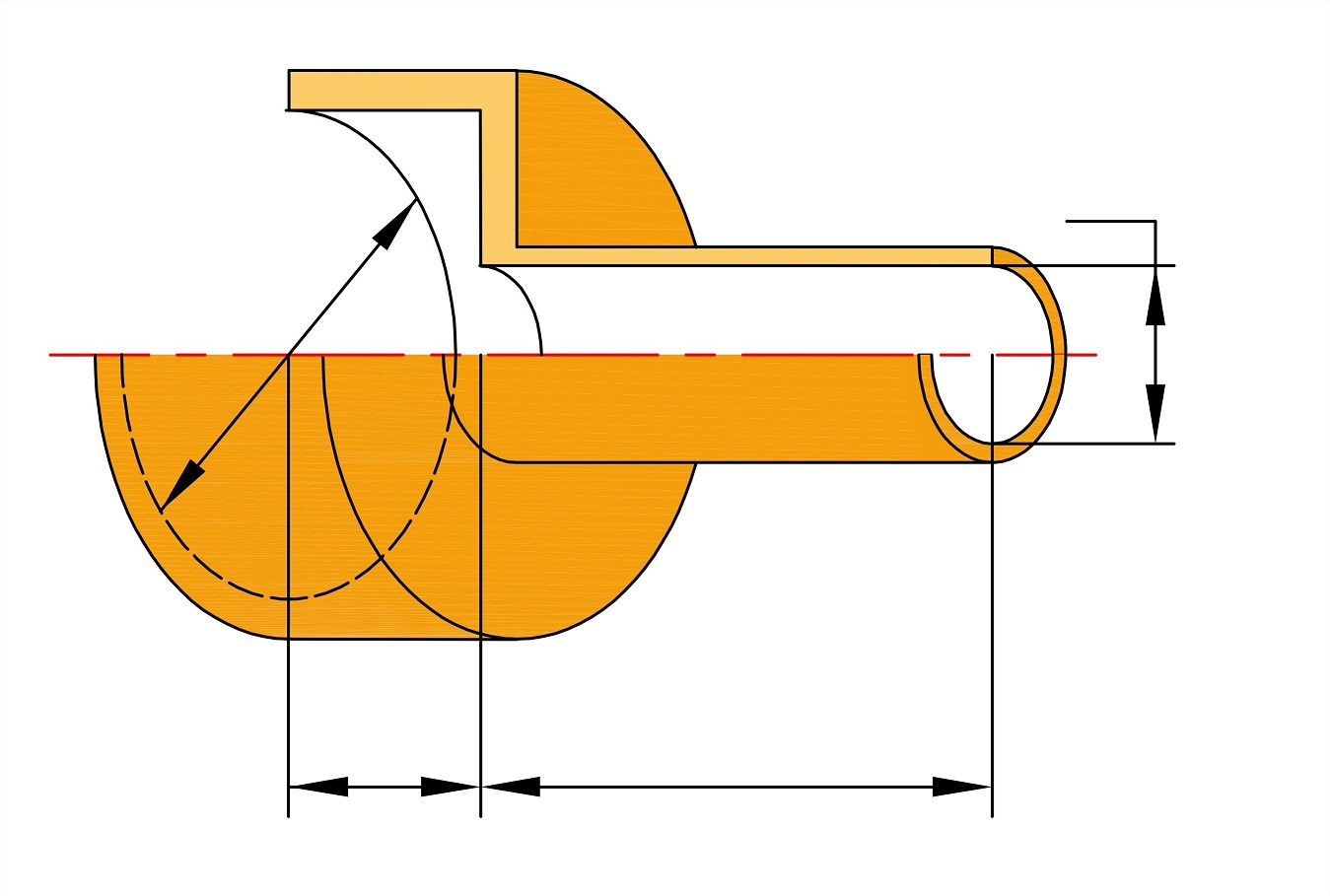 *D_1_*  *L_1_*  *L_2_*  *D_2_* | **B** 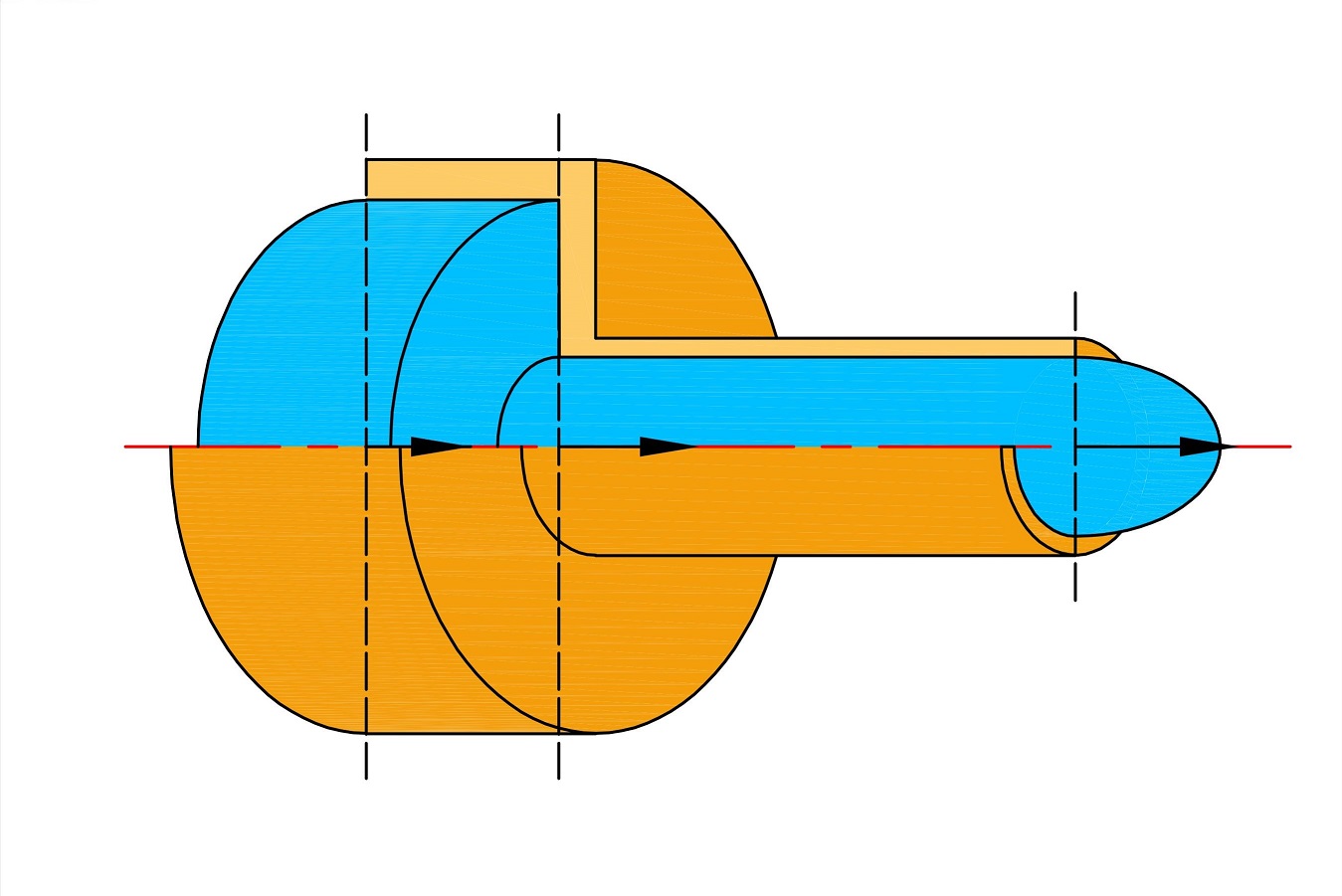 A_1_  *V_1_*  *V_2_*  *V_3_*  A_1_  A_2_  A_2_  A_3_  A_3_ |
| --- | --- |
| **Figure S1 |** The stepped nozzle. **(A)** The structure of stepped nozzle; **(B)** The movement of spinning solution in stepped nozzle | |

| A 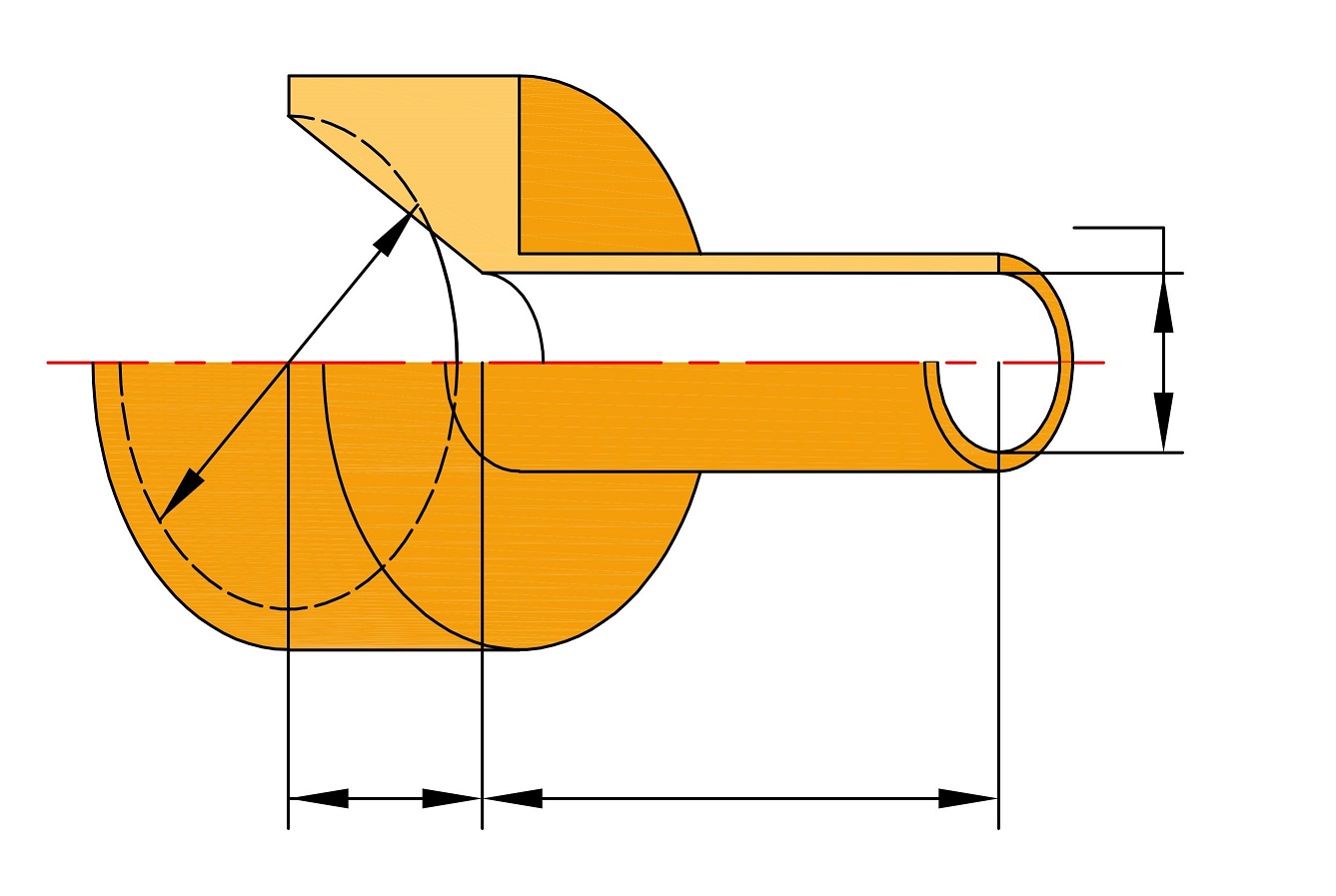 *D_1_*  *L_1_*  *L_2_*  *D_2_* | B 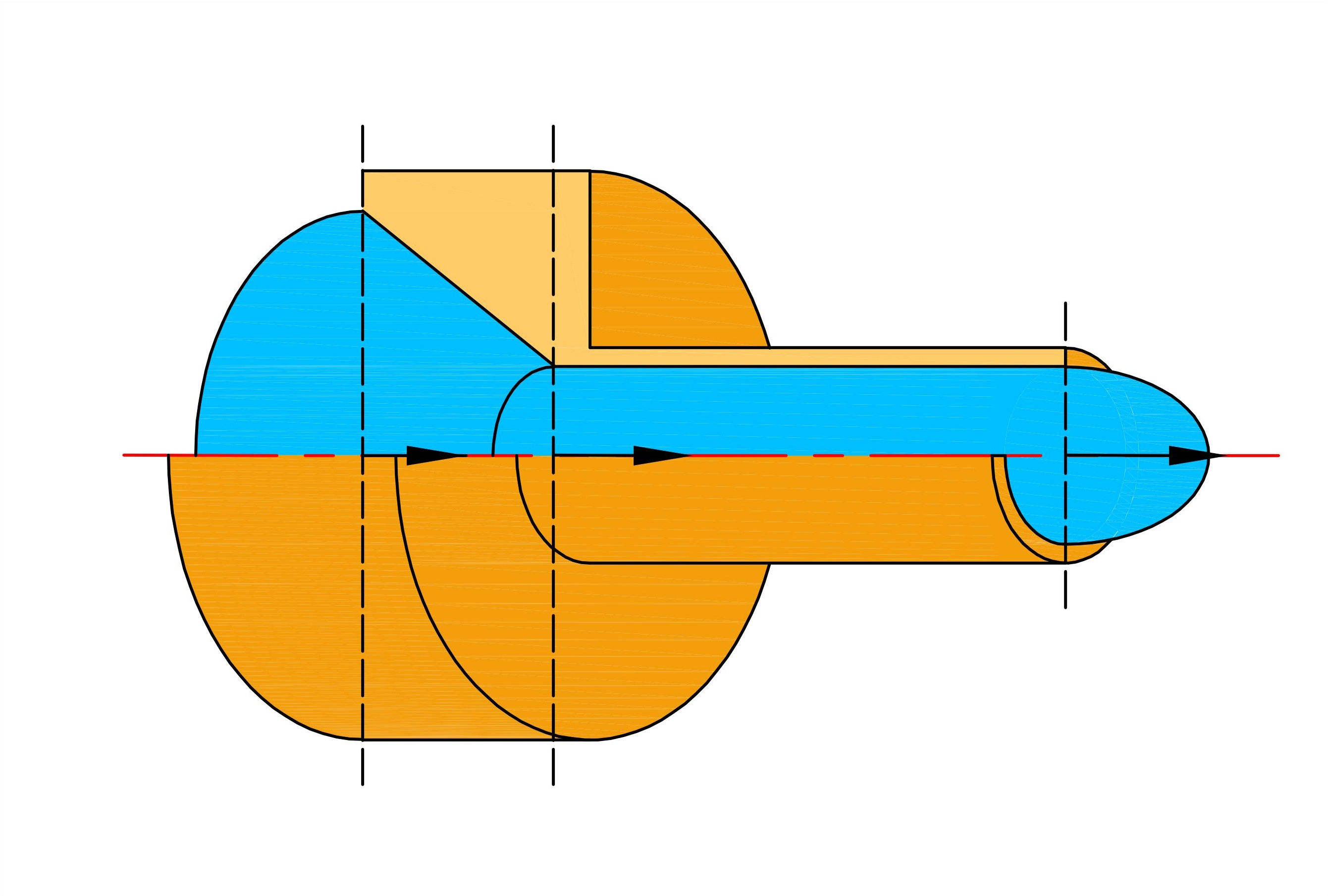 A_1_  *V_1_*  *V_2_*  *V_3_*  A_1_  A_2_  A_2_  A_3_  A_3_ |
| --- | --- |
| **Figure S2 |** The structure of conical-straight nozzle and solution movement in conical-straight nozzle. **(A)** The structure of conical-straight nozzle; **(B)** The solution movement in conical-straight nozzle | |

| **A** 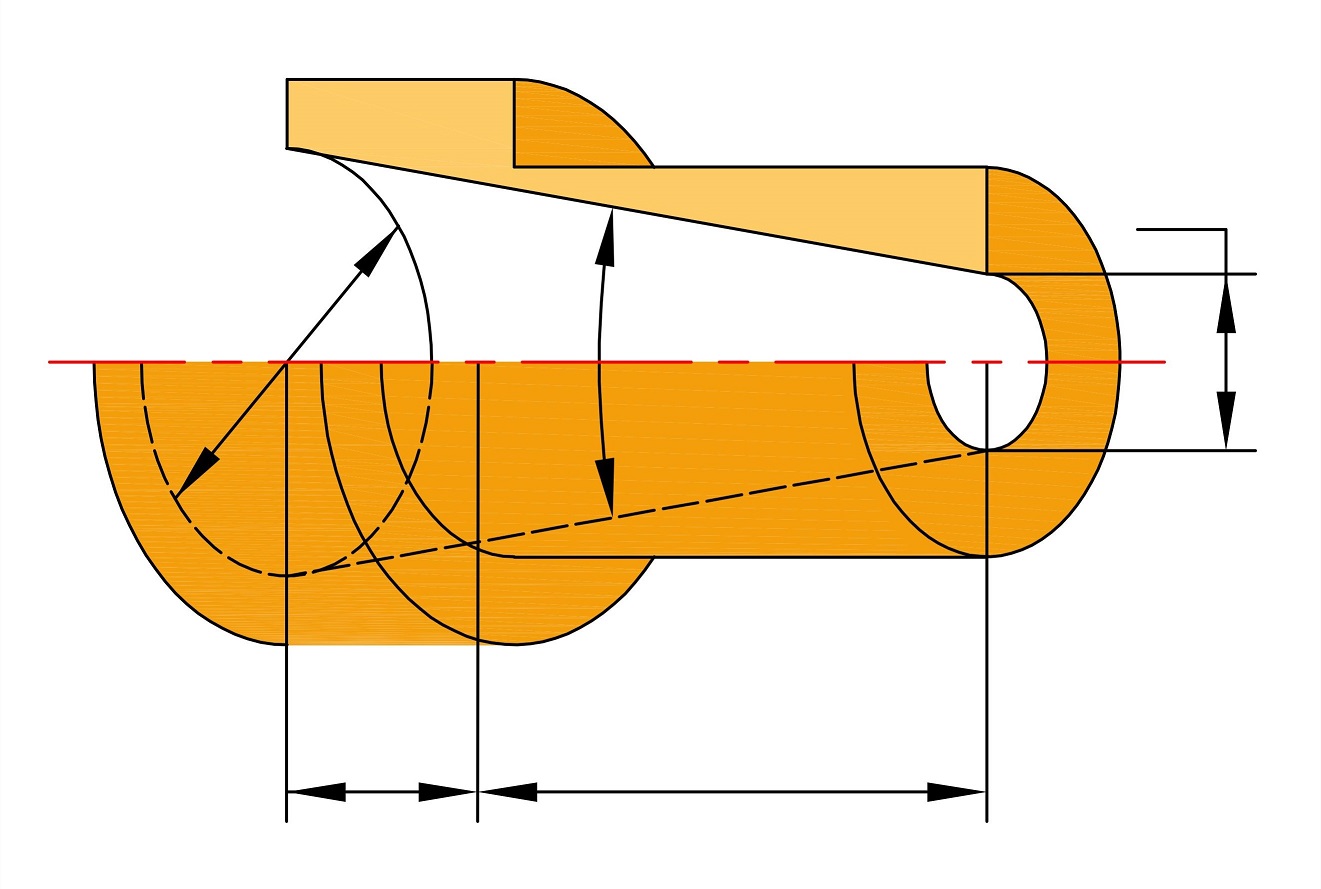 *D_0_*  *L_1_*  *L_2_*  *D_out_* | **B** 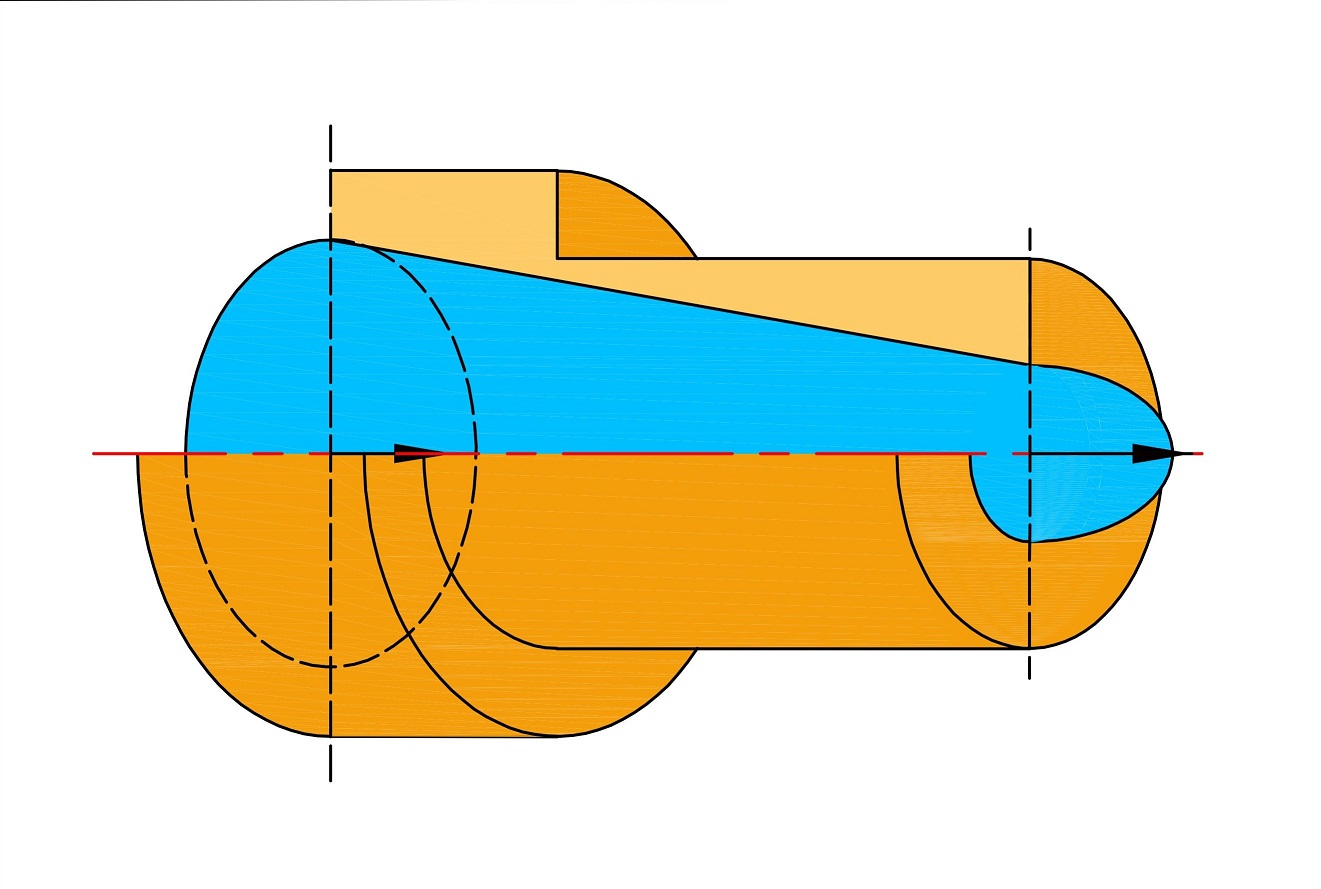 *V_1_*  *A_1_*  *A_2_*  *V_2_*  *A_1_*  *A_2_* |
| --- | --- |
| **Figure S3 |** The structure of conical nozzle and solution movement in conical nozzle. **(A)** The structure of conical nozzle; **(B)** The solution movement in conical nozzle | |

| **A** 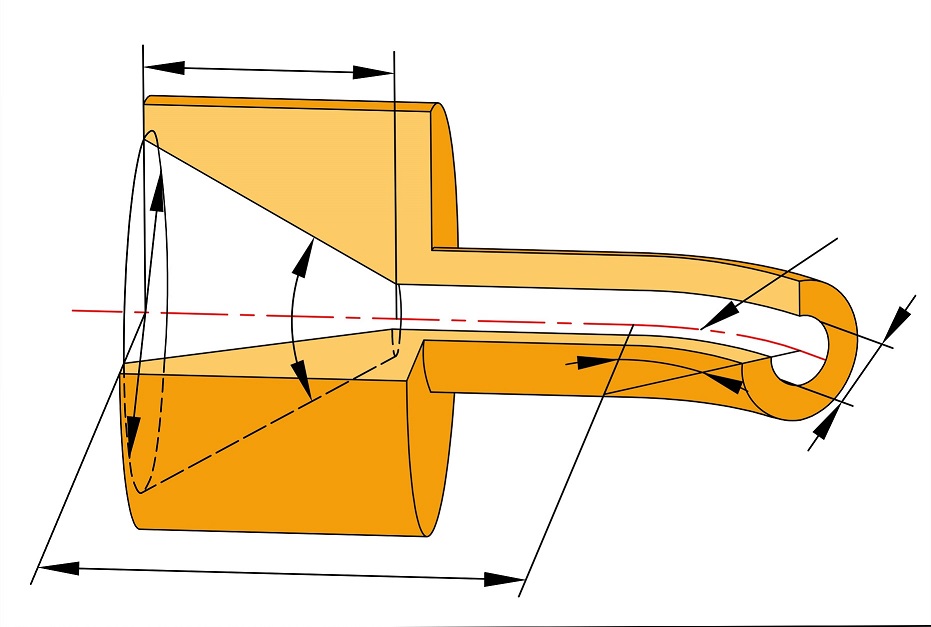 L_1_  D_1_  L_2_  D_2_  R  α  θ | **B** 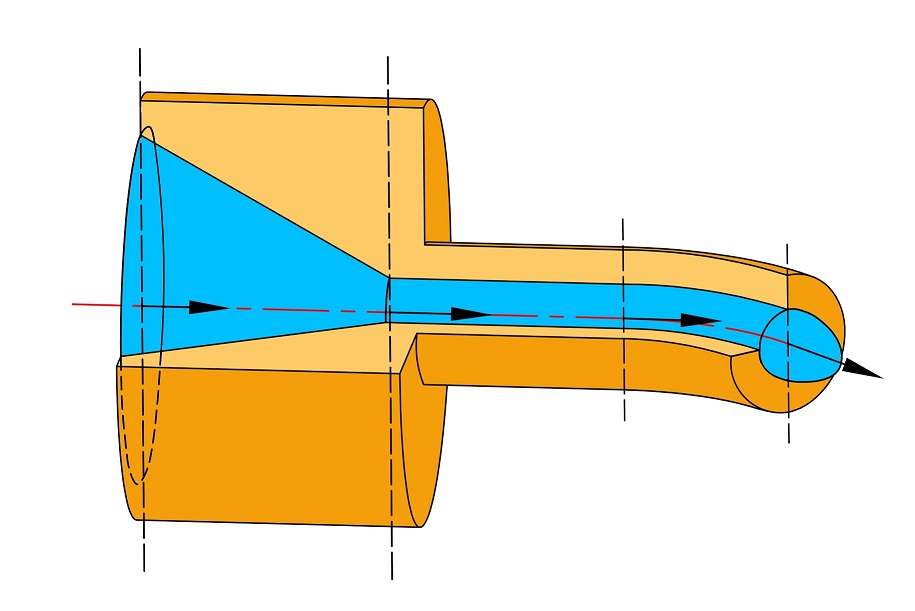 V_3_  A_2_  A_1_  A_2_  A_1_  A_3_  A_3_  A_4_  A_4_  V_4_  V_2_  V_1_ |
| --- | --- |
| **Figure S4 |** The structure of curved-pipe nozzle and solution movement in curved-pipe nozzle. **(A)** The structure of curved-pipe nozzle; **(B)** The solution movement in curved-pipe nozzle | |

| 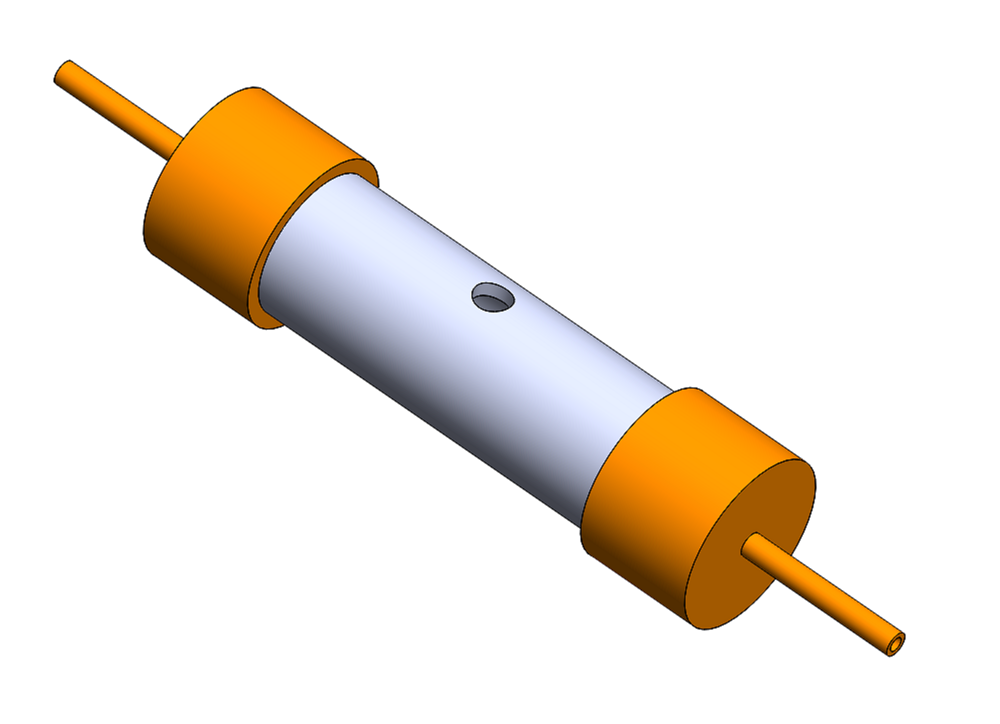 | 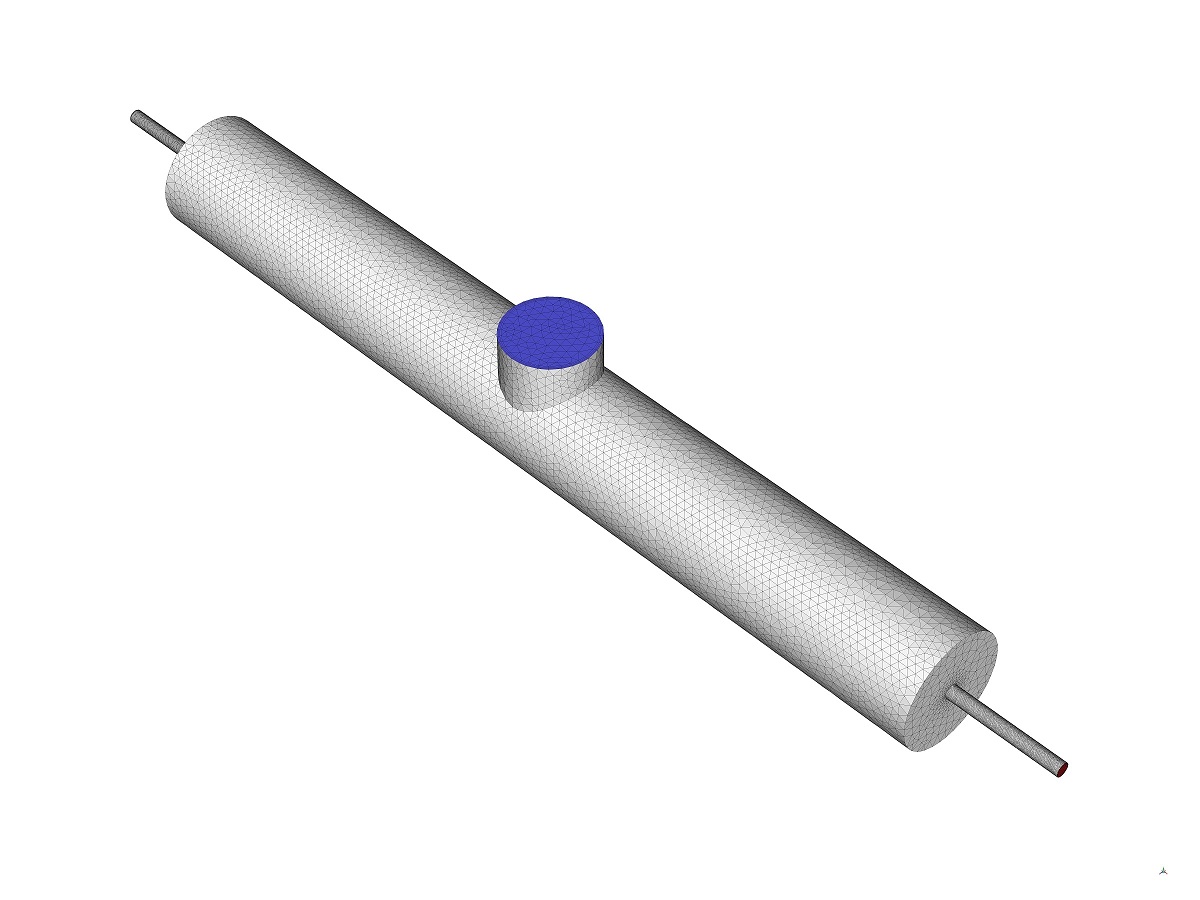 |
| --- | --- |
| **Figure S5 |** The model of the spinneret with stepped nozzles. **(A)** The spinneret with stepped nozzles; **(B)** The mesh division of spinneret with stepped nozzles | |

| 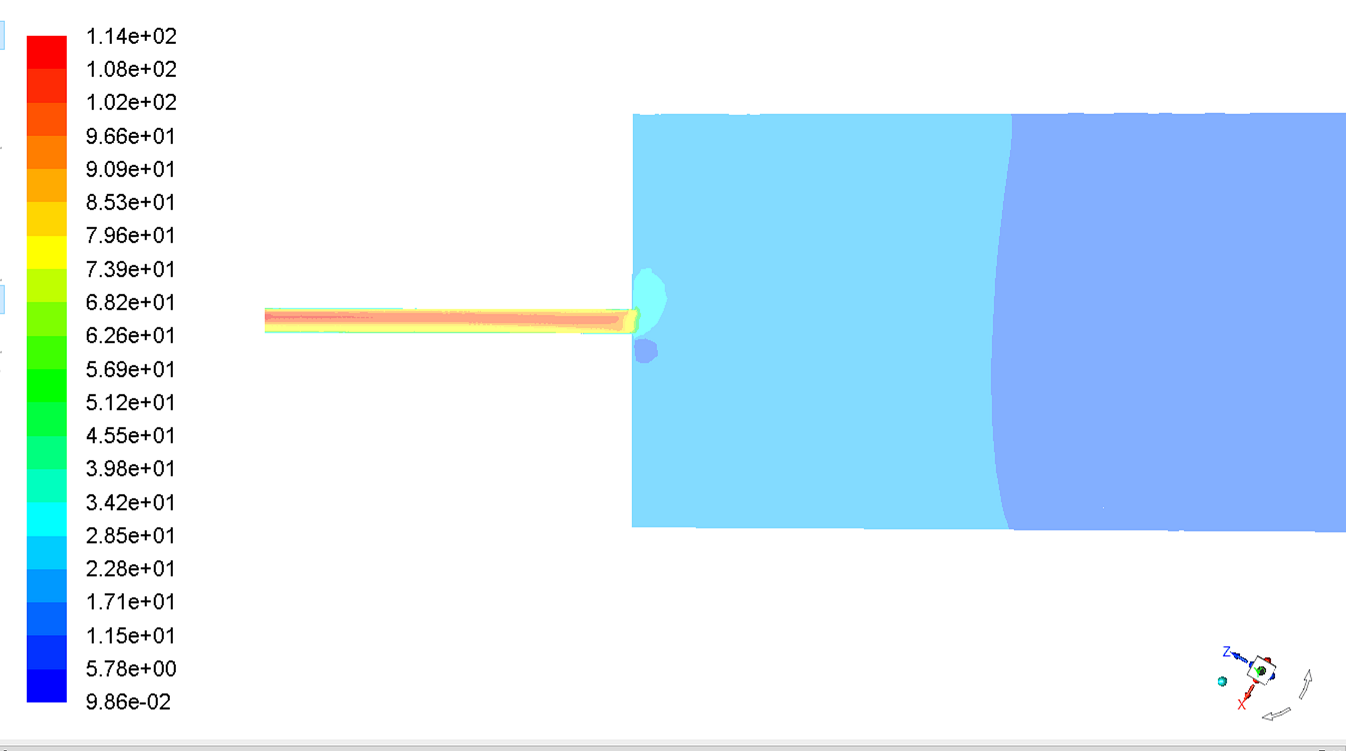  (1) | 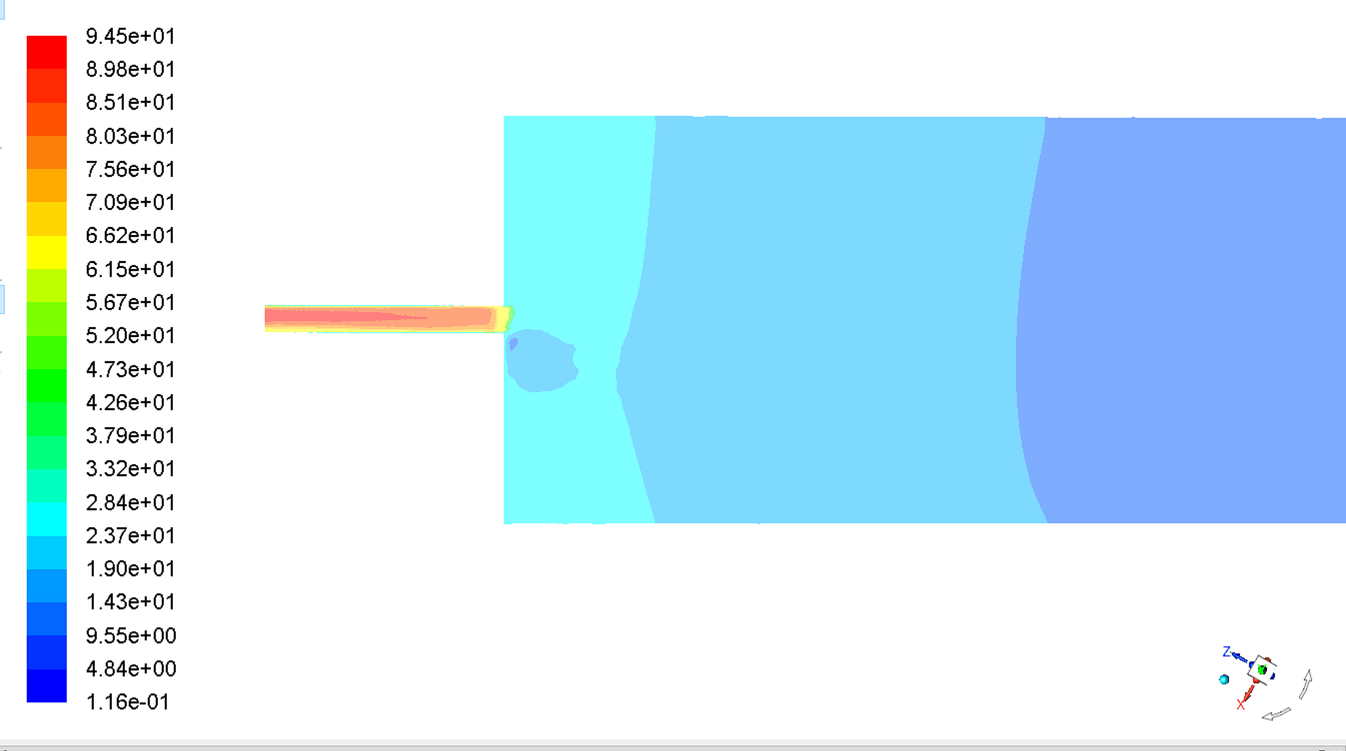  (2) | 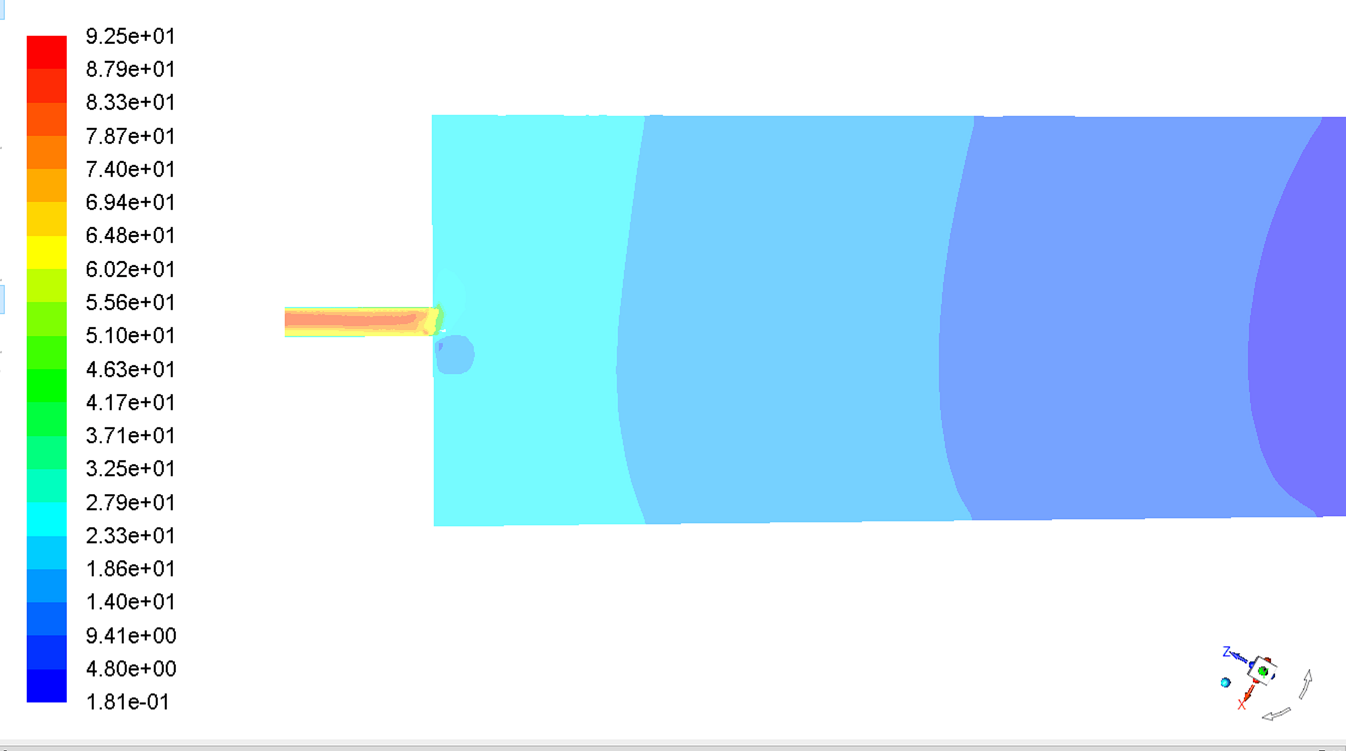  (3) |
| --- | --- | --- |
| 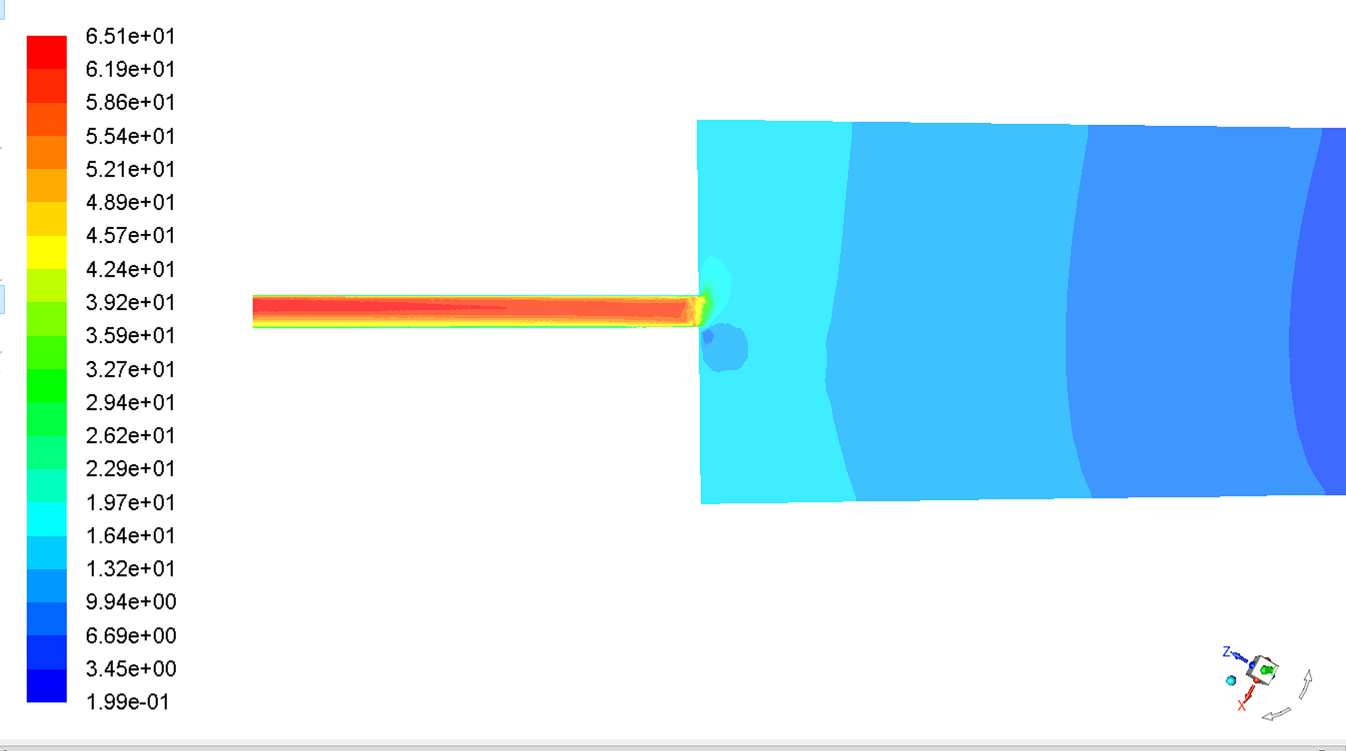  (4) | 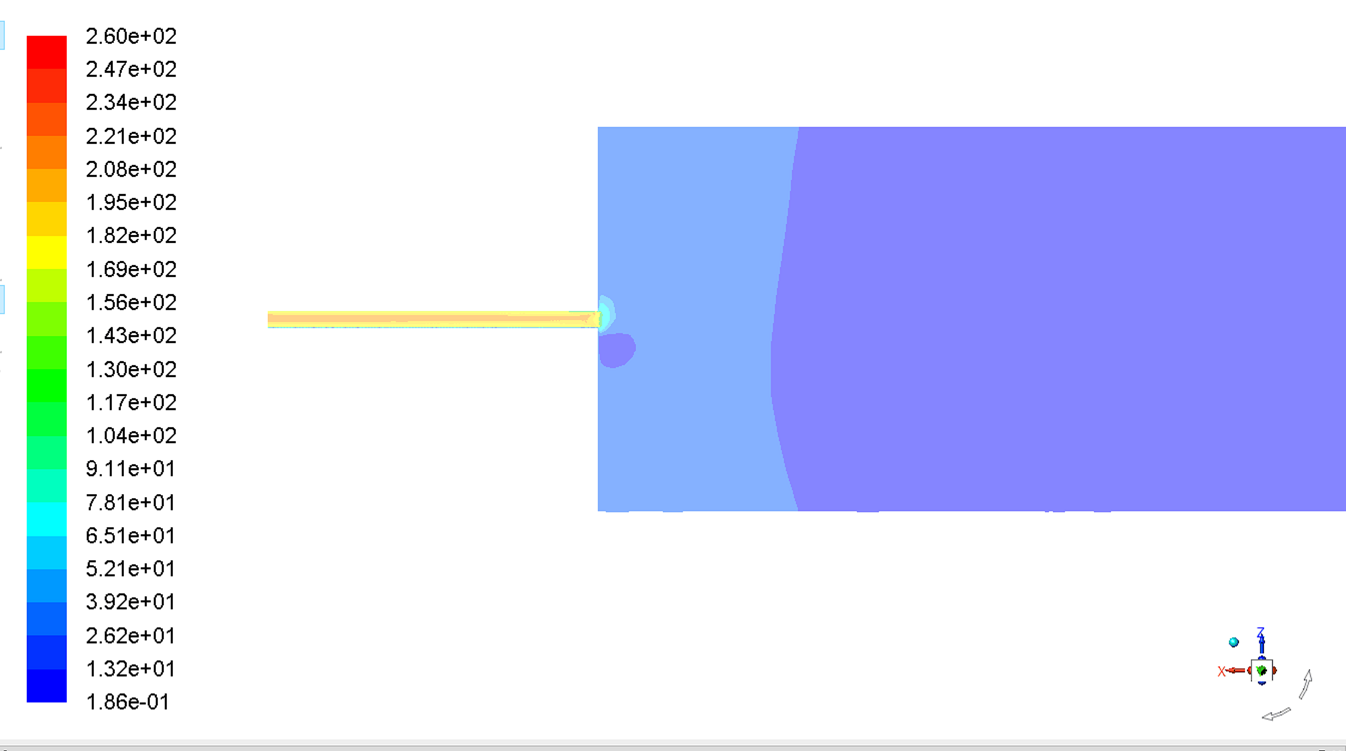  (5) | 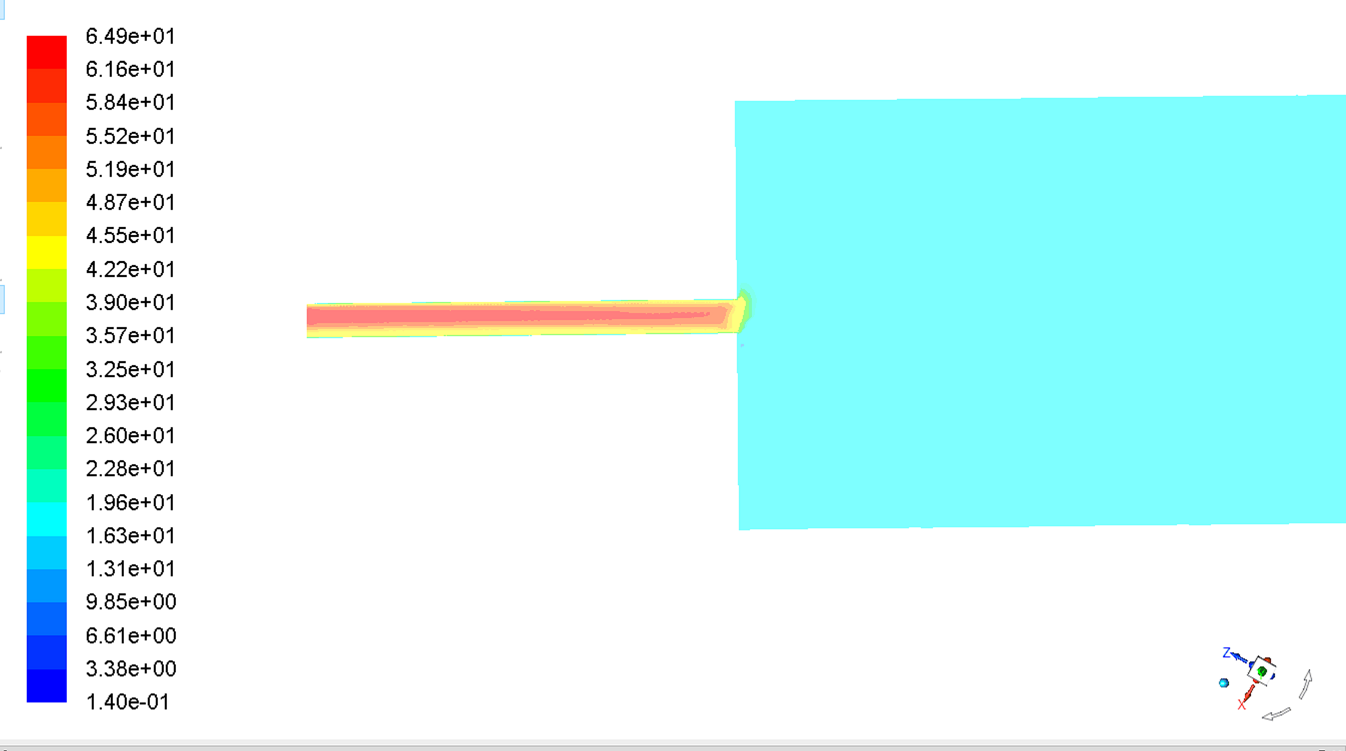  (6) |
| 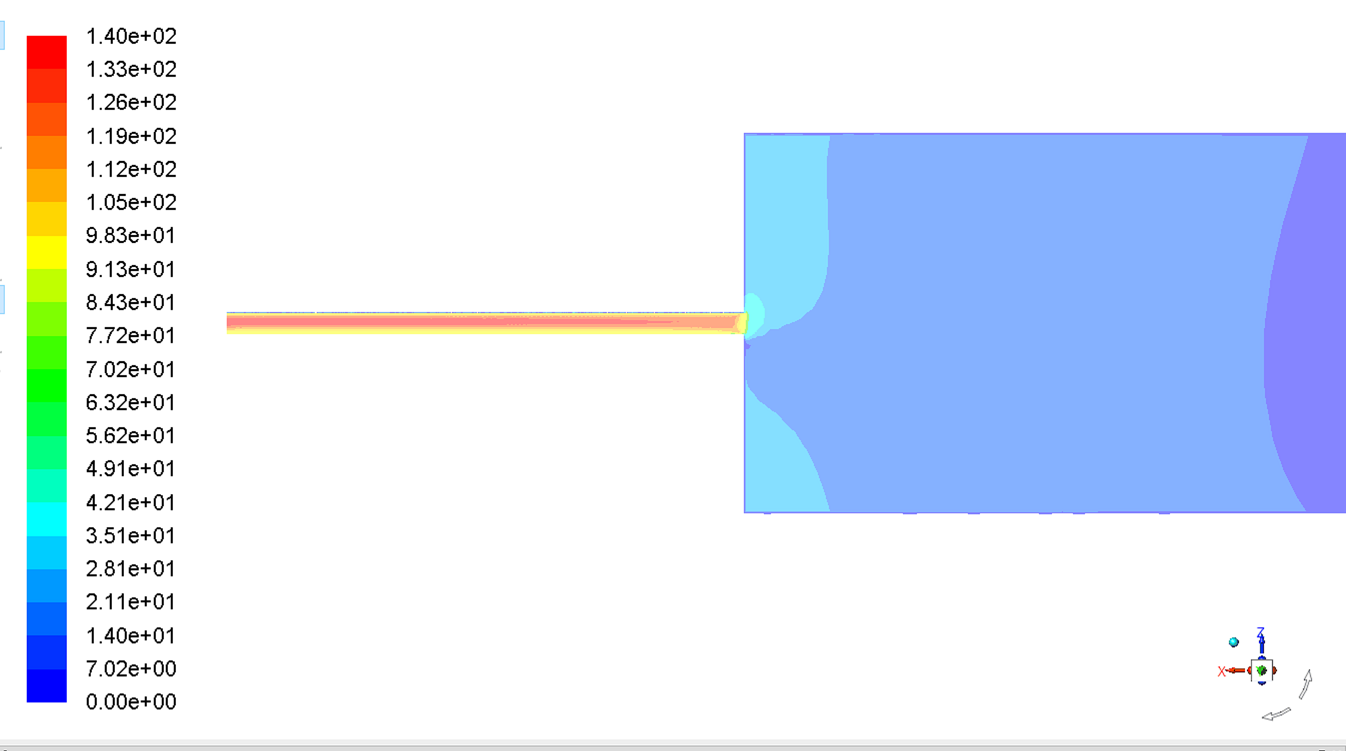  (7) | 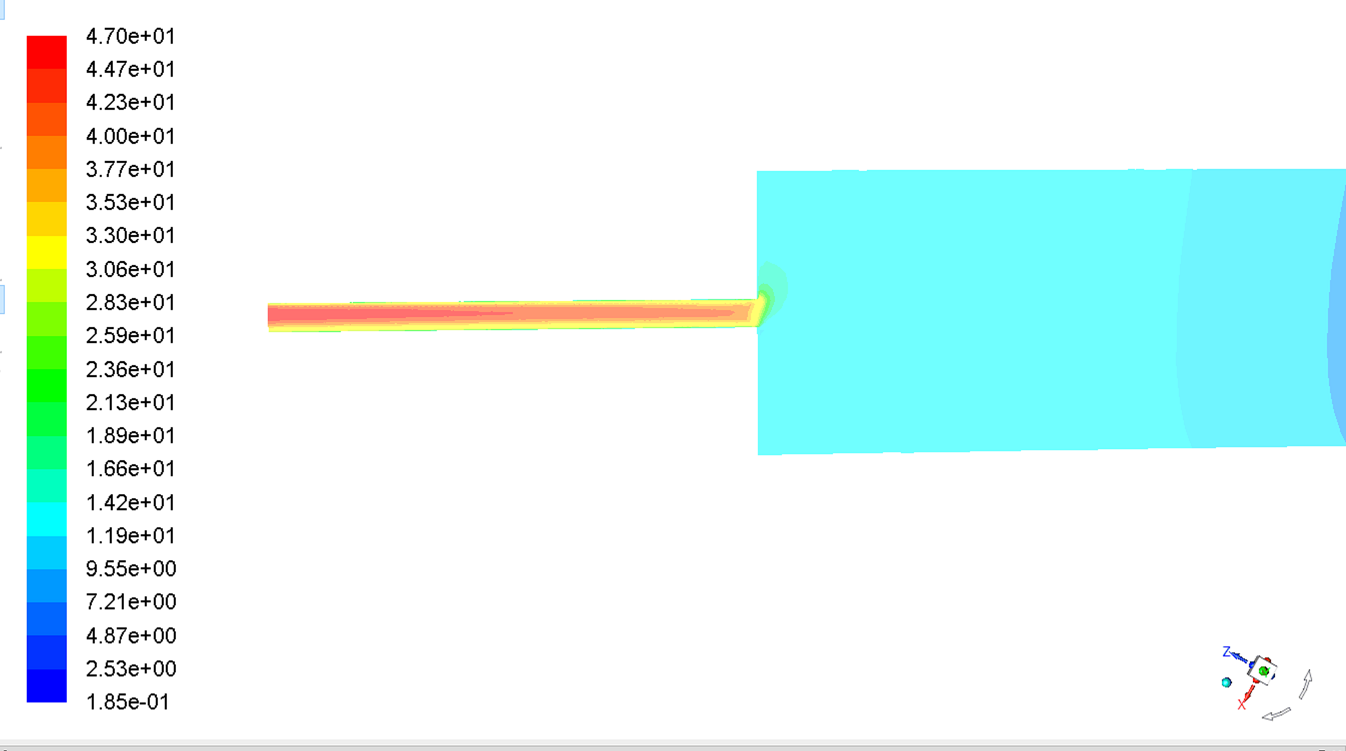  (8) | 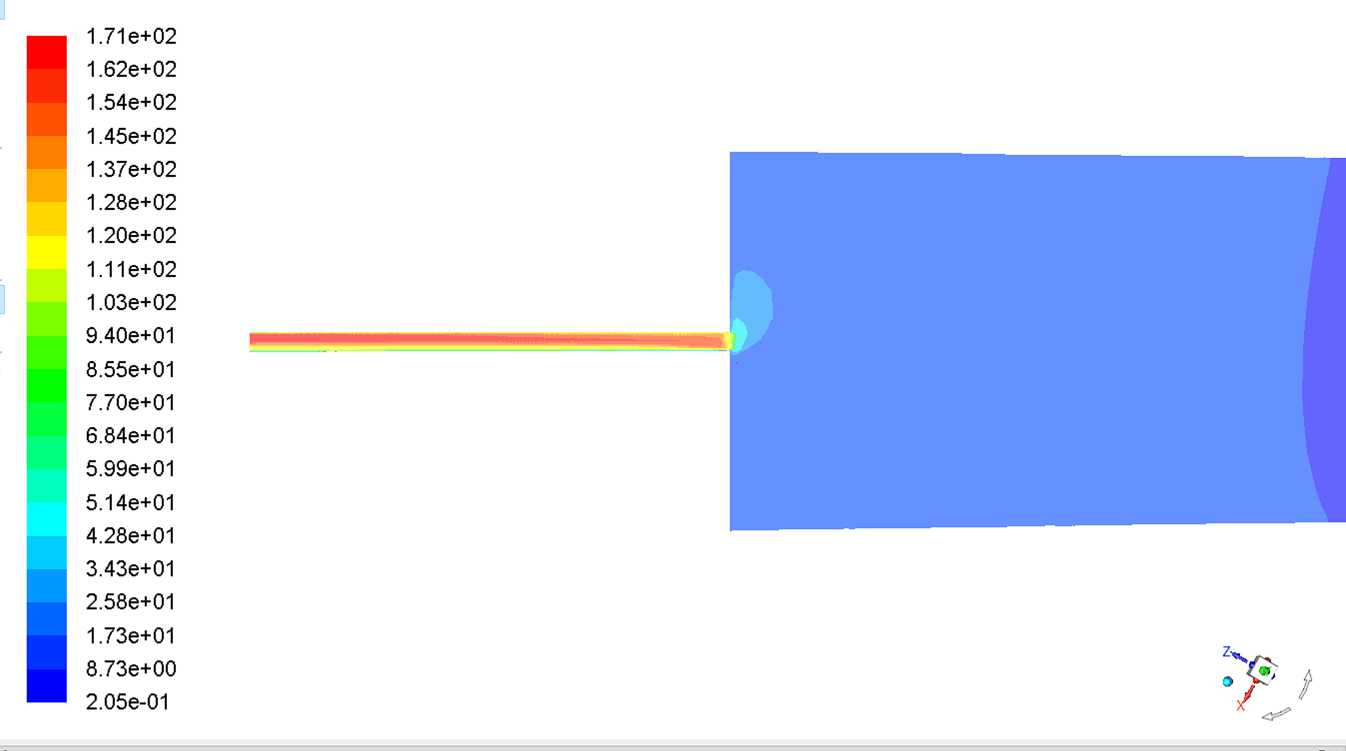  (9) |
| **Figure S6 |** Cloud diagram of solution velocity distribution in stepped nozzles of 9 tests | | |

| 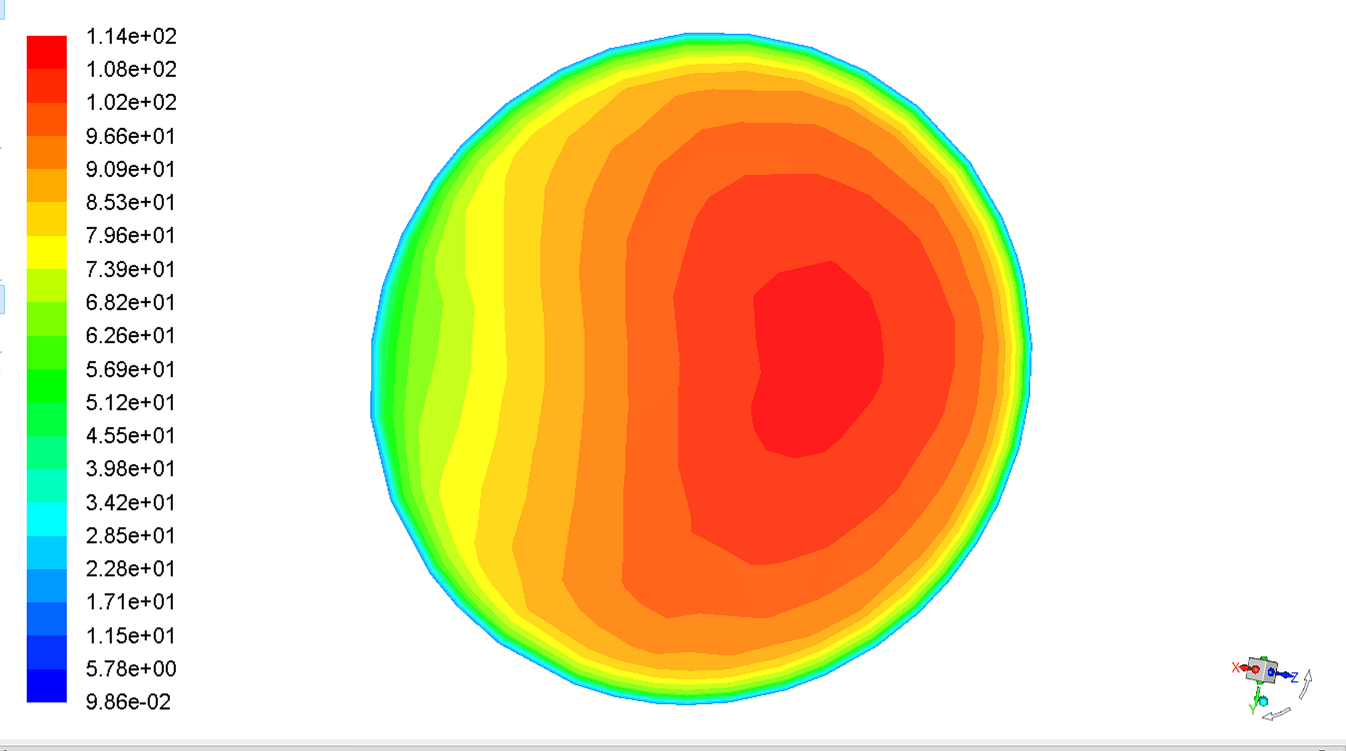  (1) | 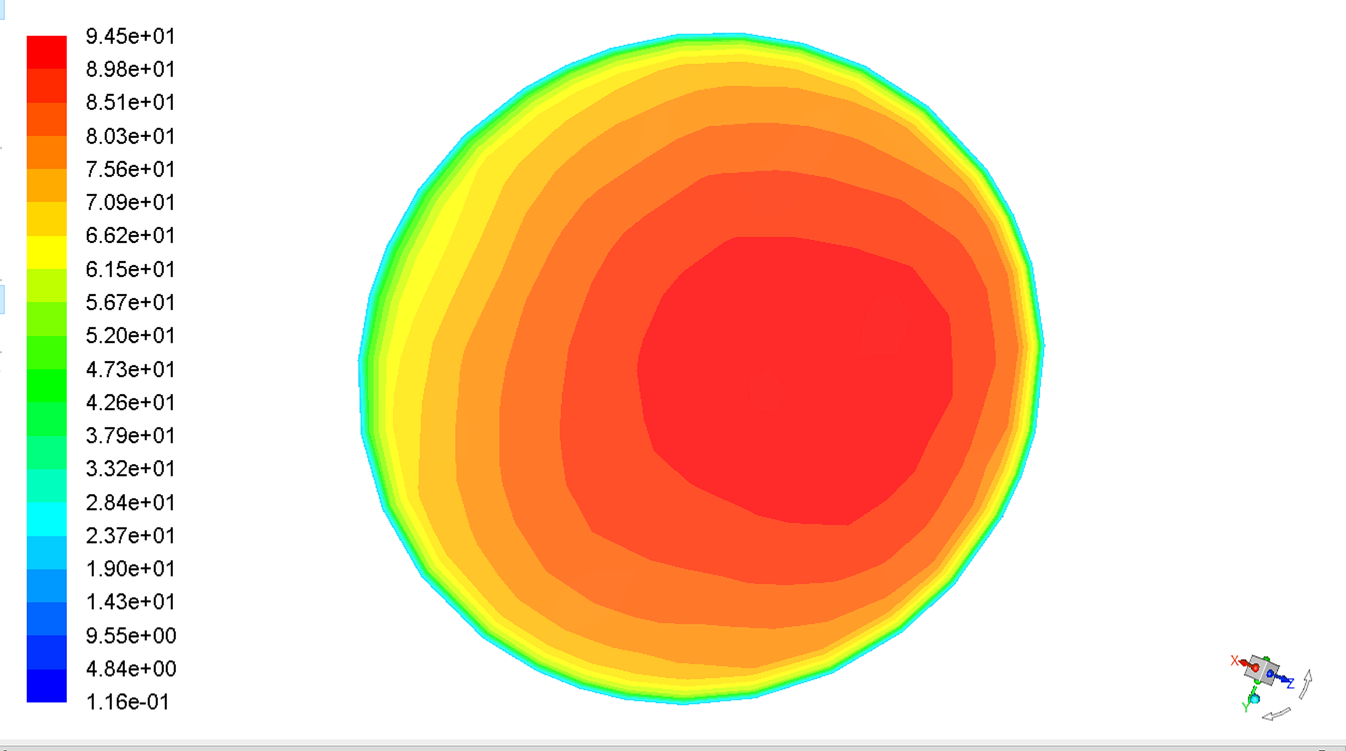  (2) | 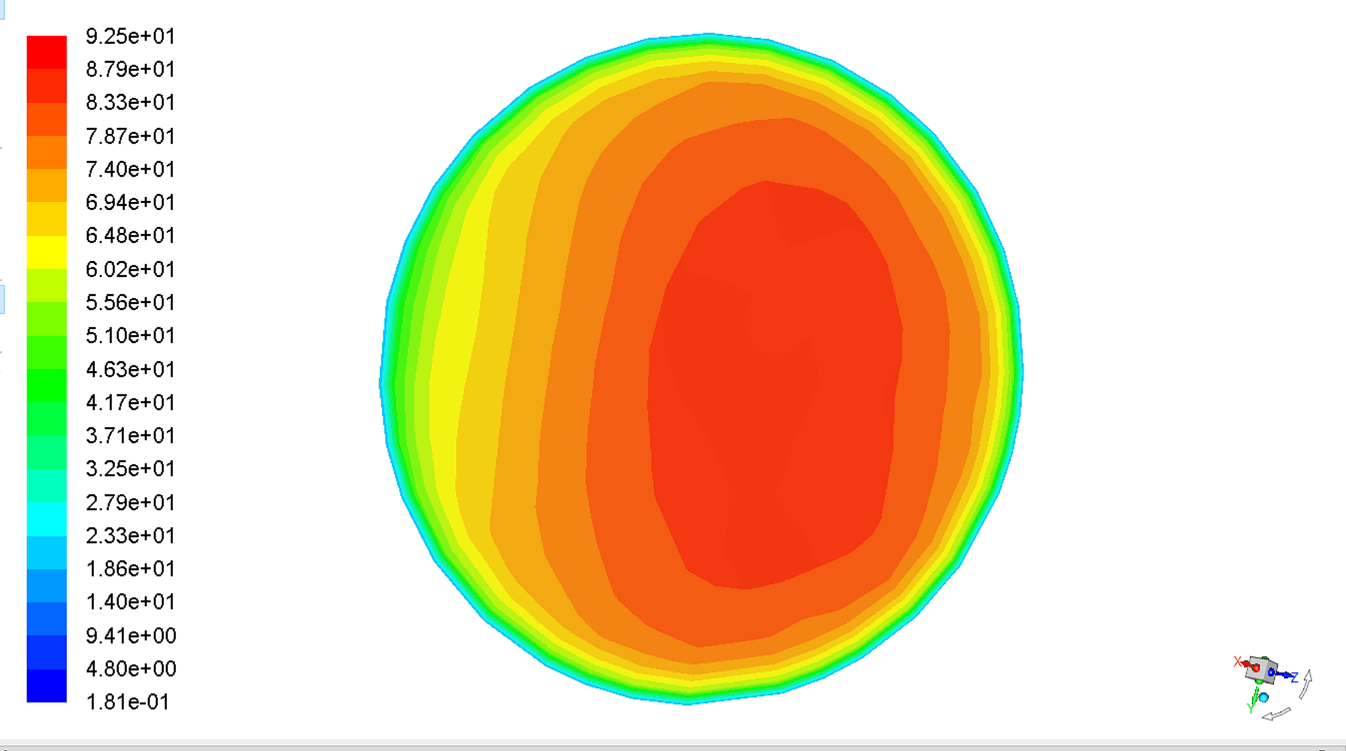  (3) |
| --- | --- | --- |
| 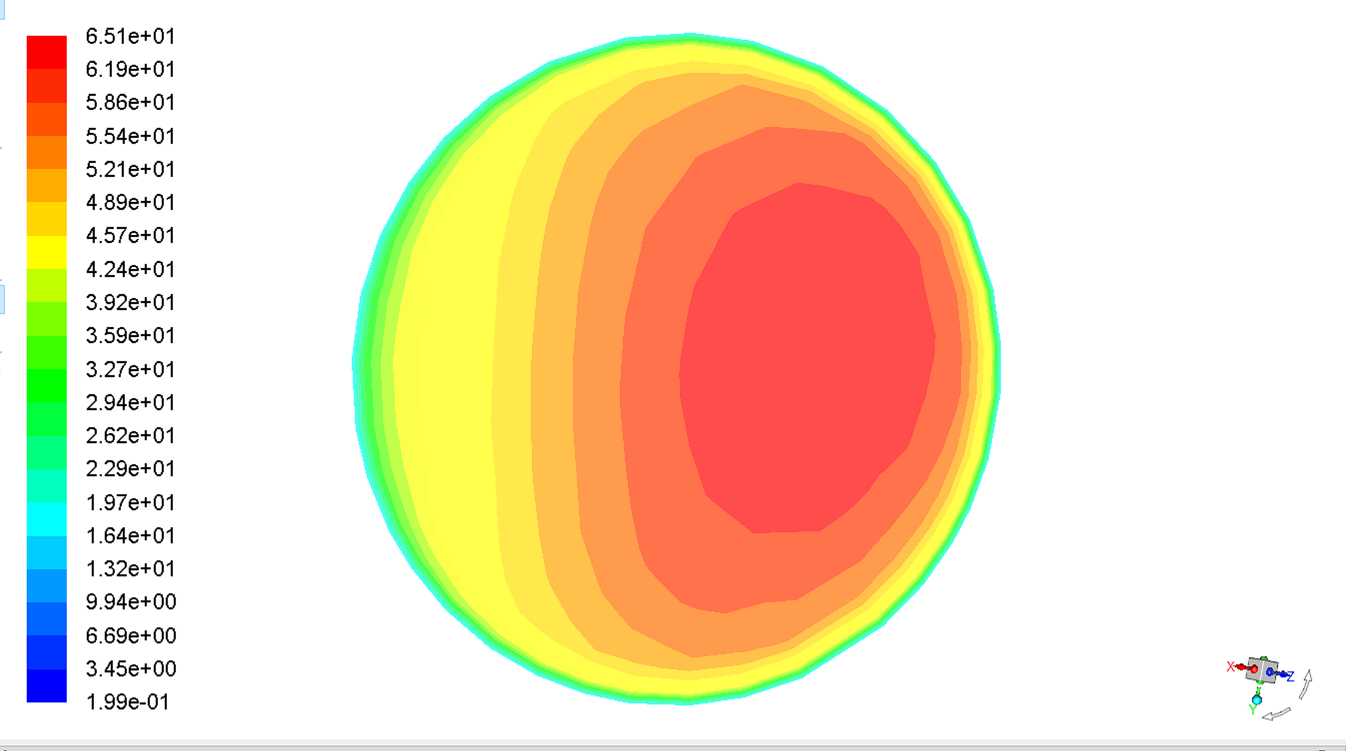  (4) | 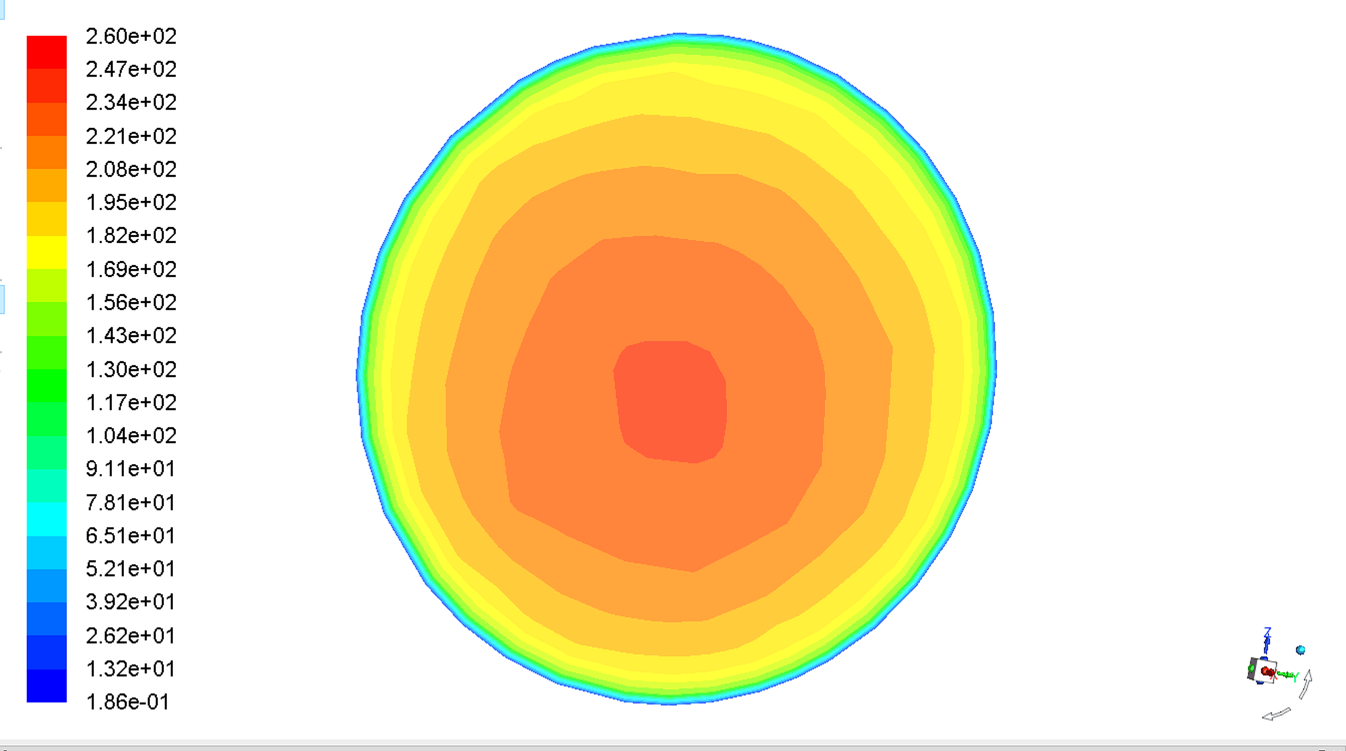  (5) | 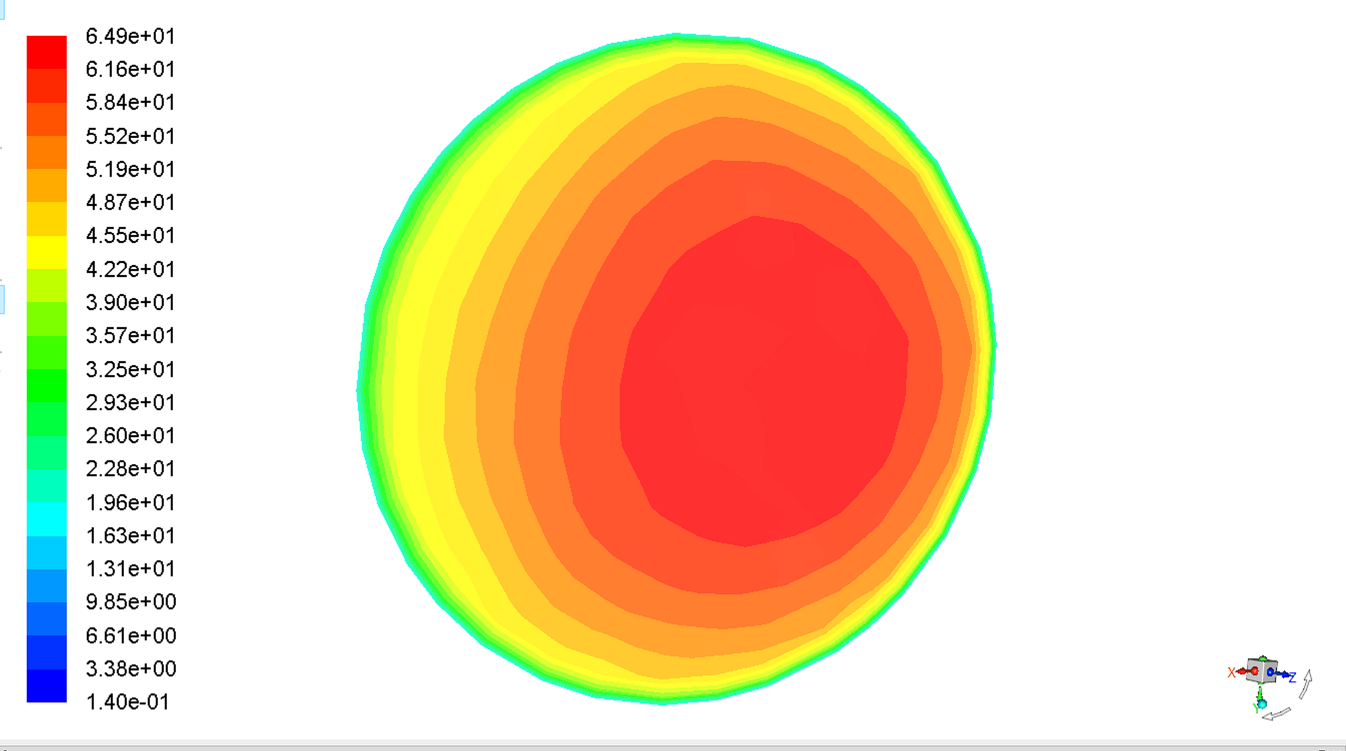  (6) |
| 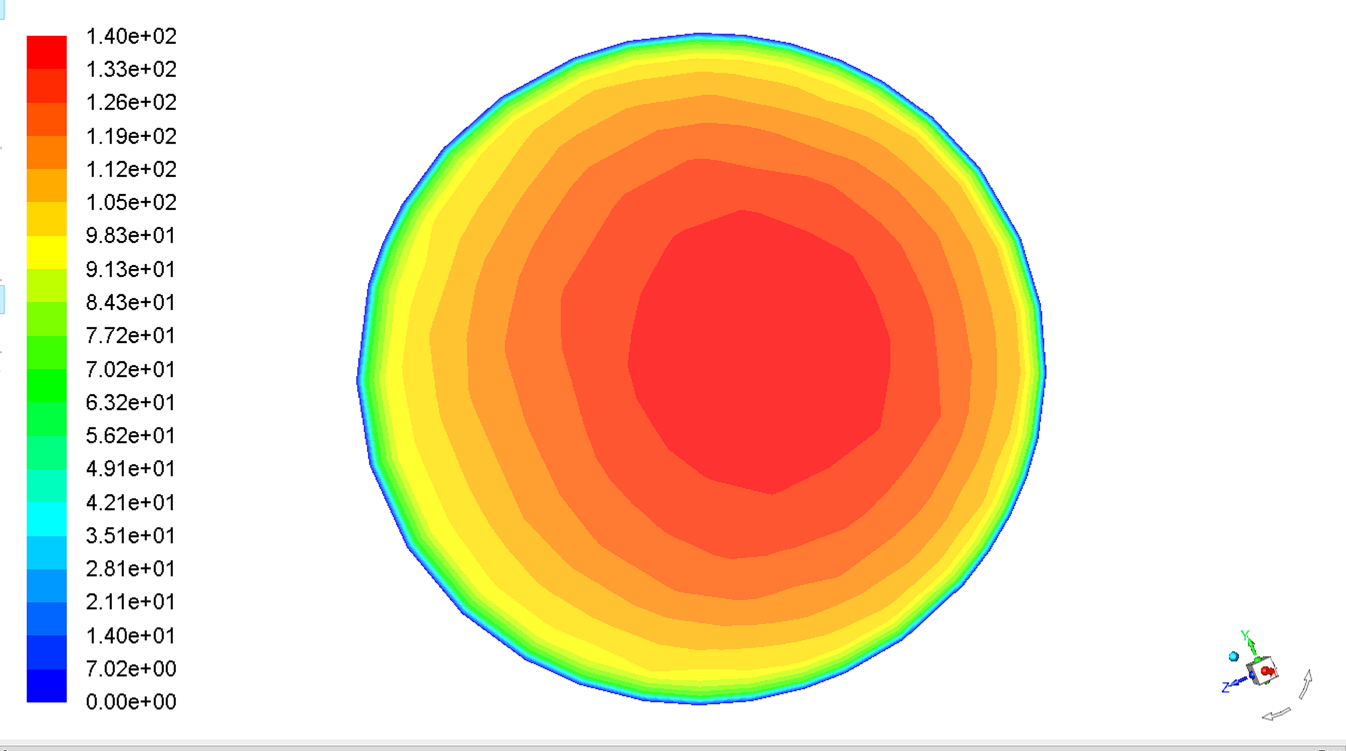  (7) | 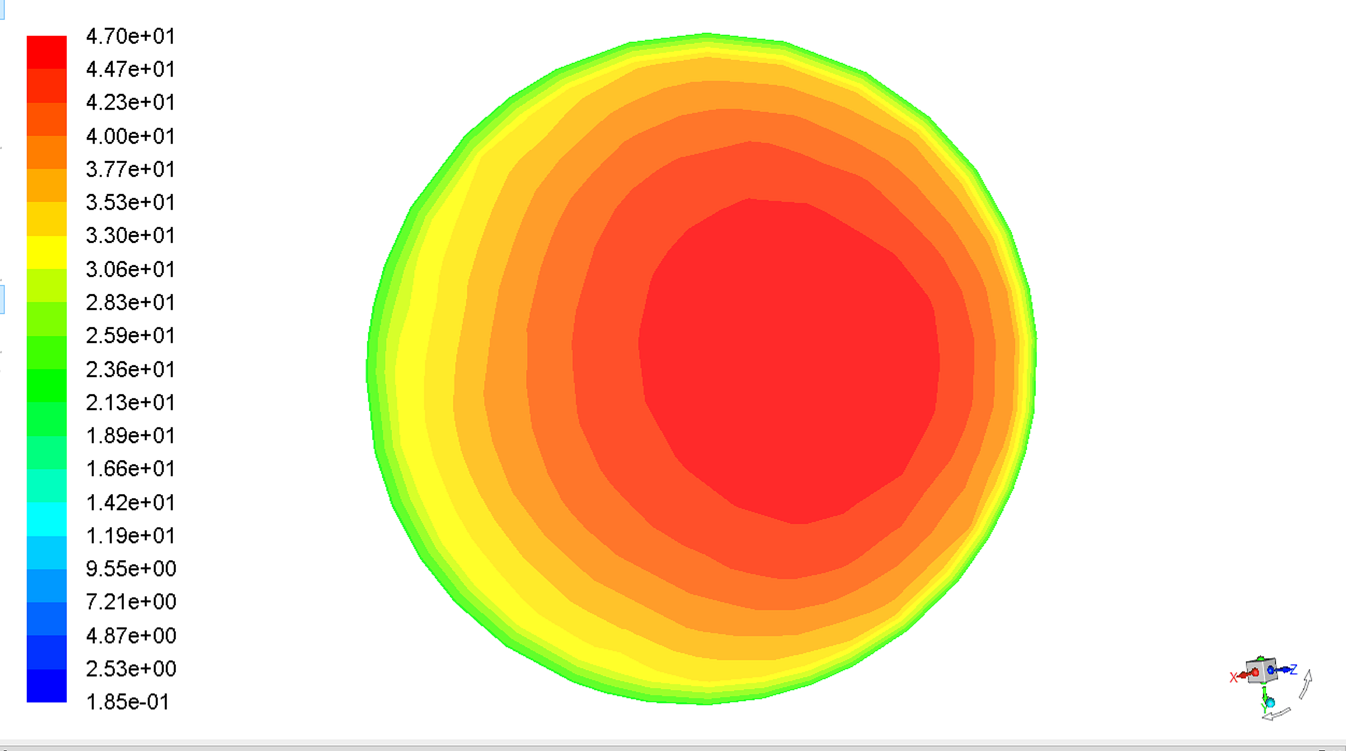  (8) | 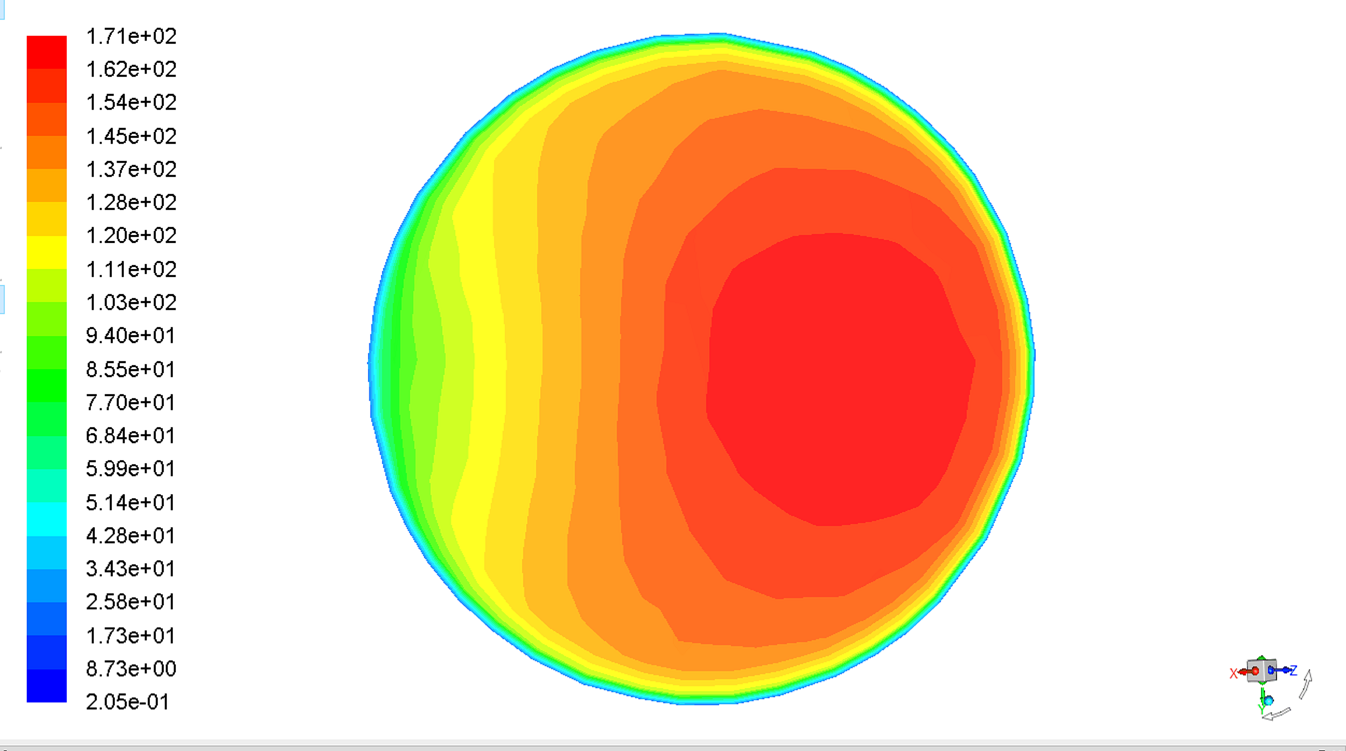  (9) |
| **Figure S7 |** Solution velocity distribution at the outlet section of stepped nozzles of 9 tests | | |

| **A**  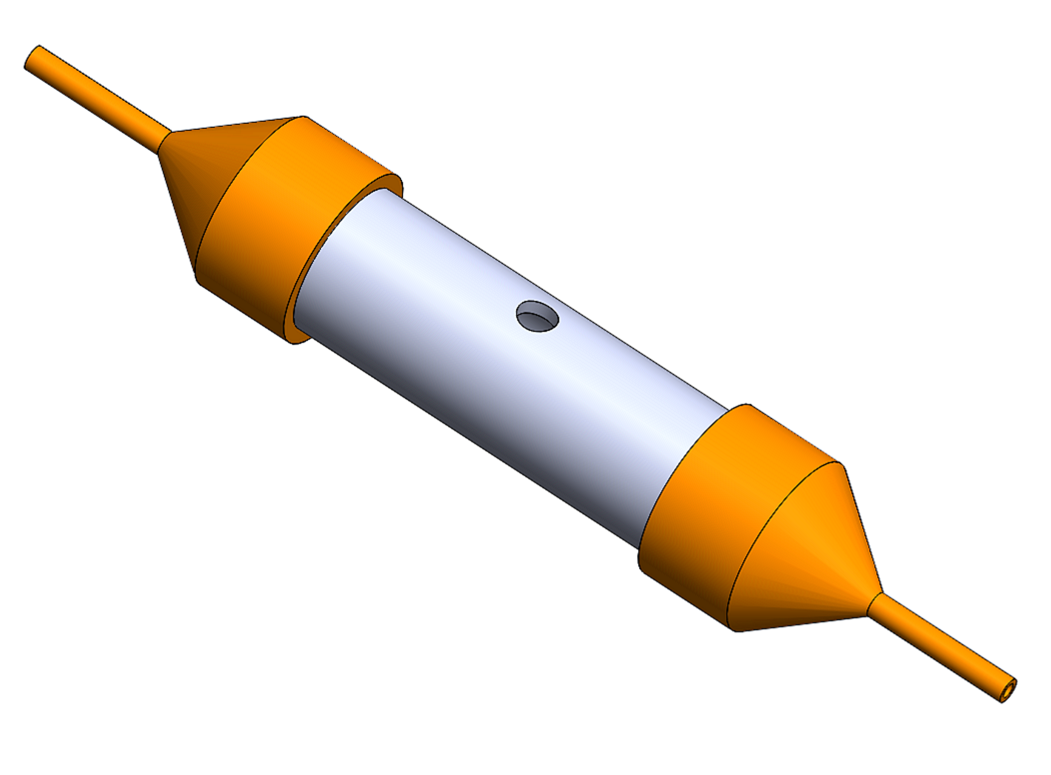 | **B**  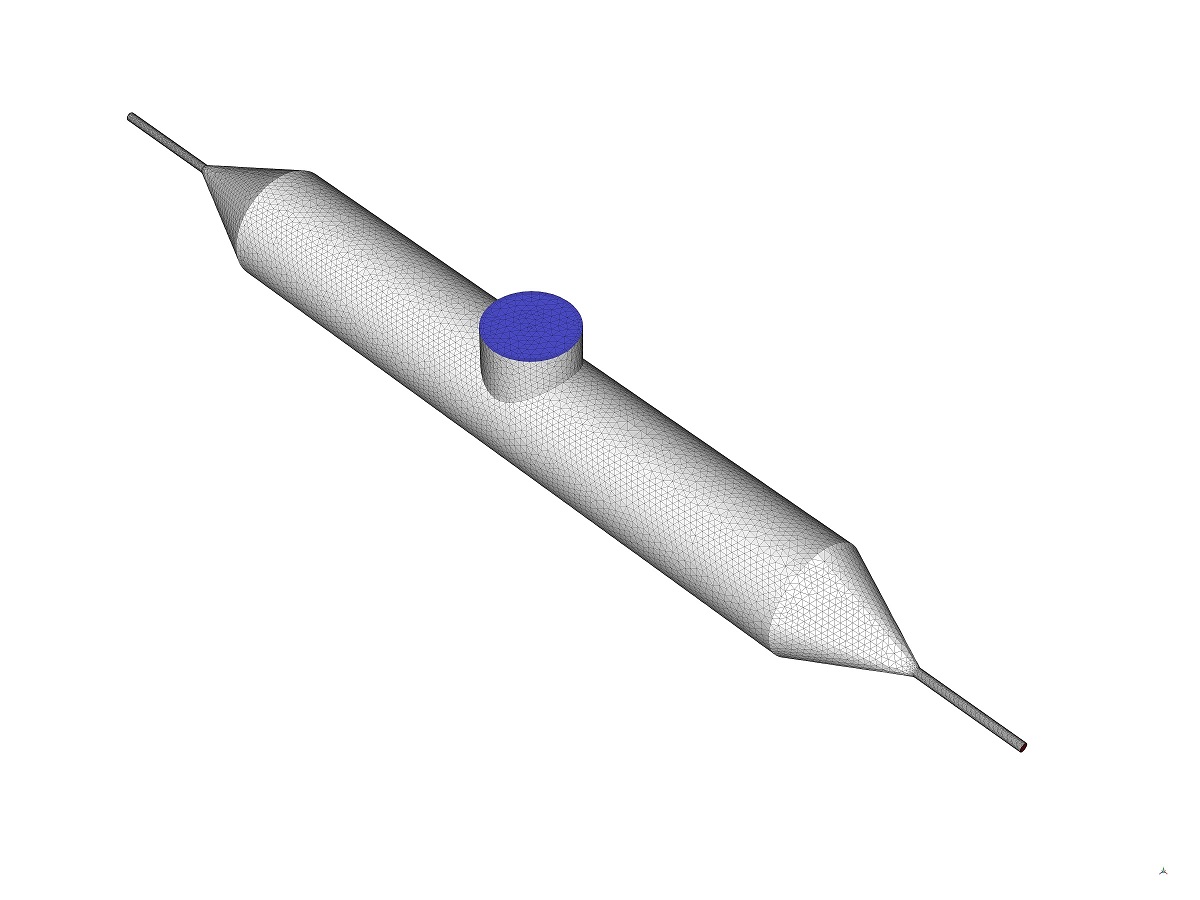 |
| --- | --- |
| **Figure S8 |** The model of the spinneret with conical-straight nozzles. **(A)** The spinneret with conical-straight nozzles; **(B)** The mesh division of spinneret with conical-straight nozzles | |

| 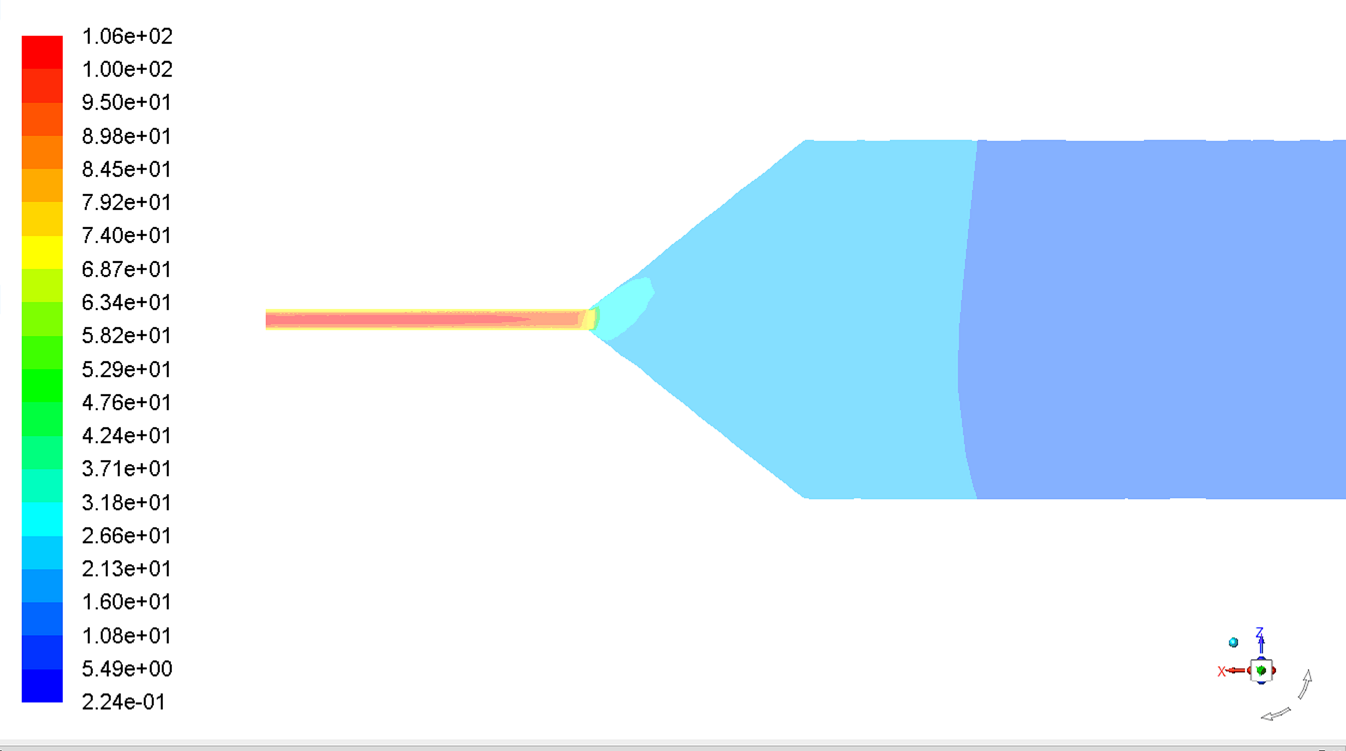  (1) | 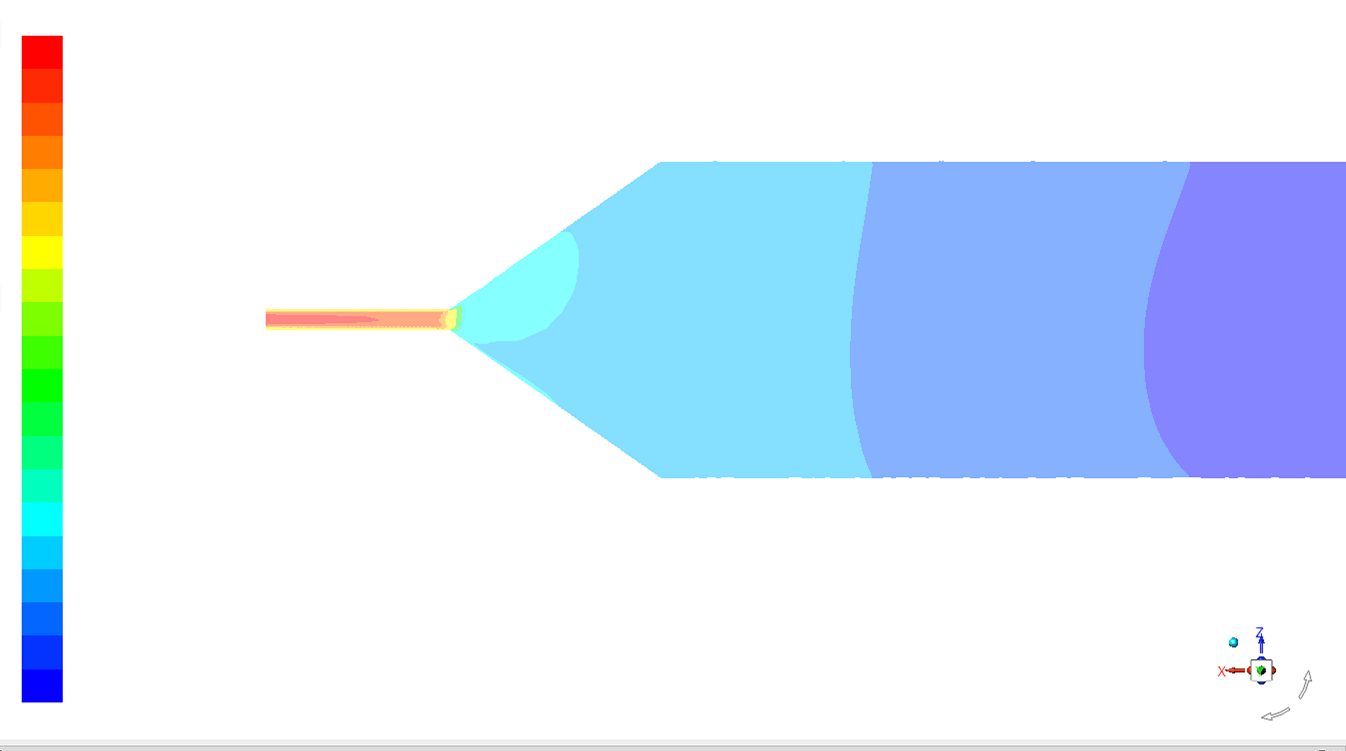  (2) | 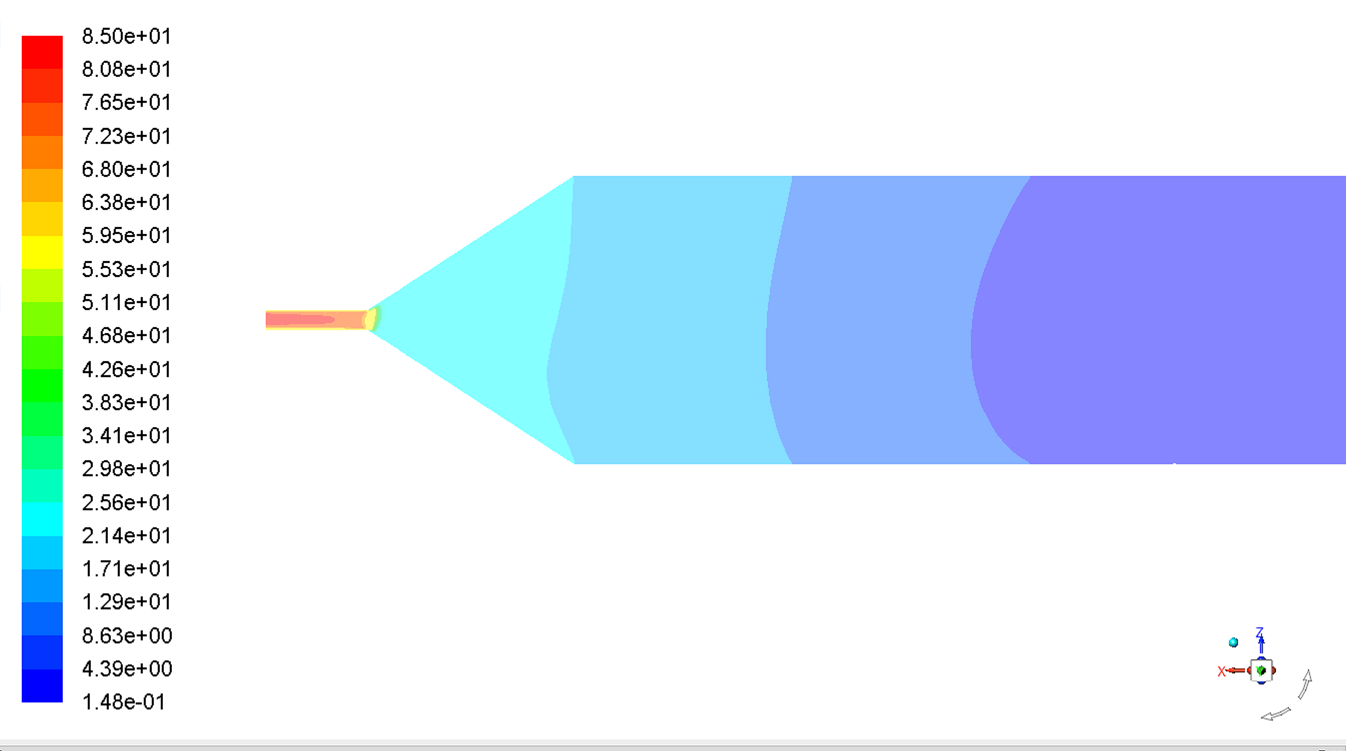  (3) |
| --- | --- | --- |
| 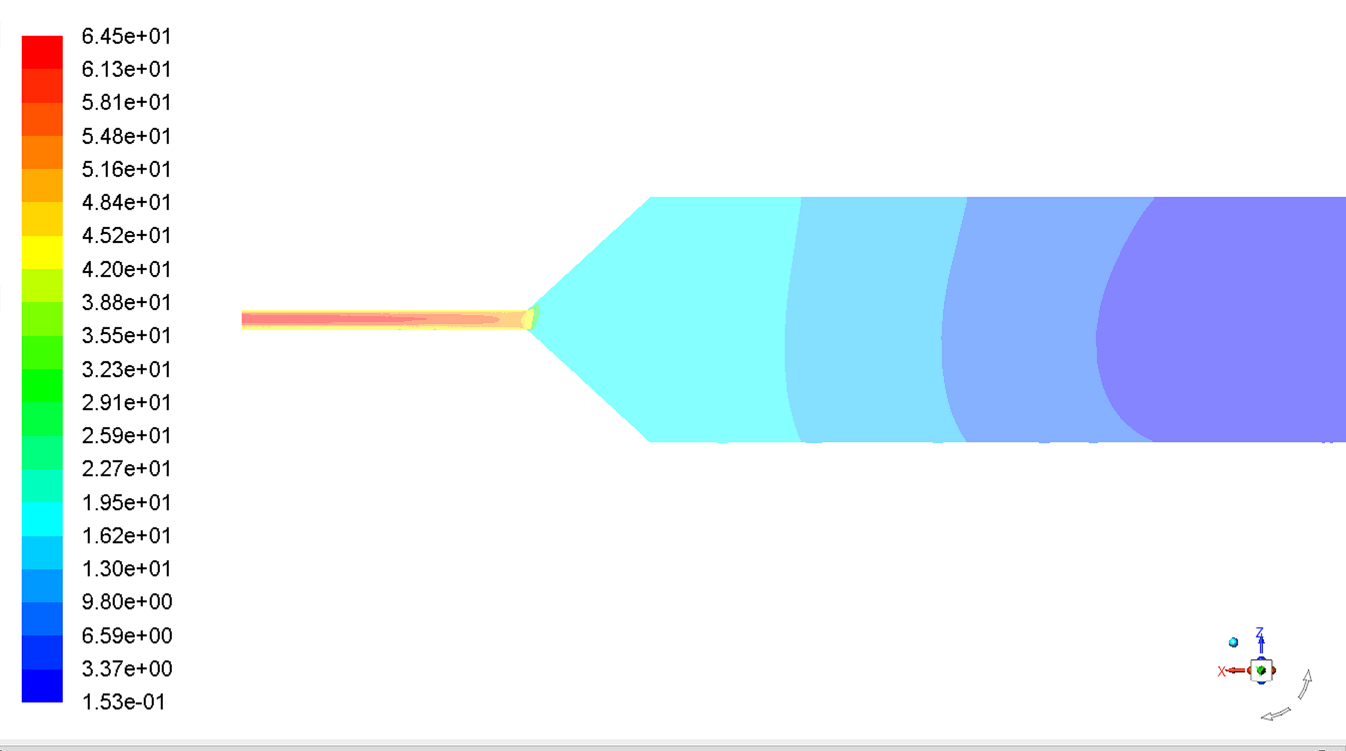  (4) | 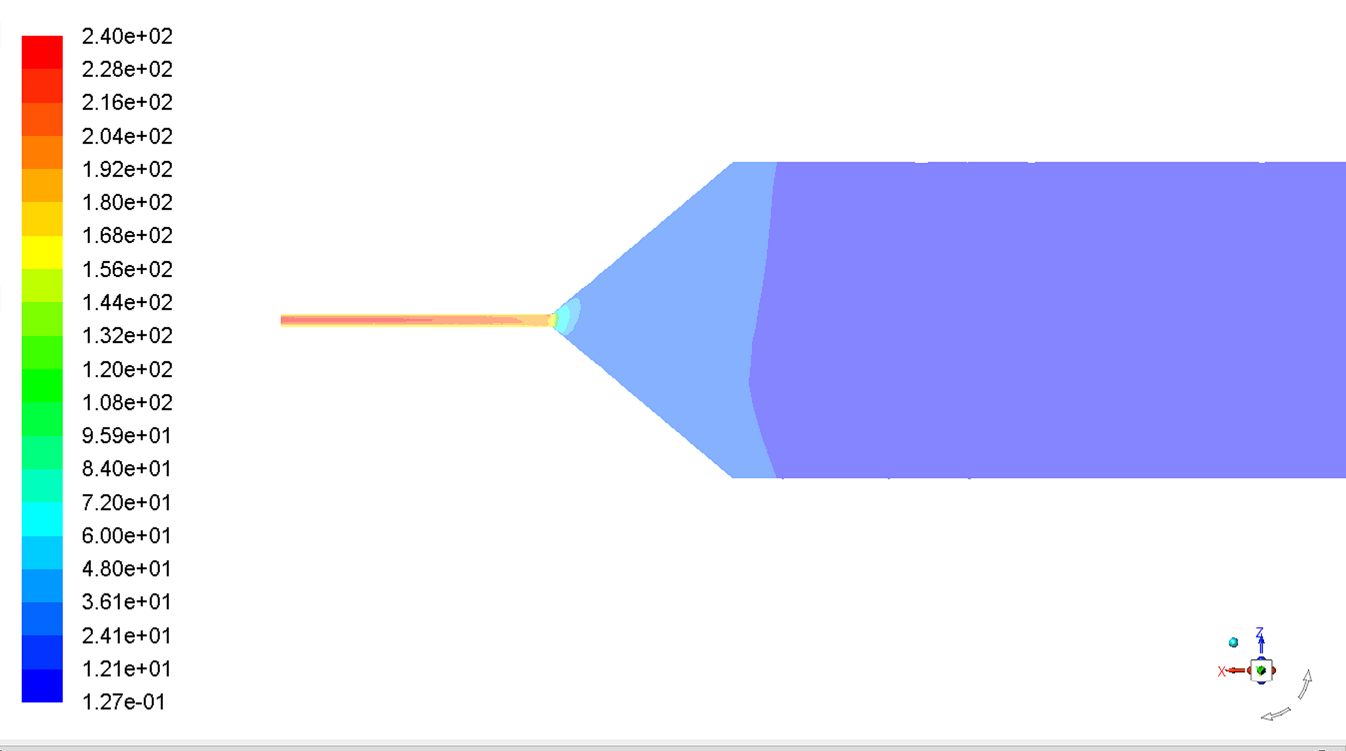  (5) | 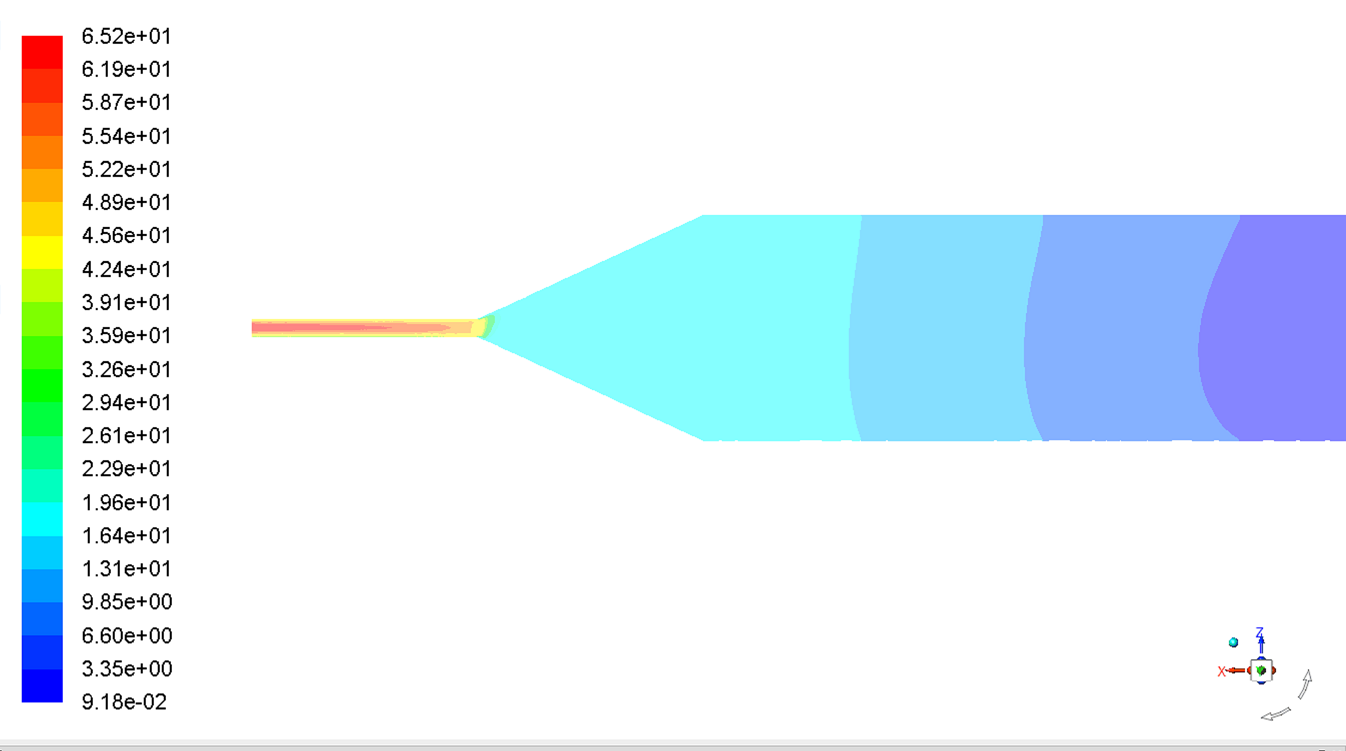  (6) |
| 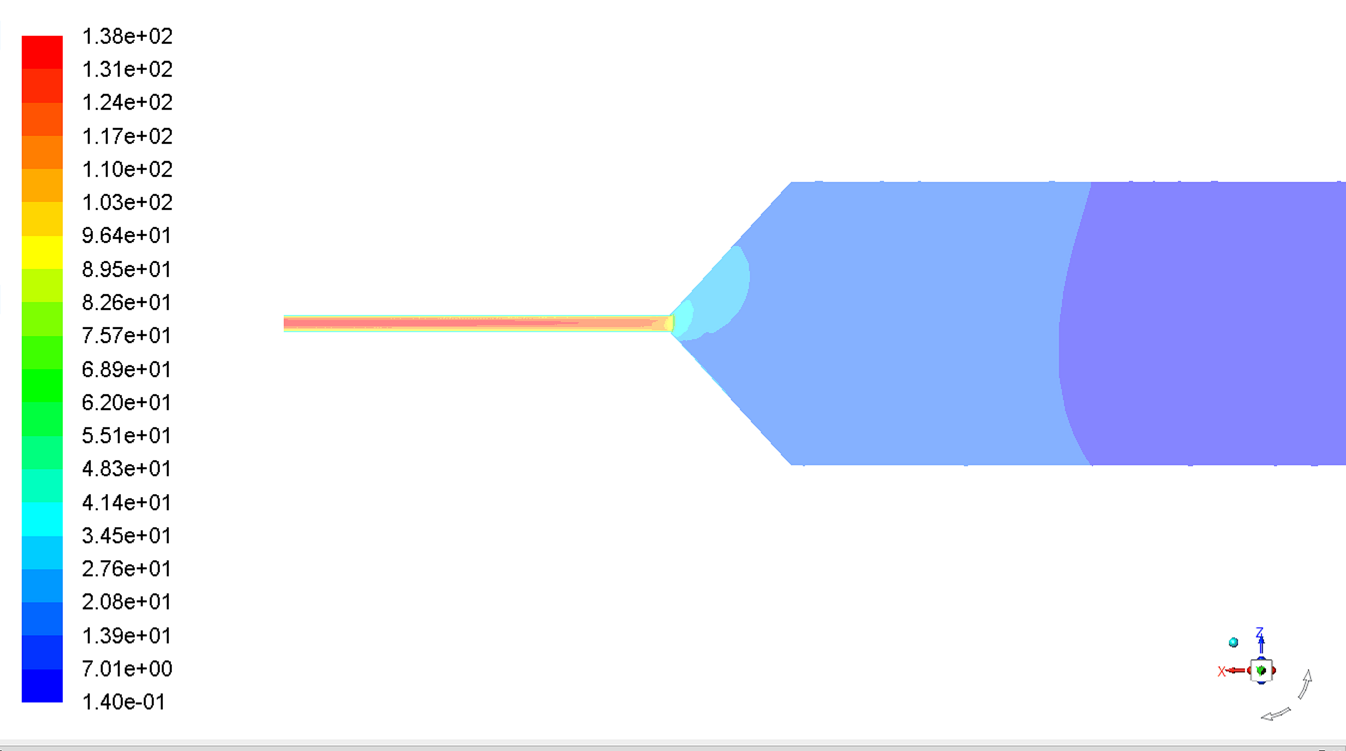  (7) | 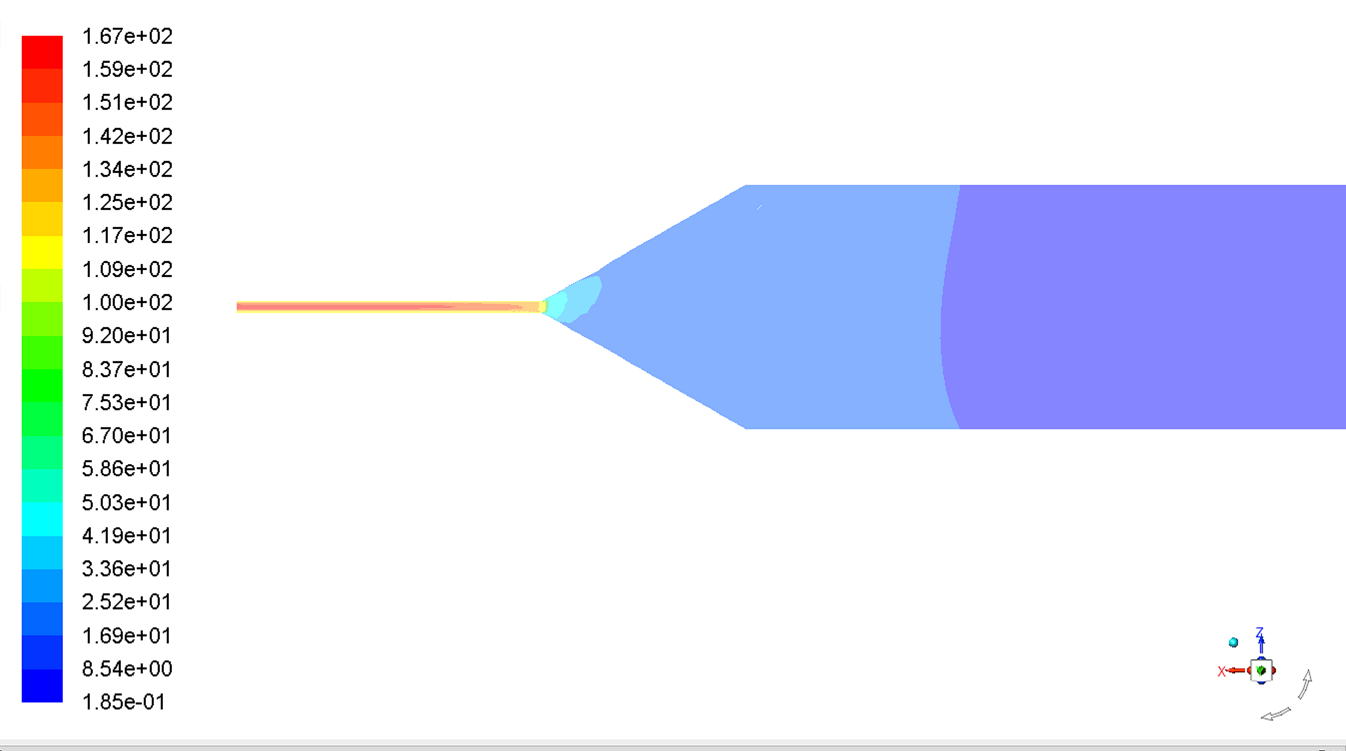  (8) | 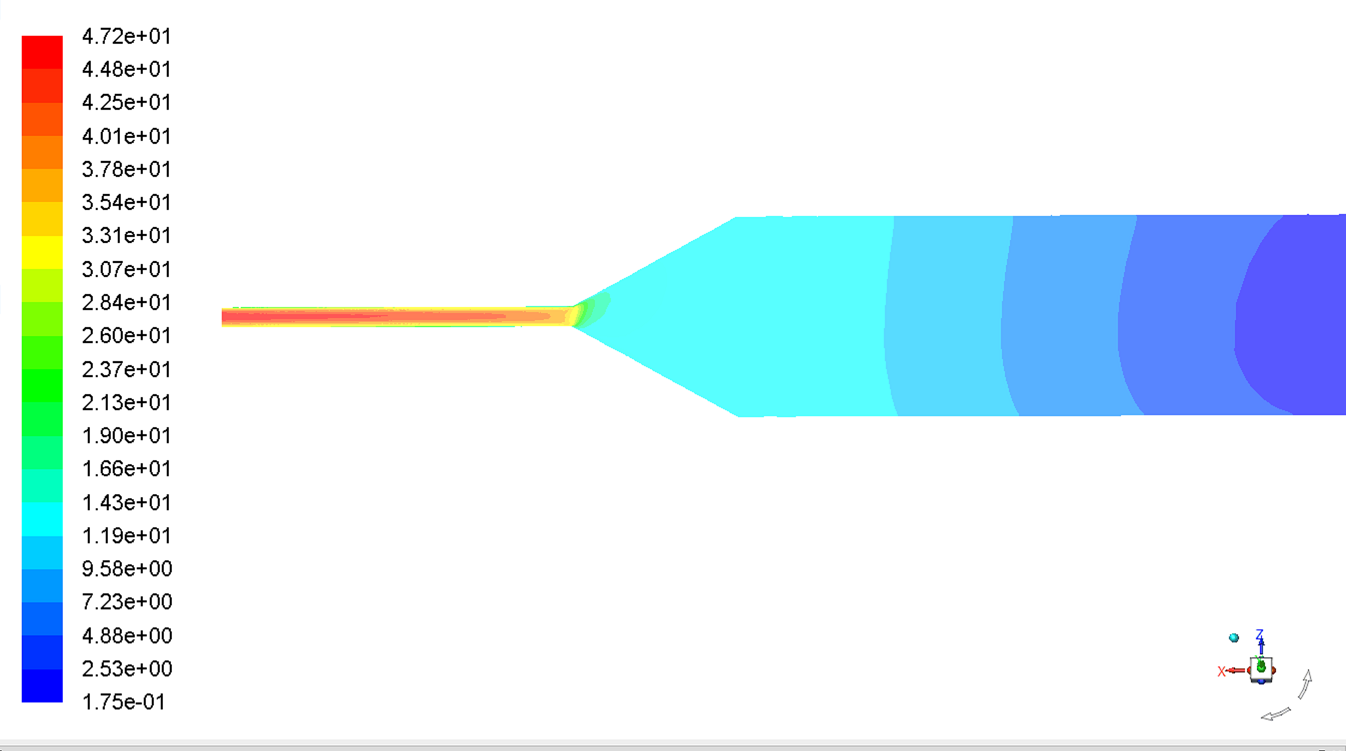  (9) |
| **Figure S9 |** Cloud diagram of solution velocity distribution in conical-straight nozzles of 9 tests | | |

| 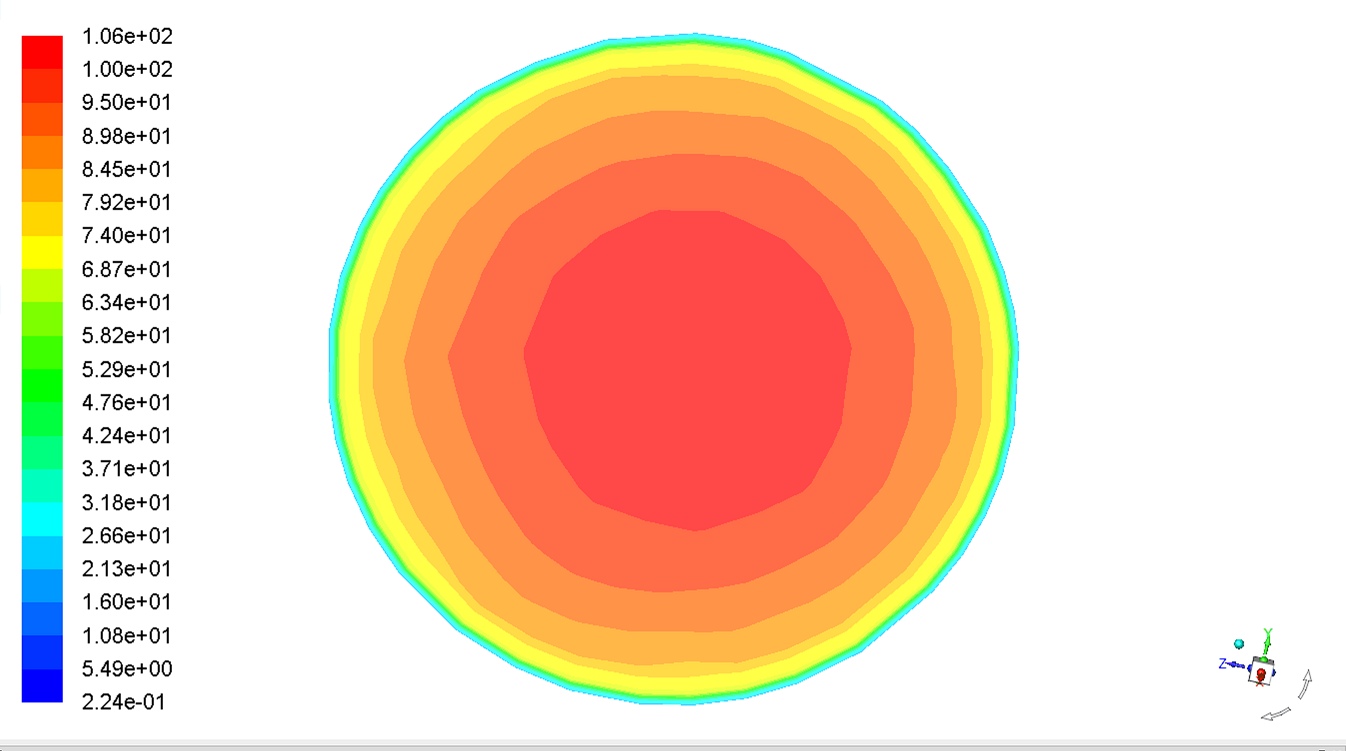  (1) | 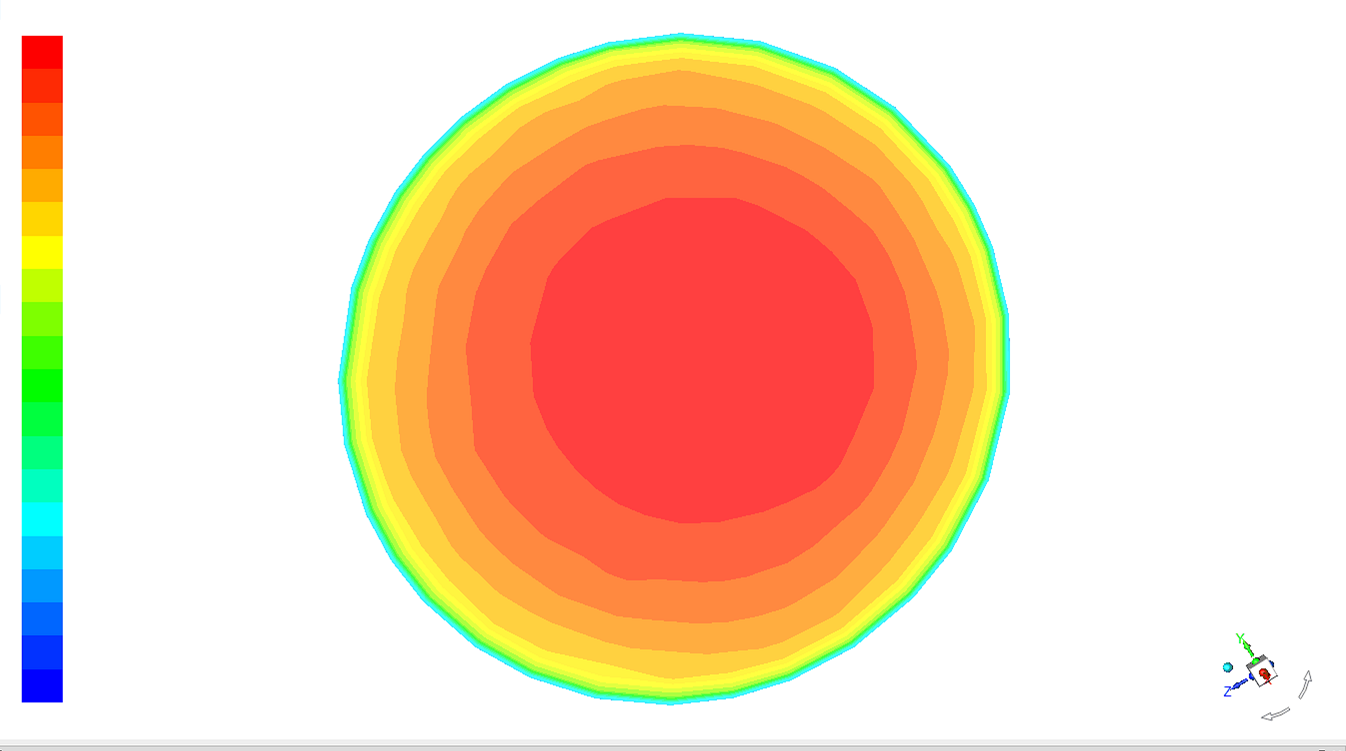  (2) | 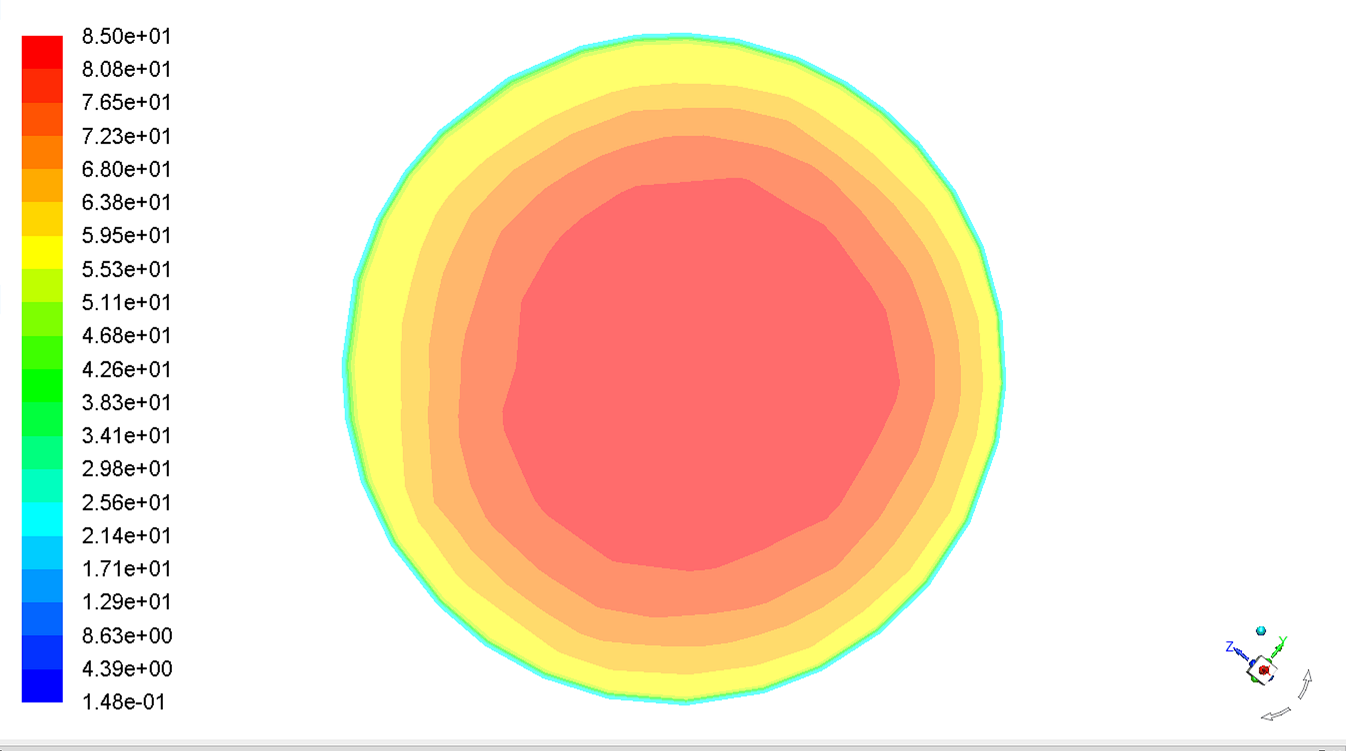  (3) |
| --- | --- | --- |
| 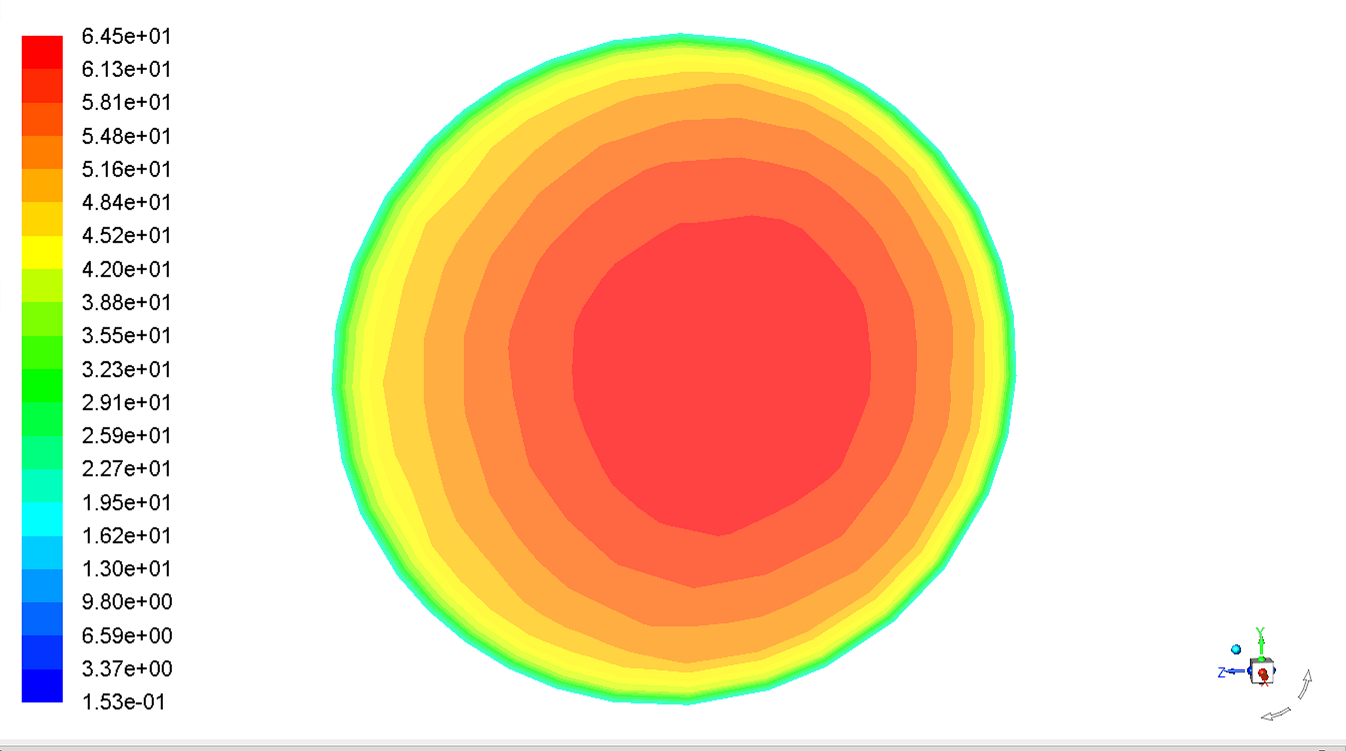  (4) | 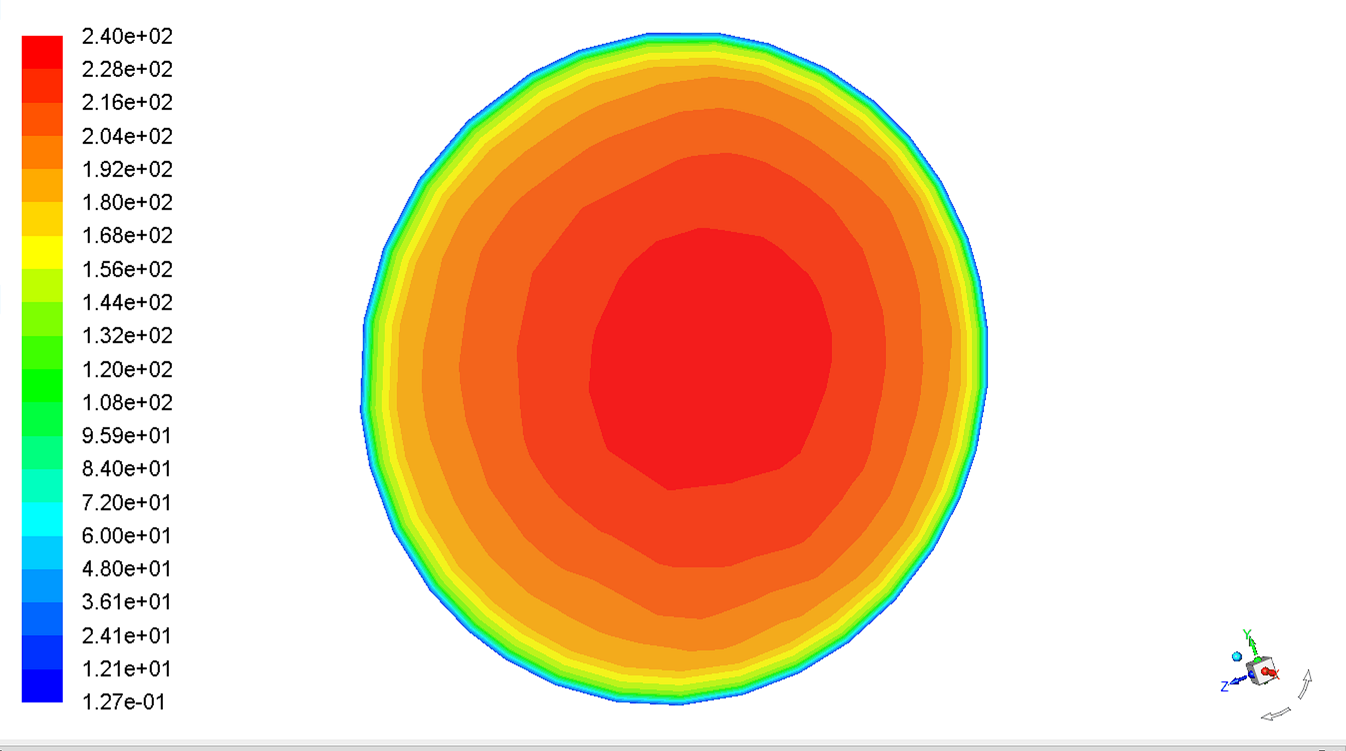  (5) | 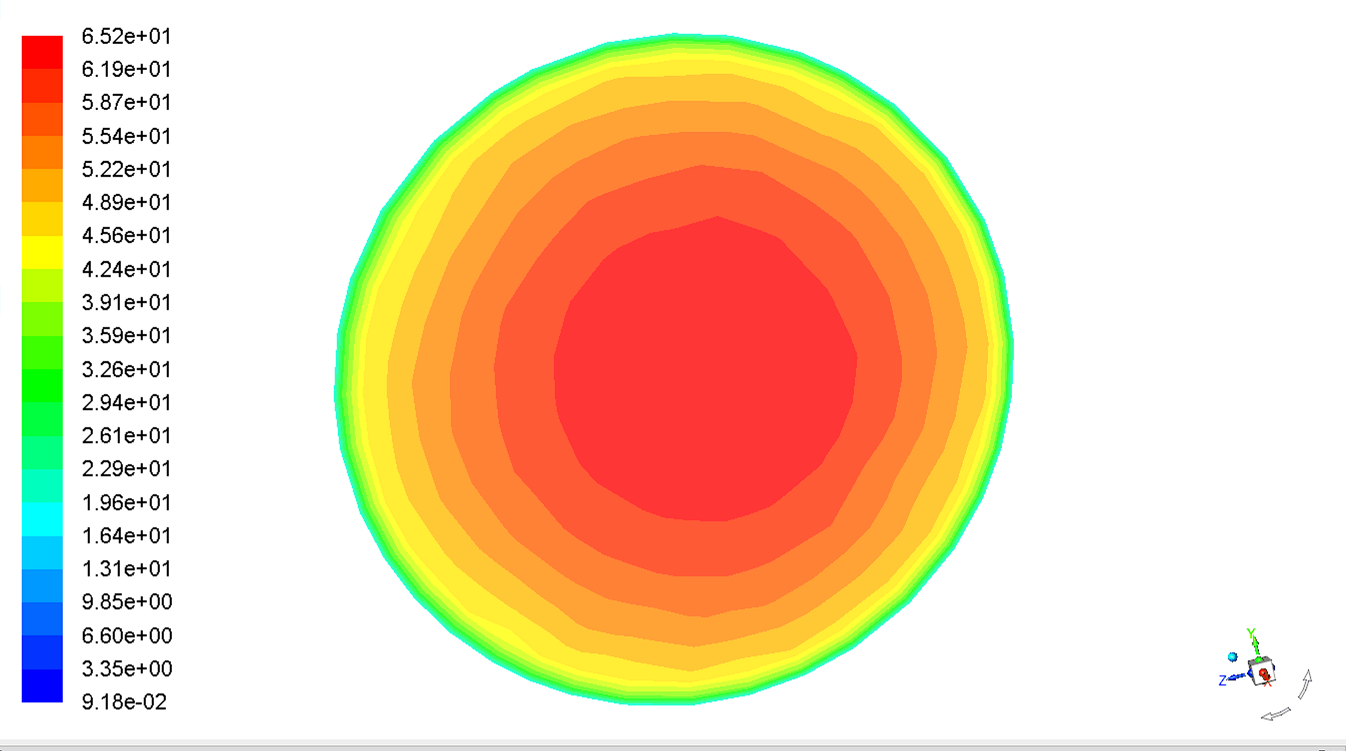  (6) |
| 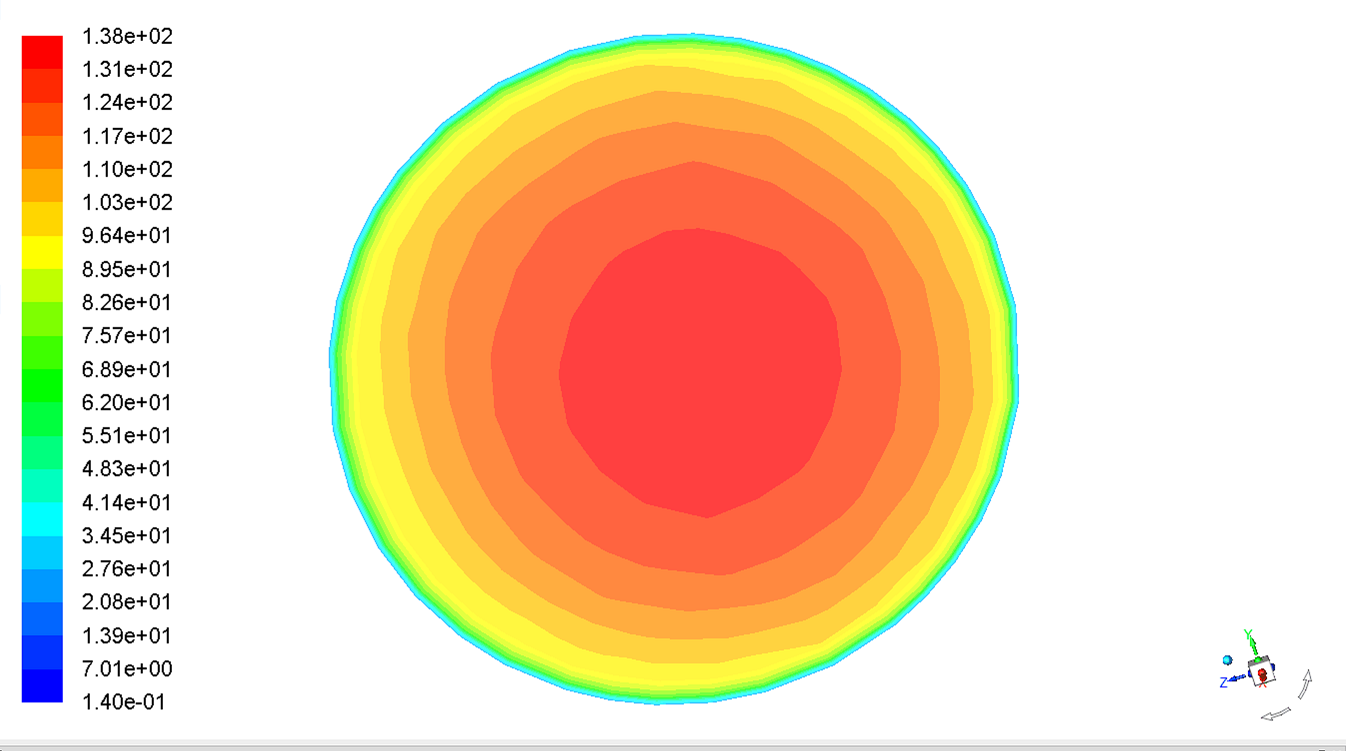  (7) | 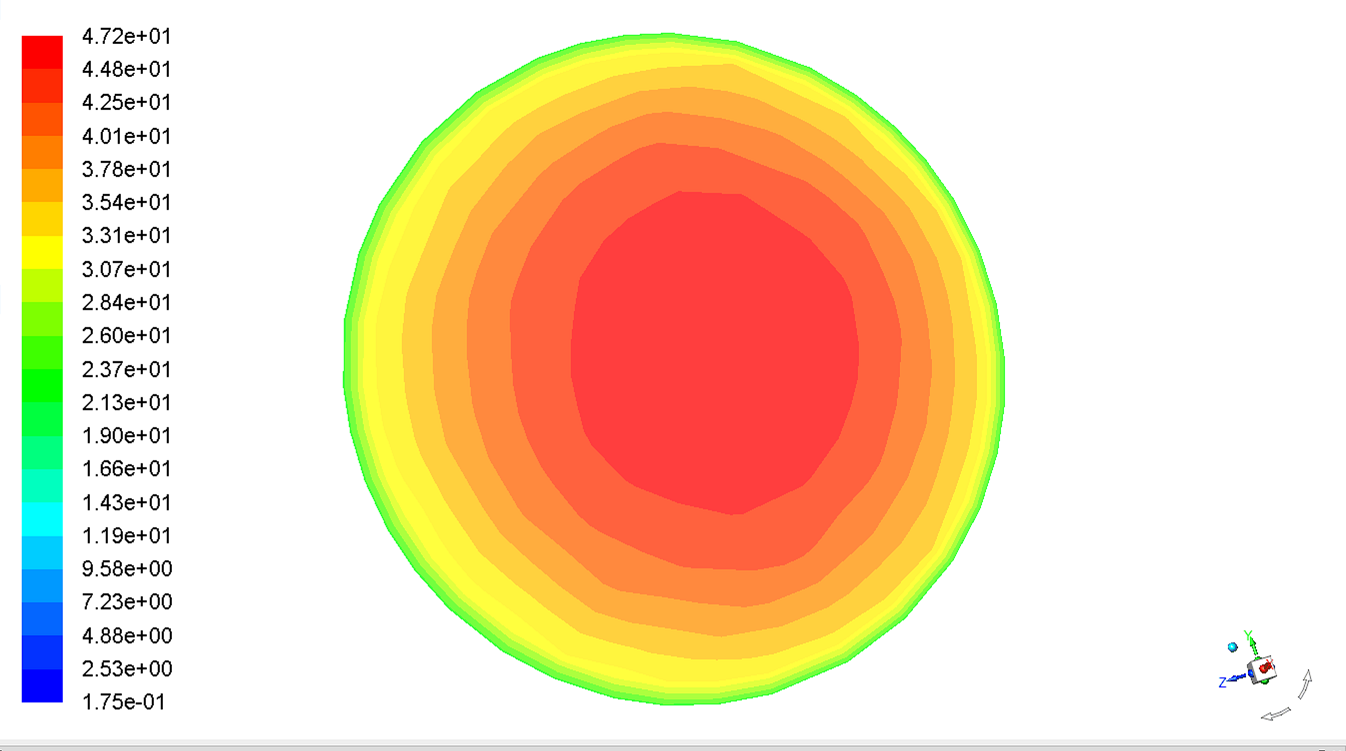  (8) | 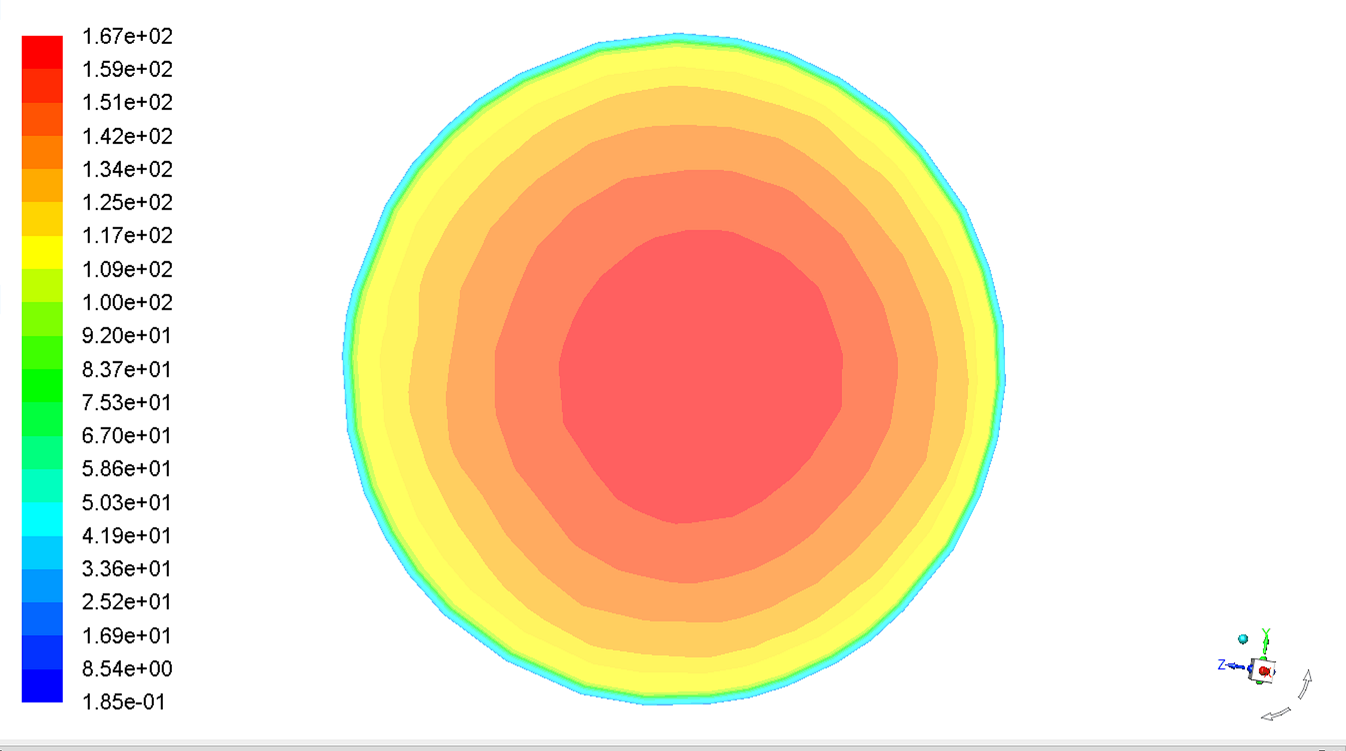  (9) |
| **Figure S10 |** Solution velocity distribution at the outlet section of conical-straight nozzles of 9 tests | | |

| **A**  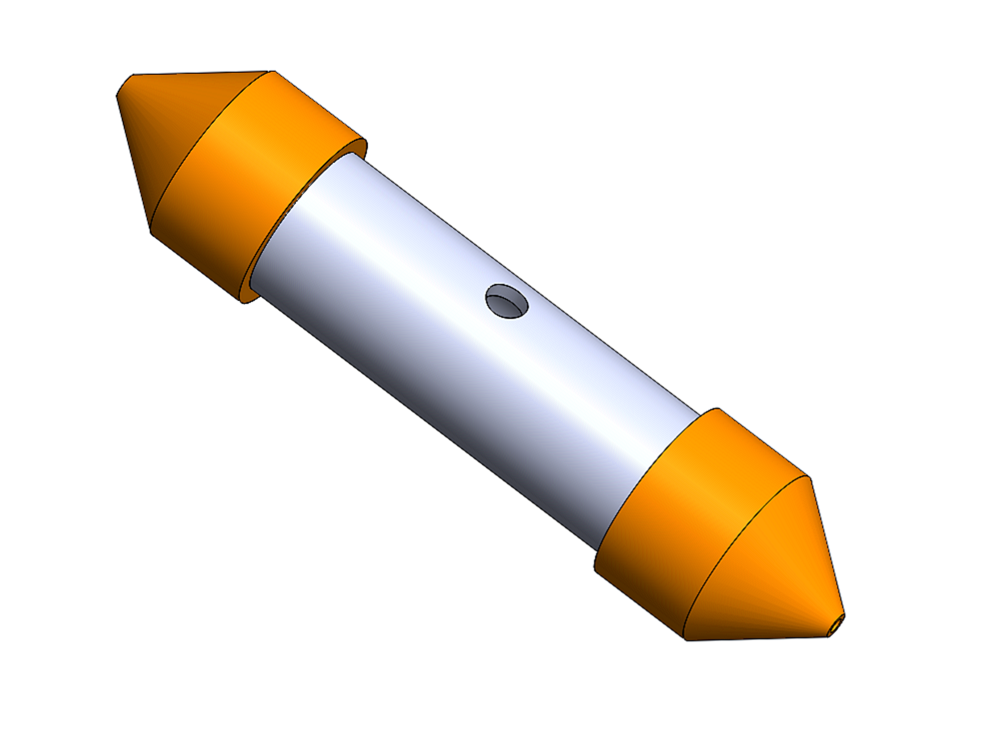 | **B**  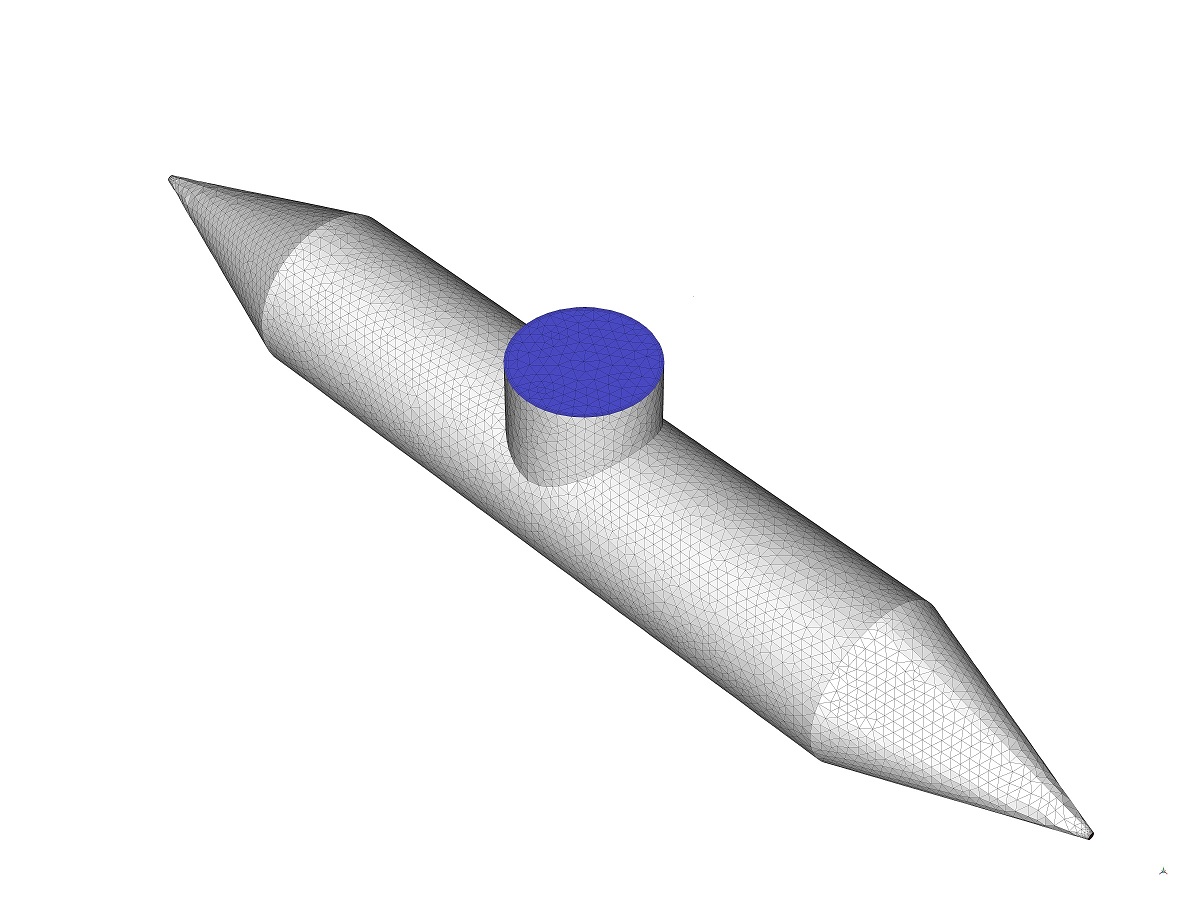 |
| --- | --- |
| **Figure S11 |** The model of the spinneret with conical nozzles. **(A)** The spinneret with conical nozzles; **(B)** The mesh division of spinneret with conical nozzles | |

| 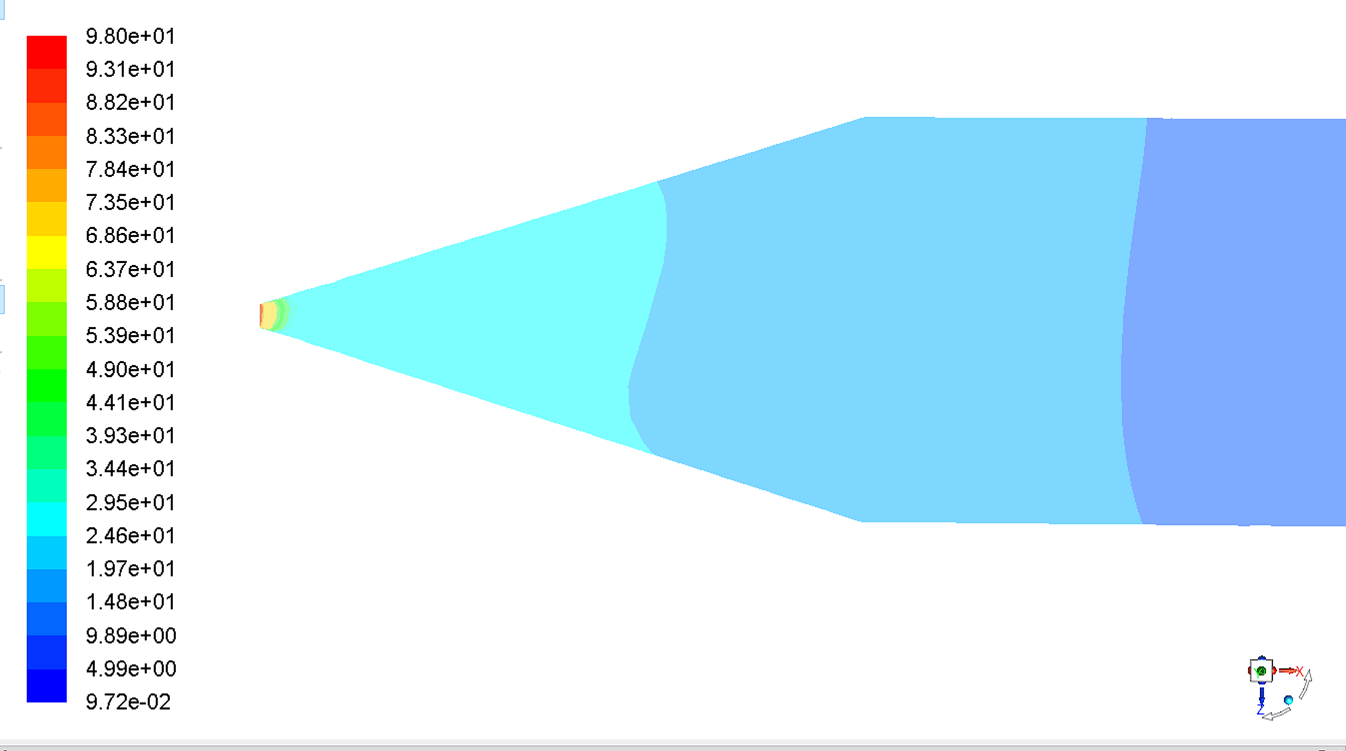  (1) | 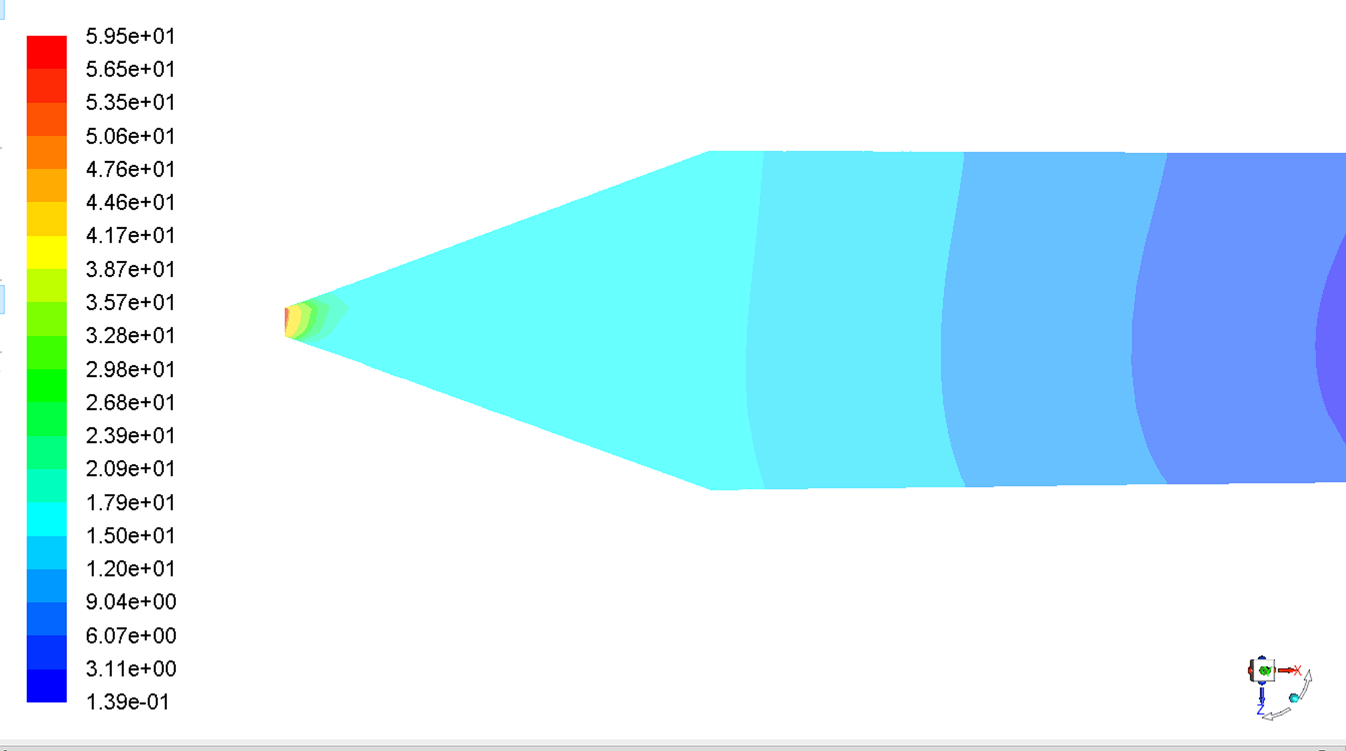  (2) | 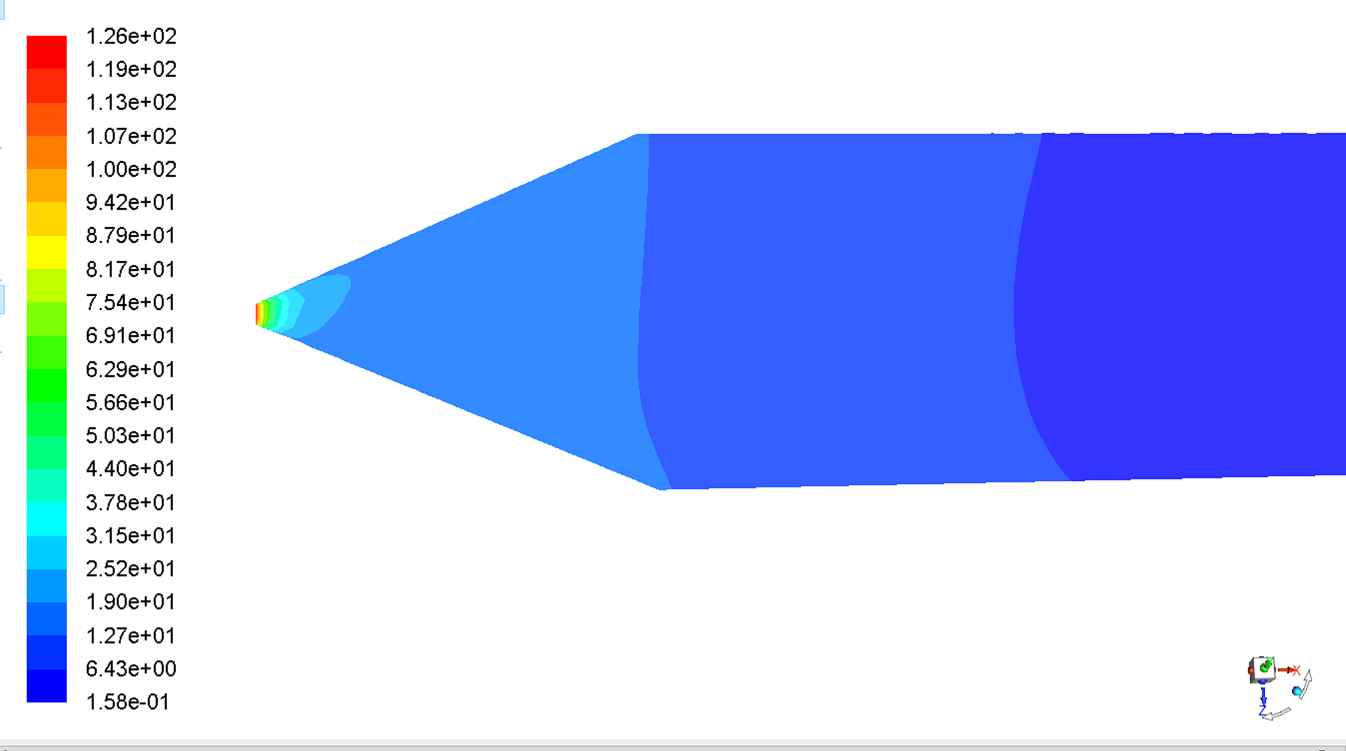  (3) |
| --- | --- | --- |
| 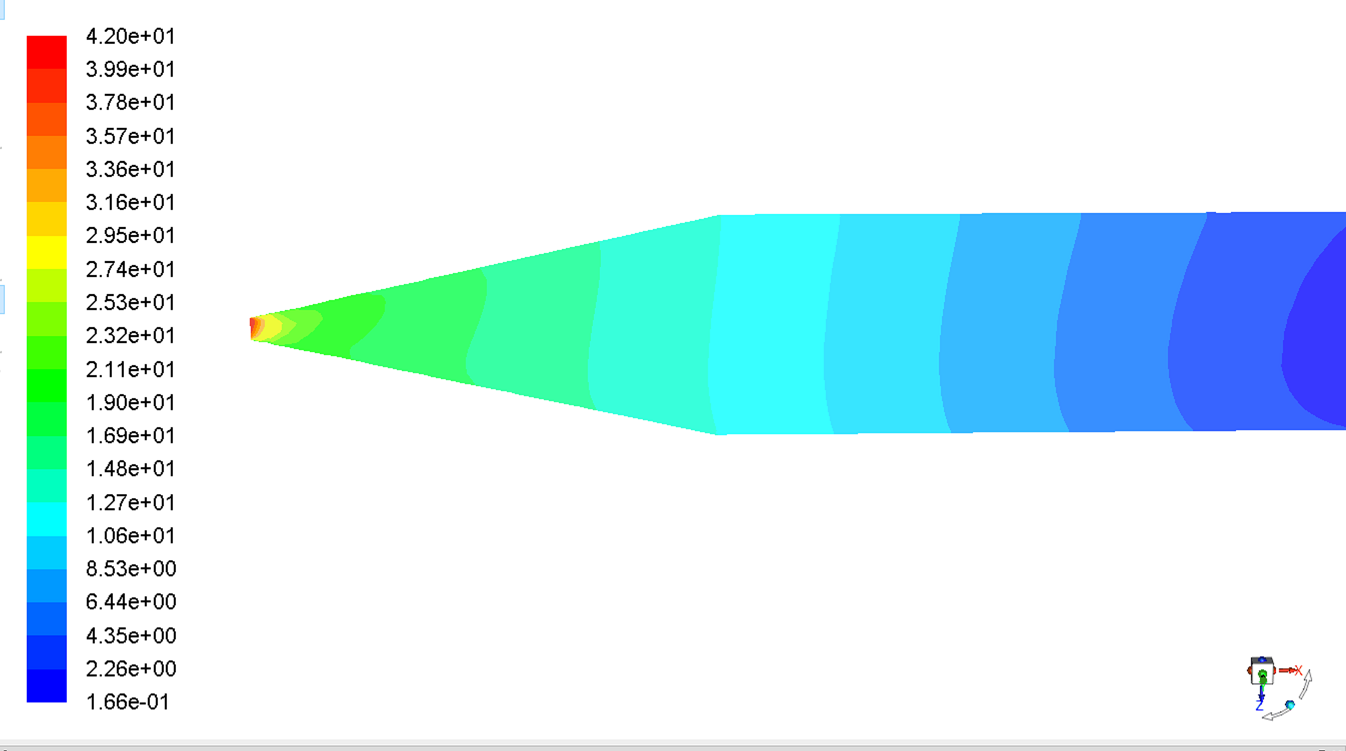  (4) | 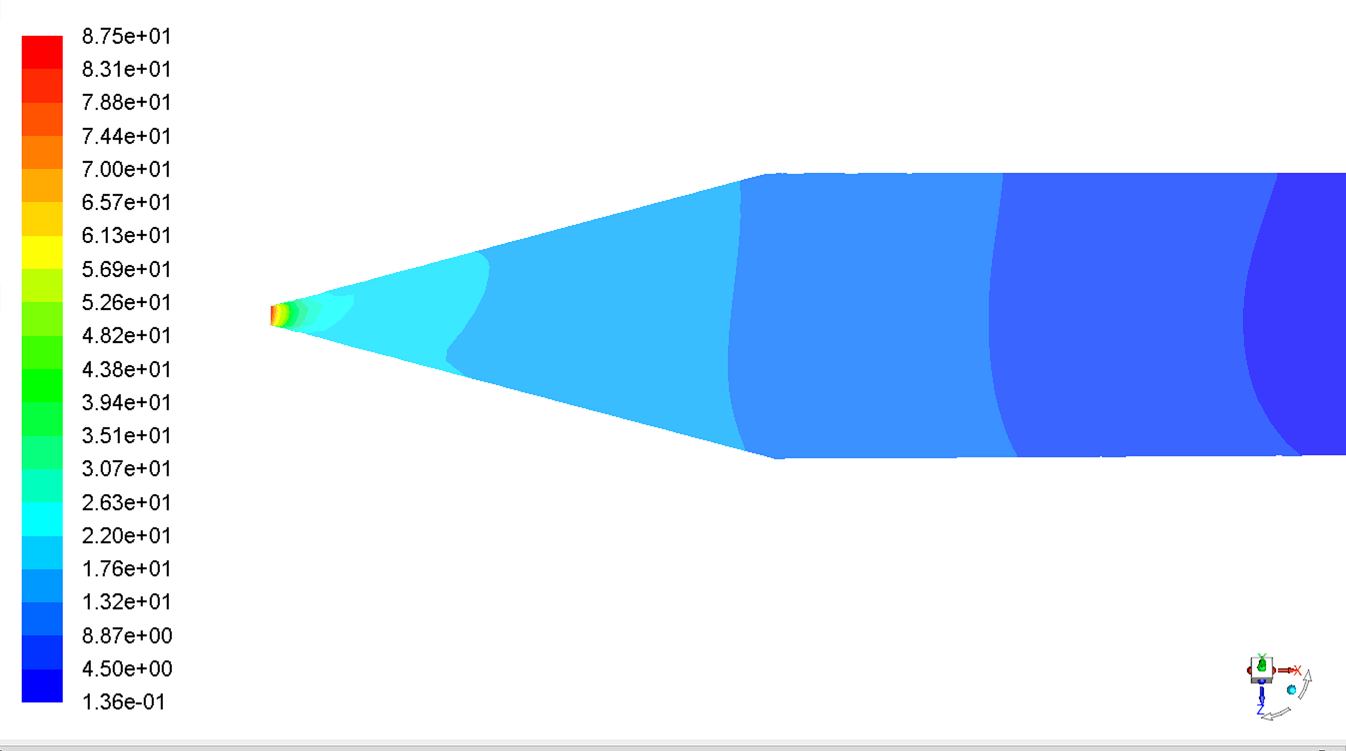  (5) | 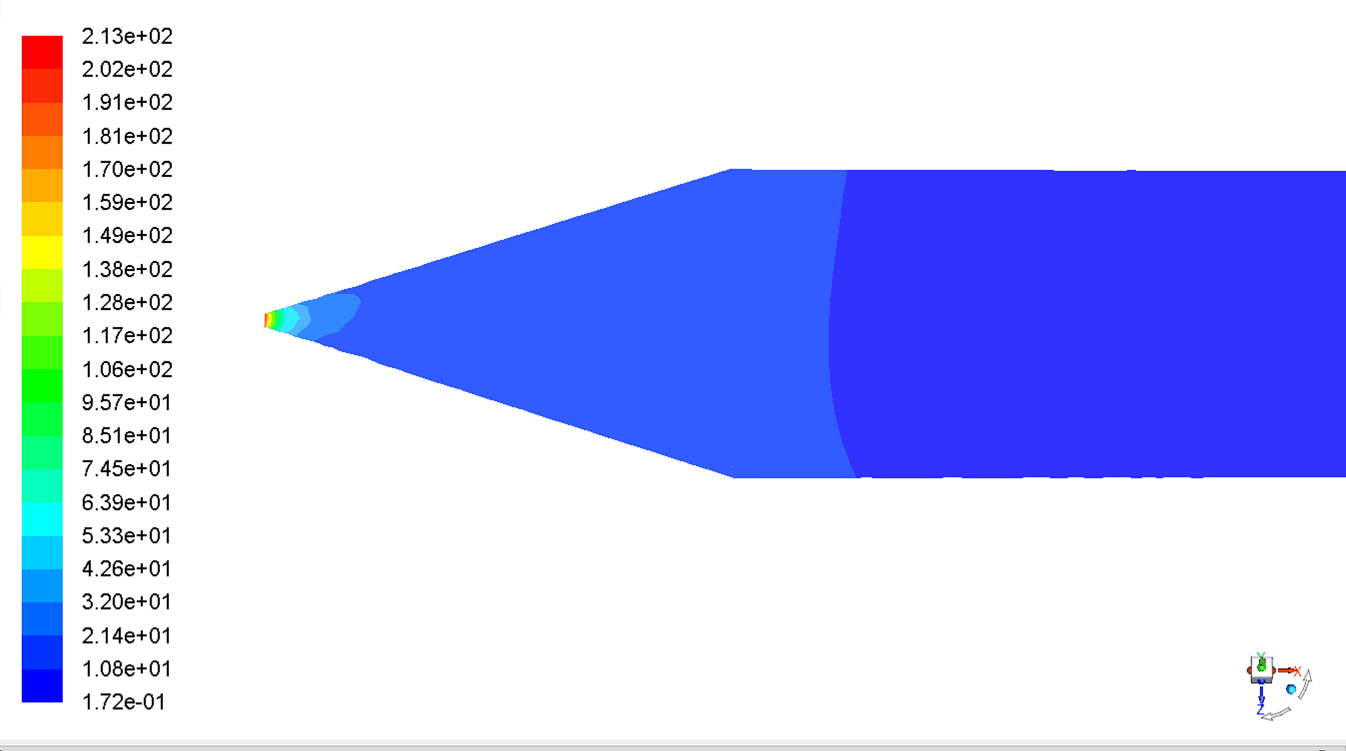  (6) |
| 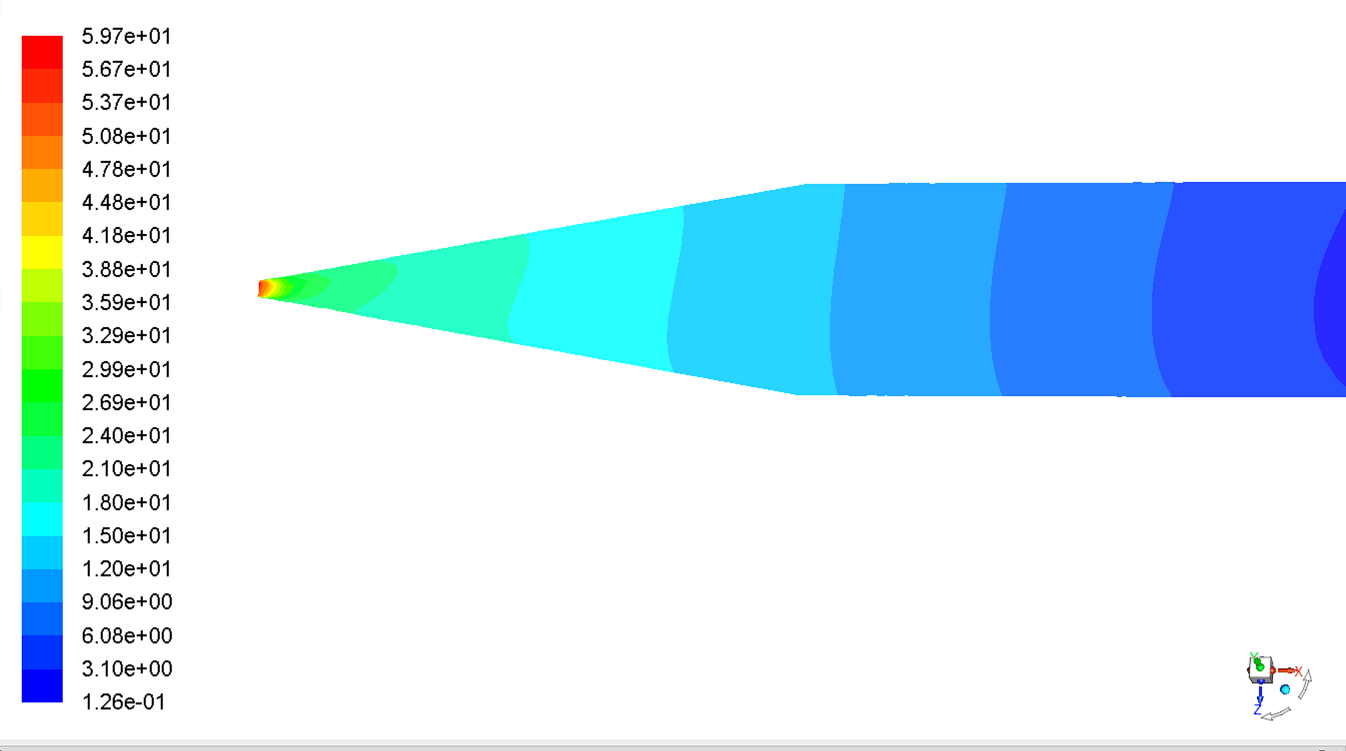  (7) | 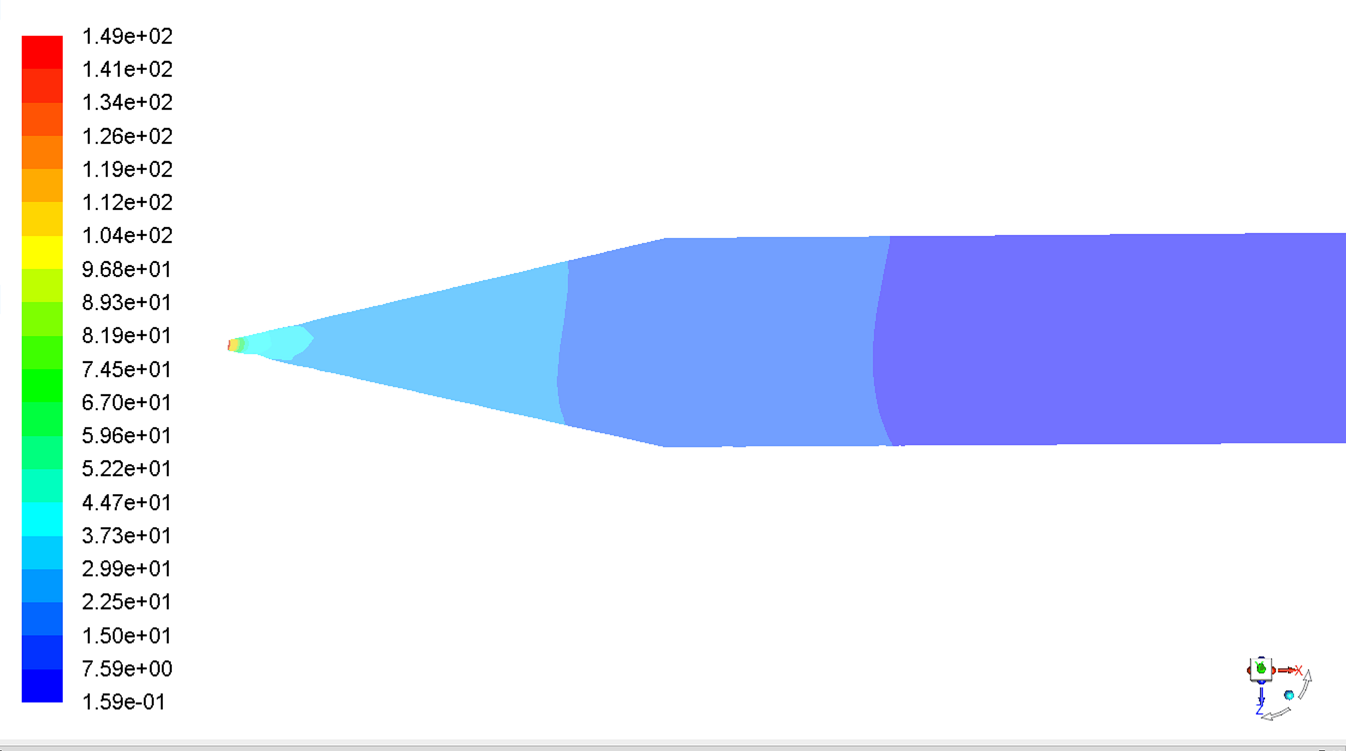  (8) | 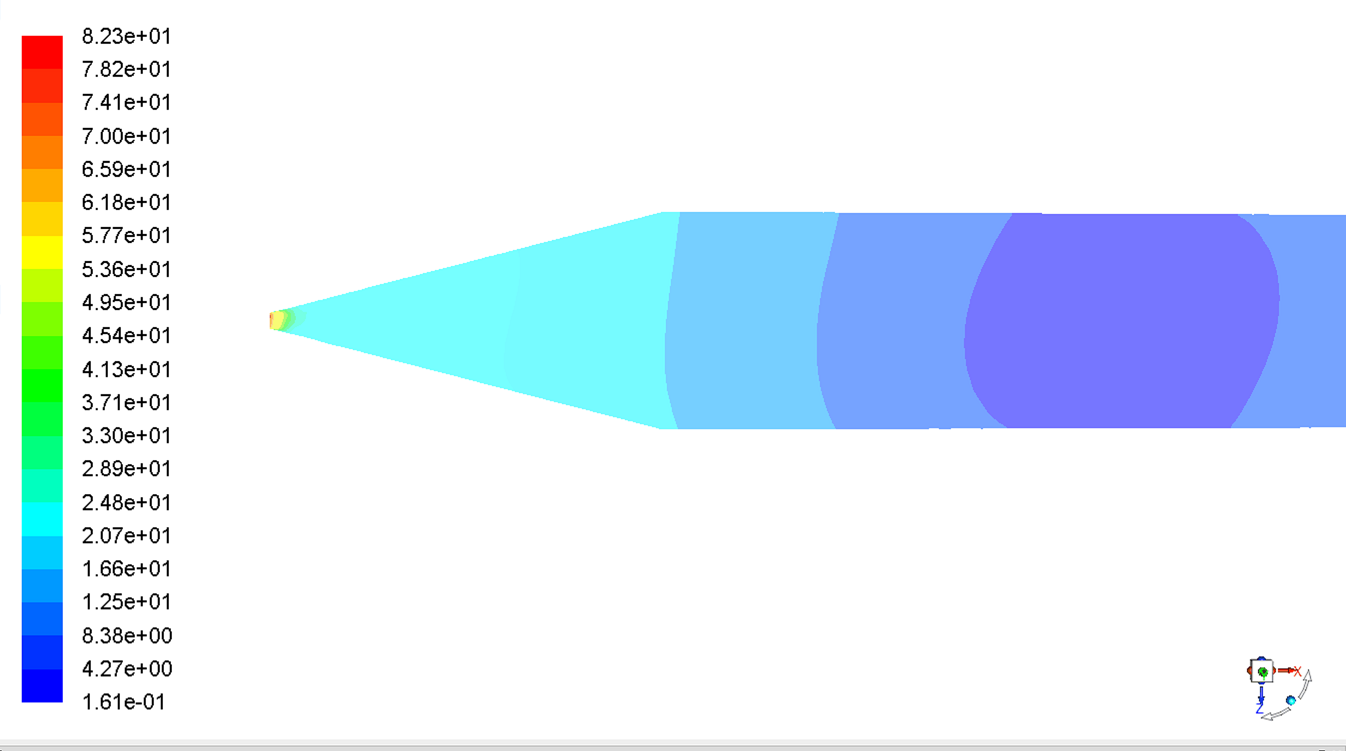  (9) |
| **Figure S12 |** Cloud diagram of solution velocity distribution in conical nozzles of 9 tests | | |

| 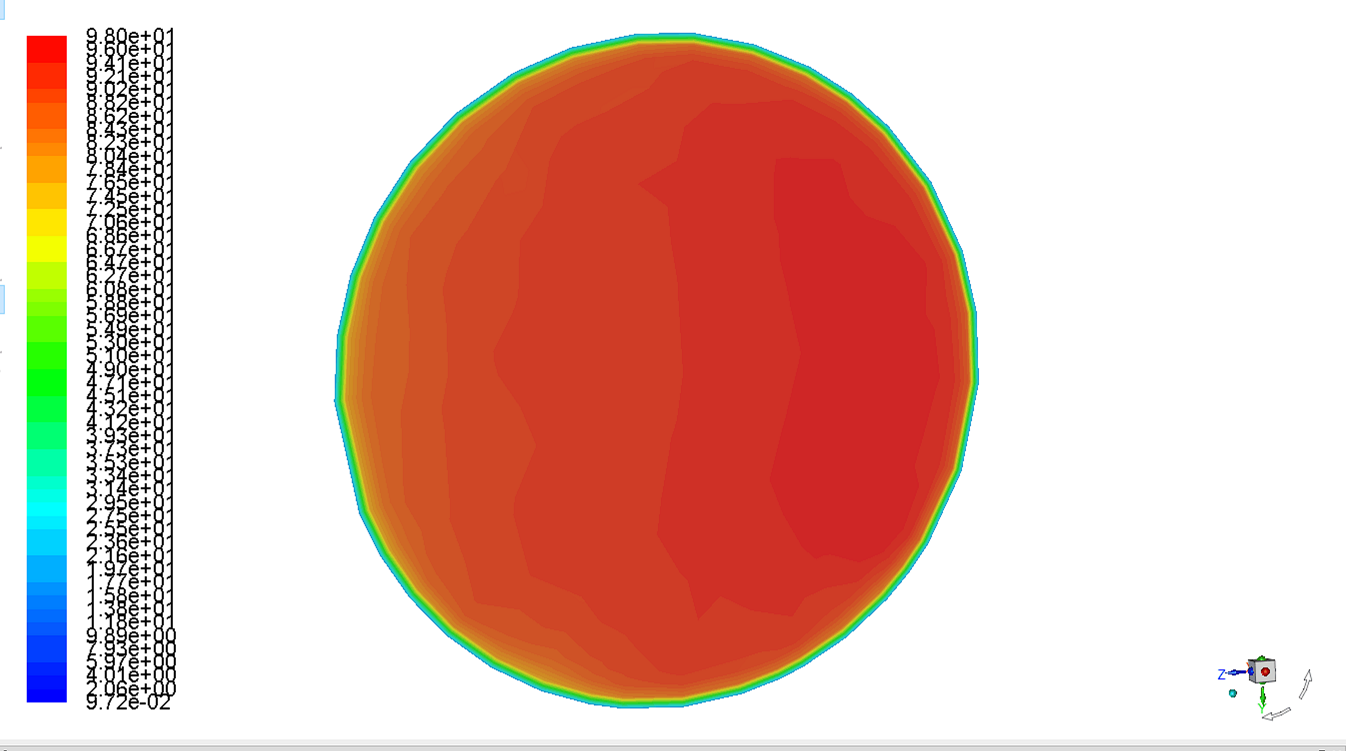  (1) | 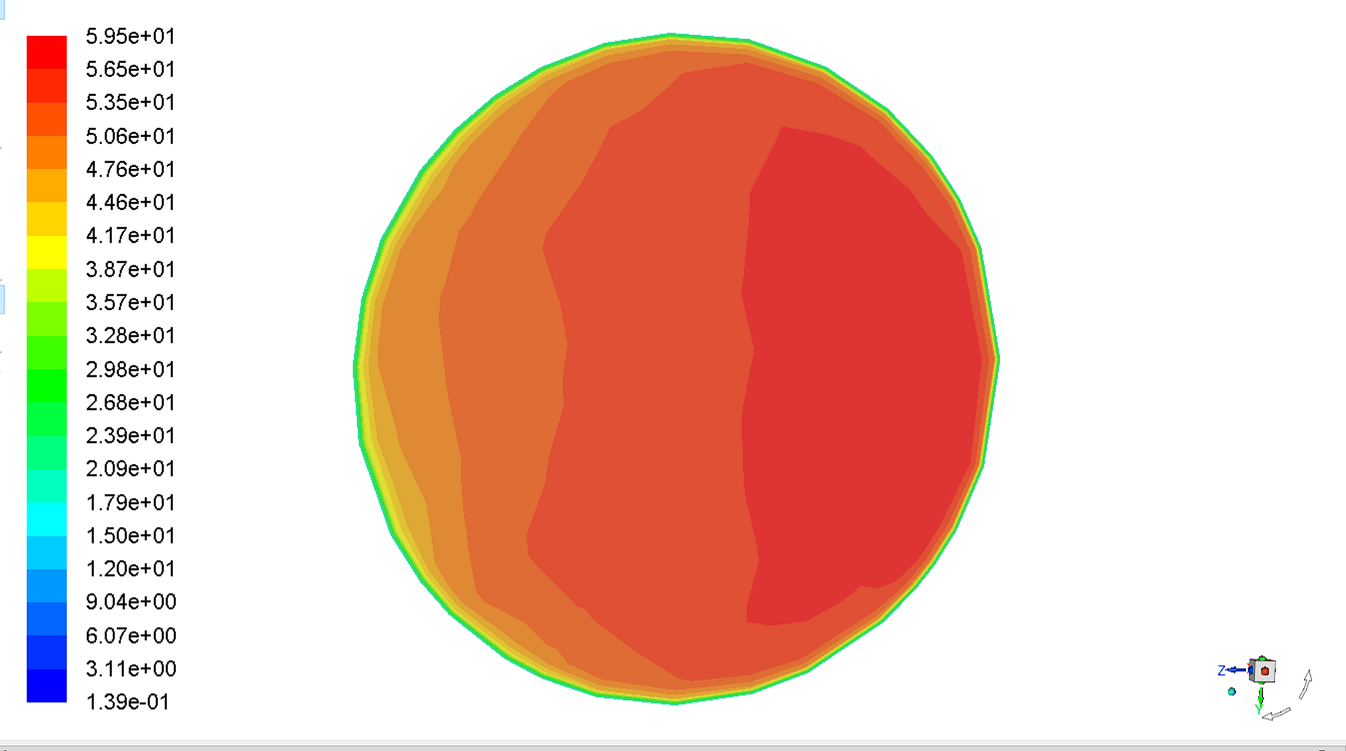  (2) | 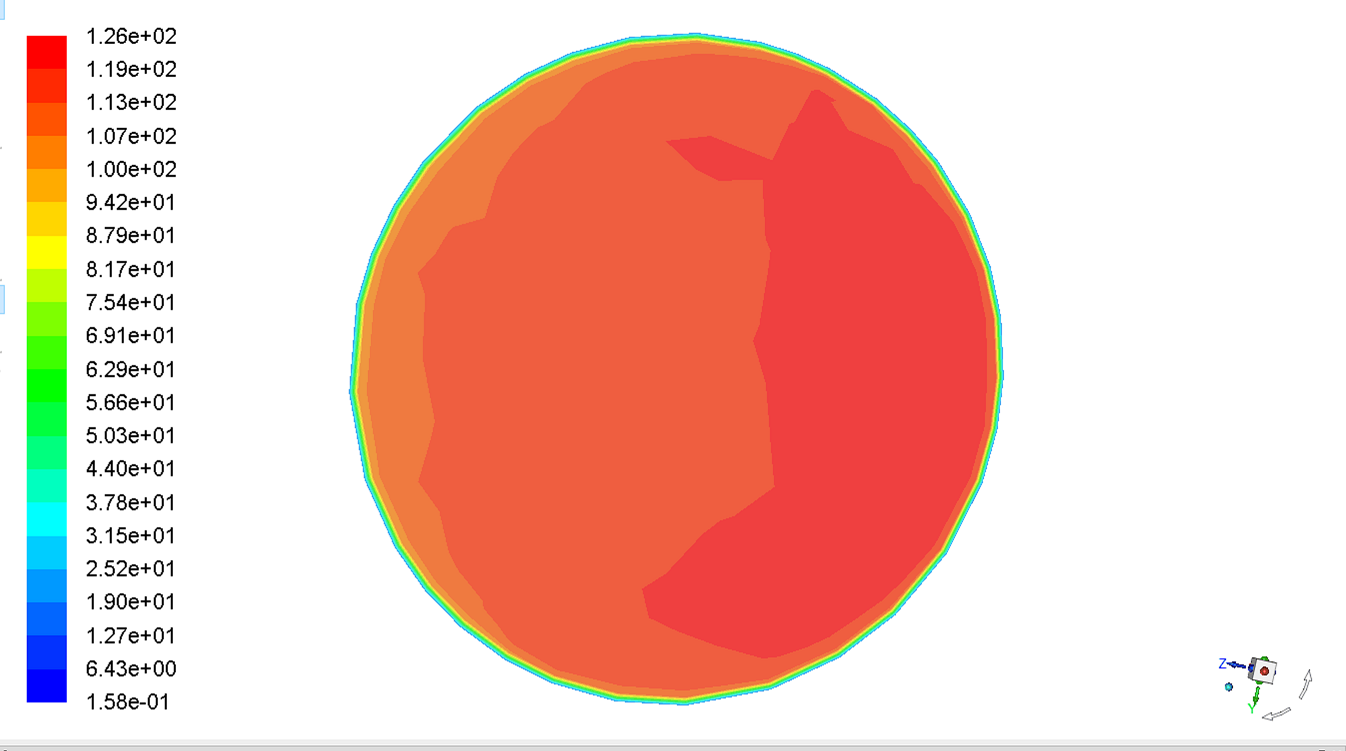  (3) |
| --- | --- | --- |
| 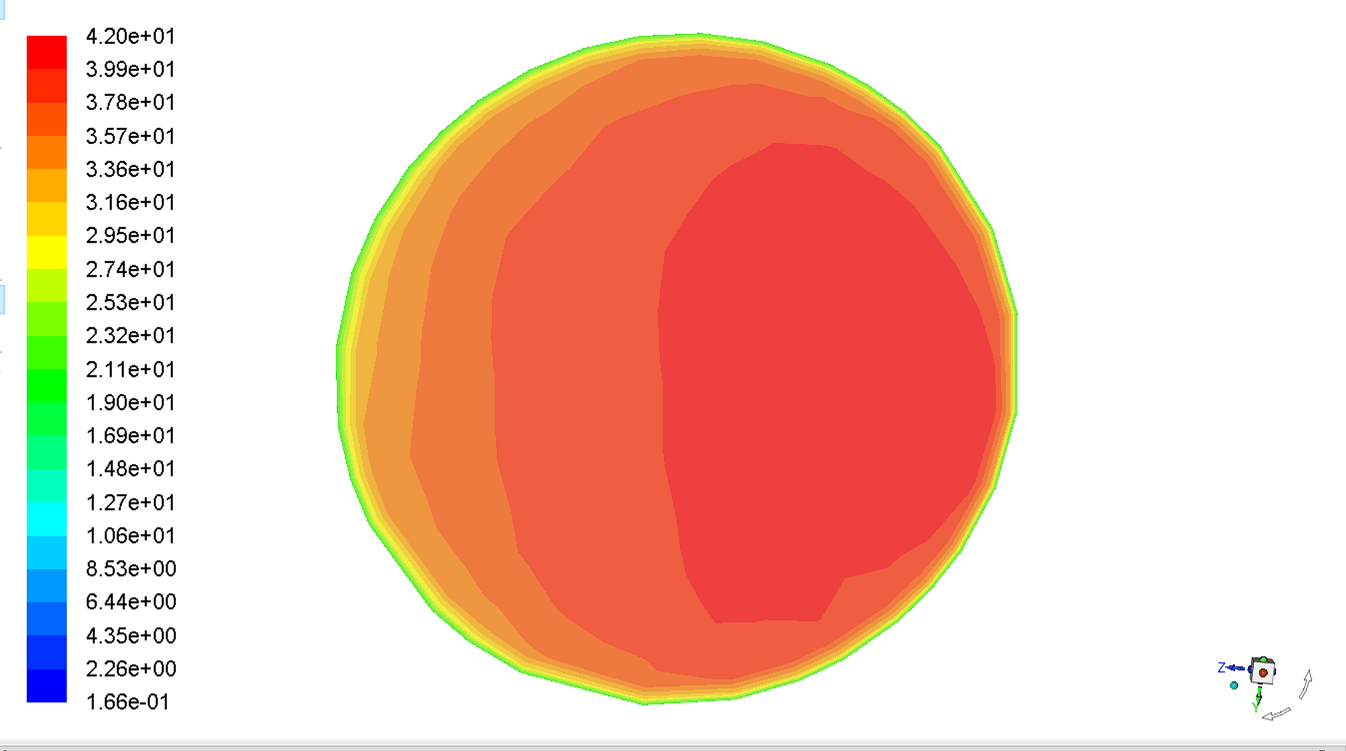  (4) | 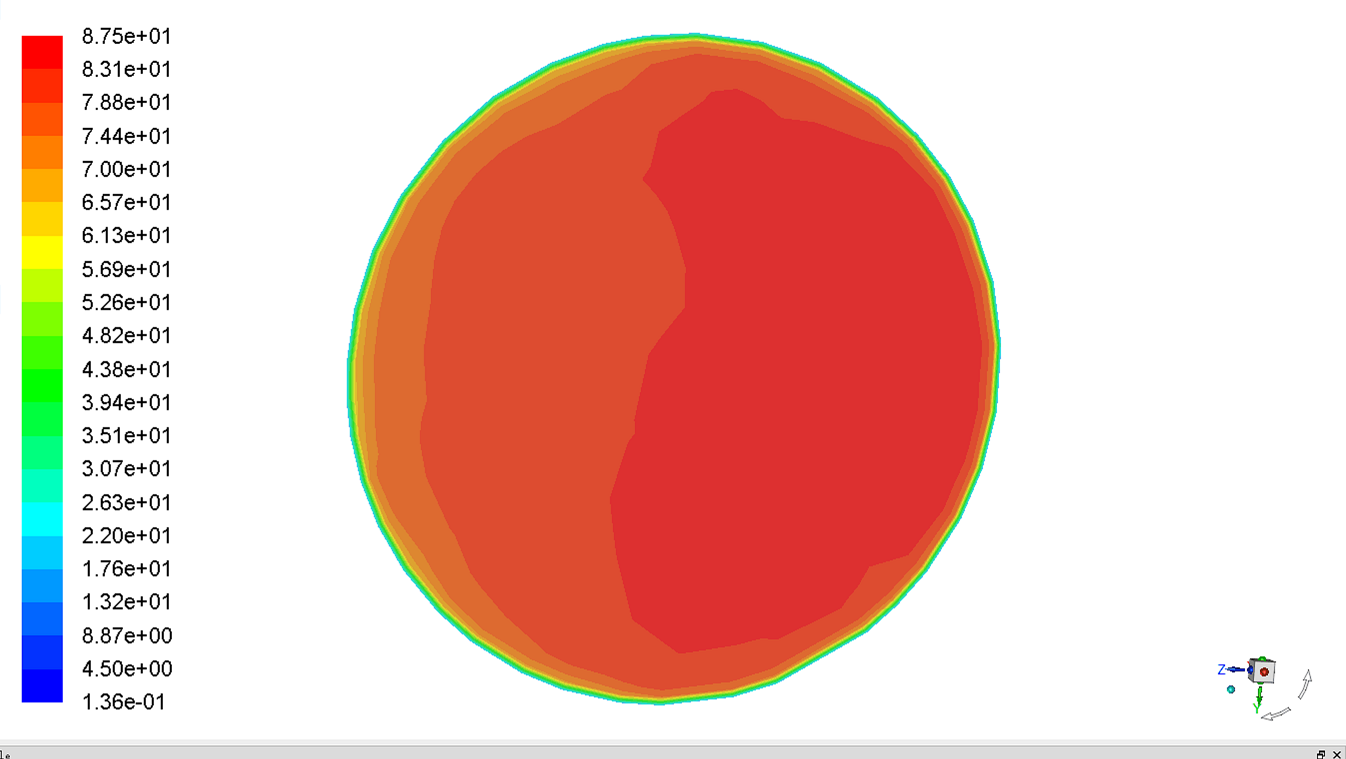  (5) | 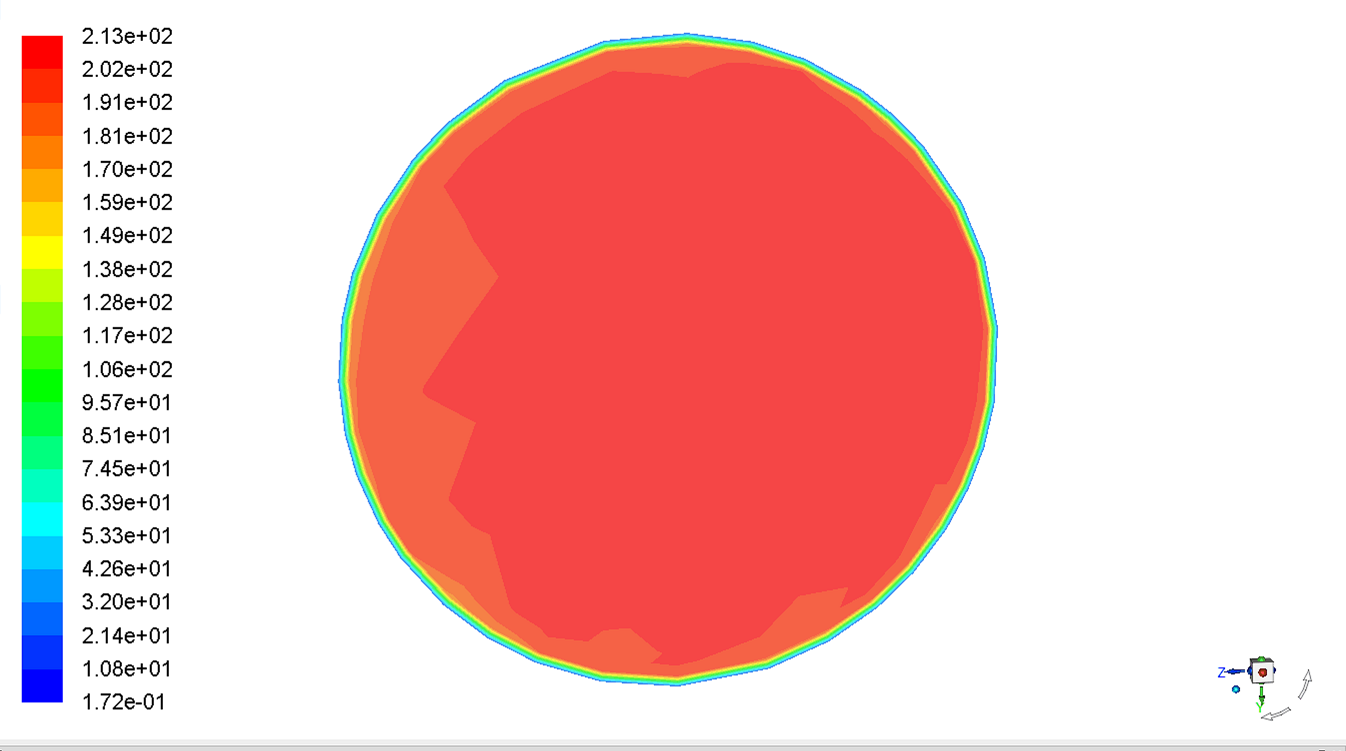  (6) |
| 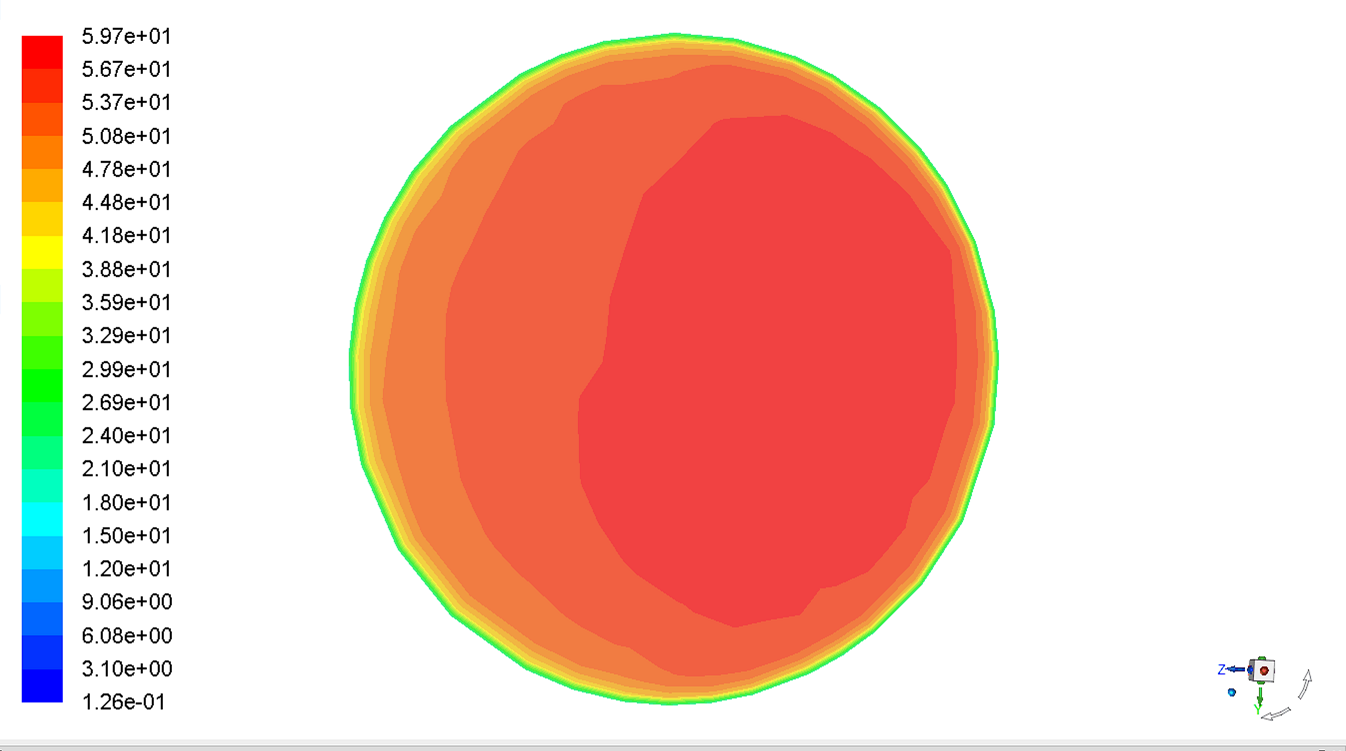  (7) | 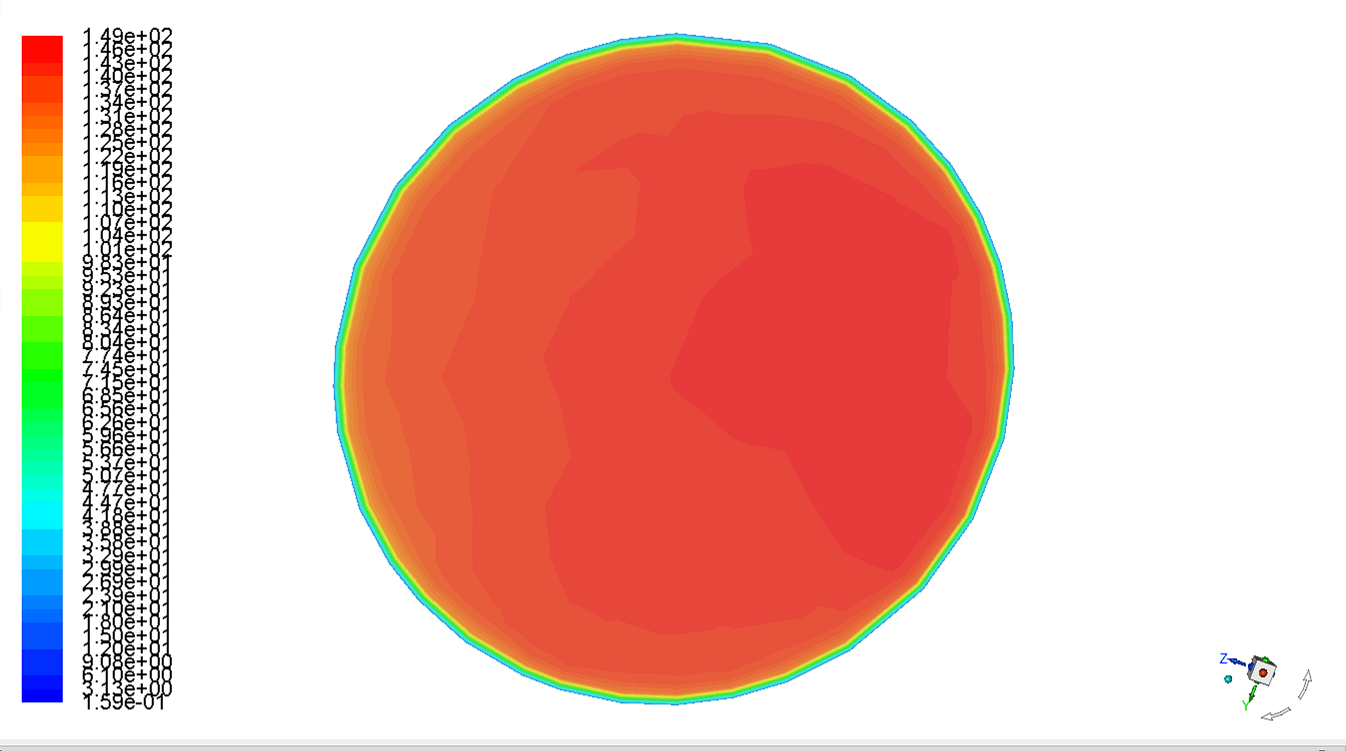  (8) | 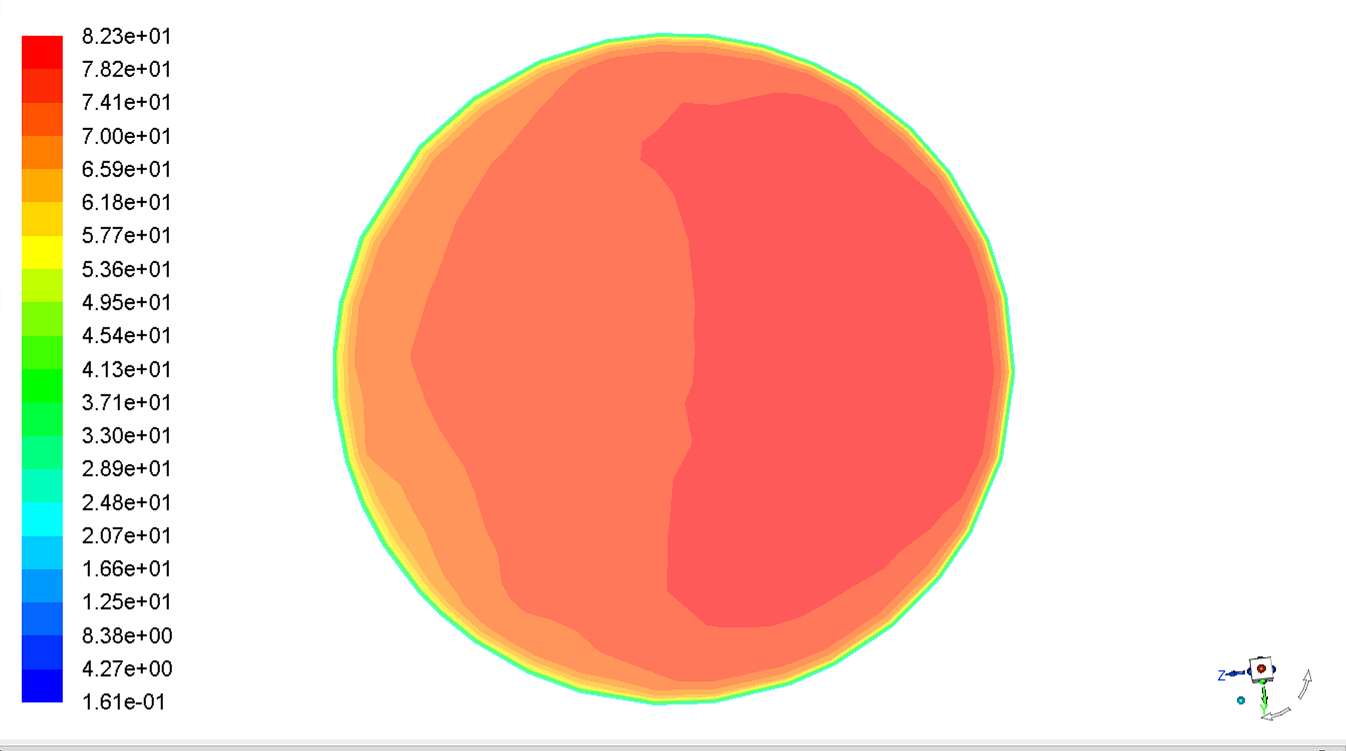  (9) |
| **Figure S13 |** Solution velocity distribution at the outlet section of conical nozzles of 9 tests | | |

| **A**  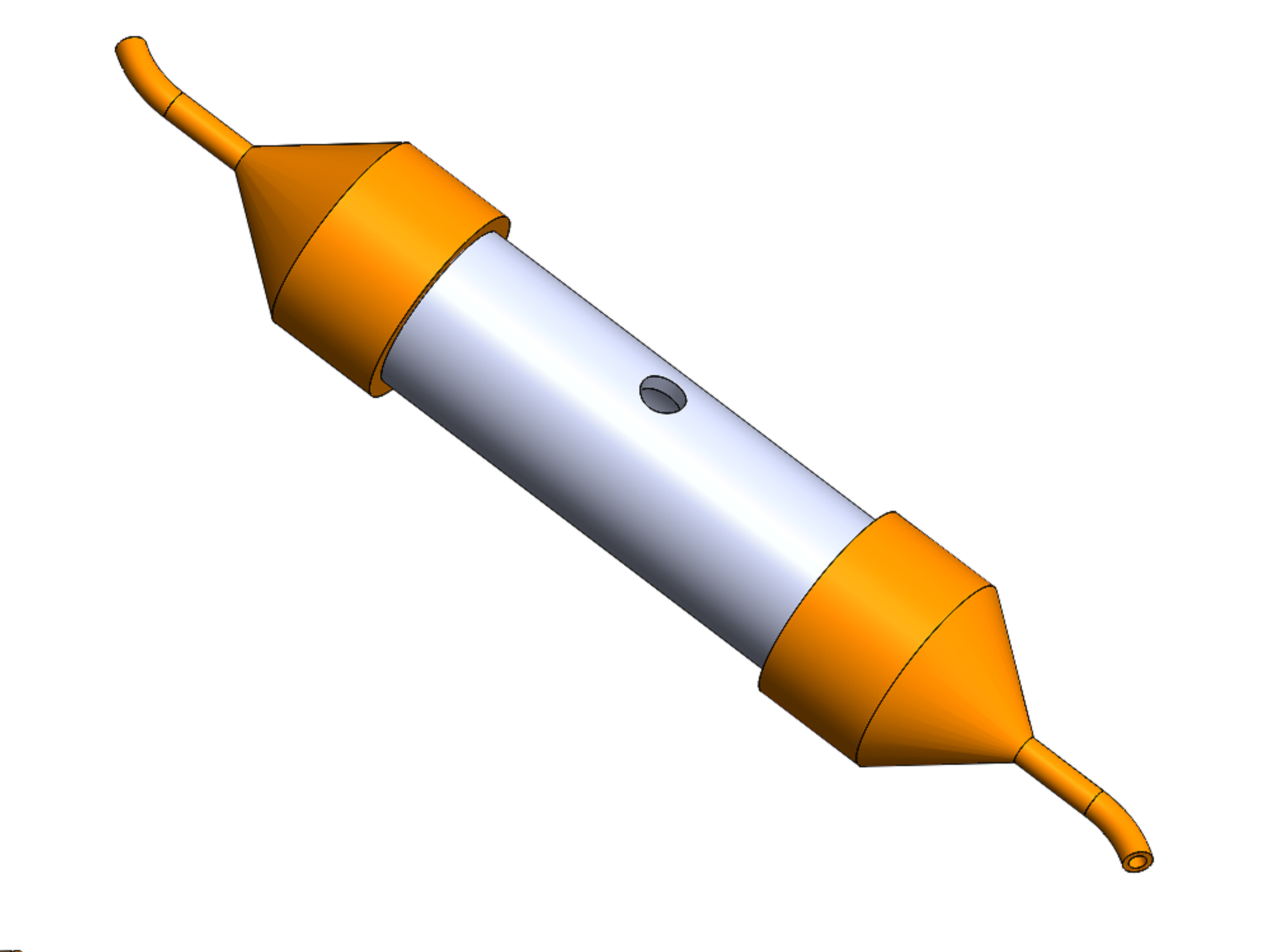 | **B**  **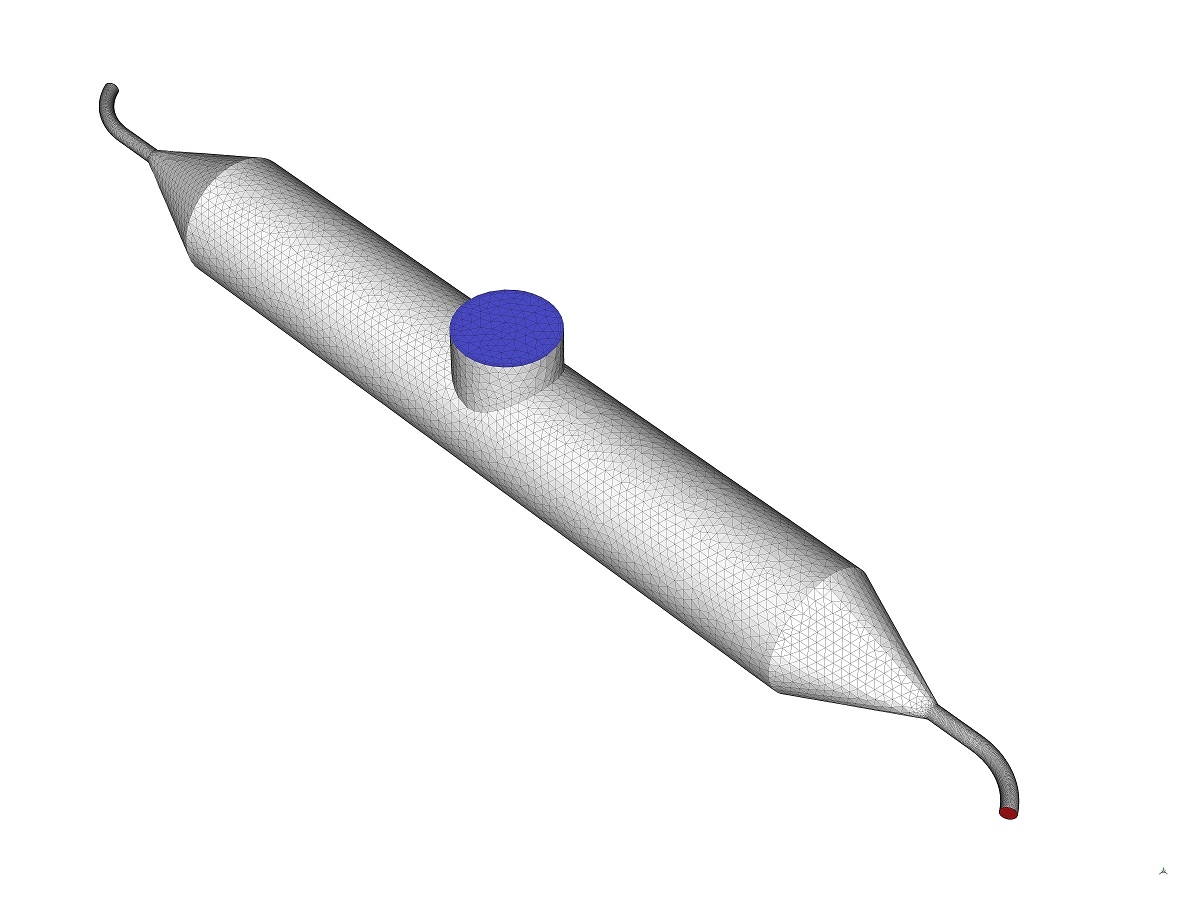** |
| --- | --- |
| **Figure S14 |** Model of the spinneret with curved-pipe nozzles. **(A)** The spinneret with curved-pipe nozzles; **(B)** The mesh division of spinneret with curved-pipe nozzles | |

| 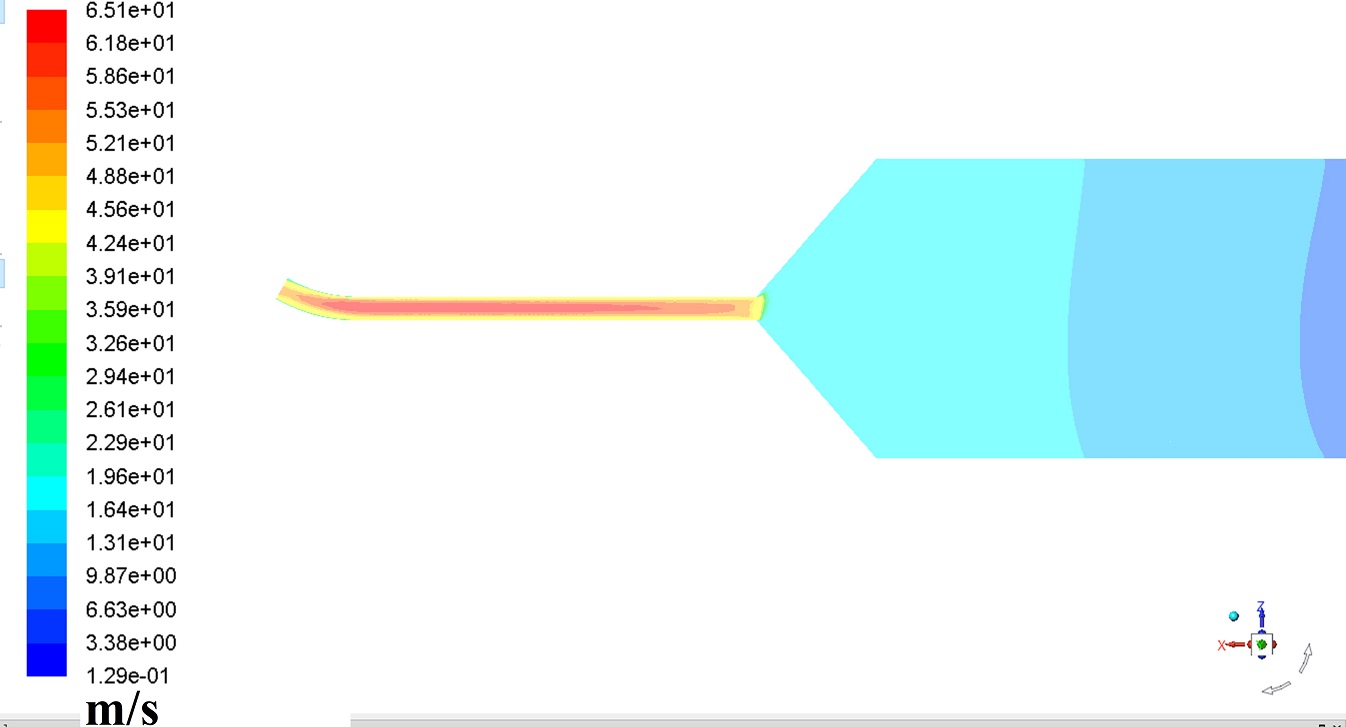 (1) | 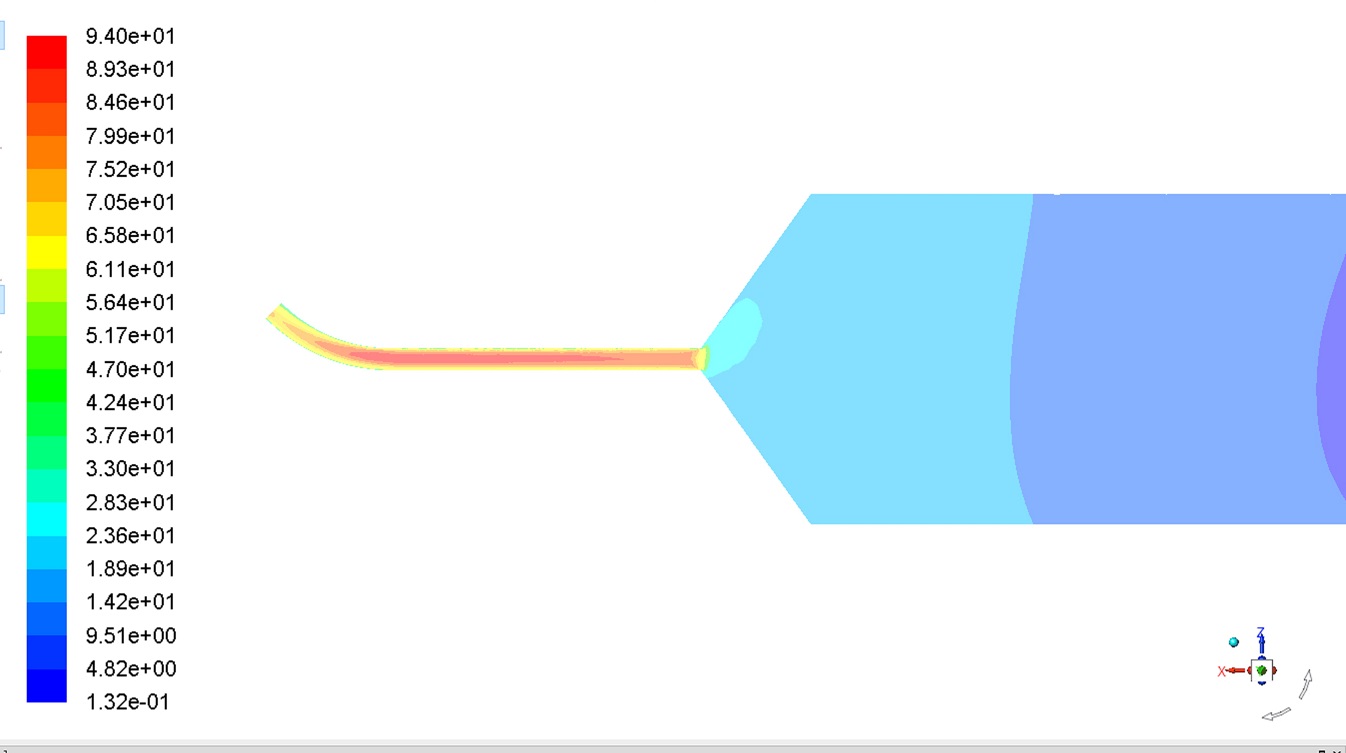  (2) | 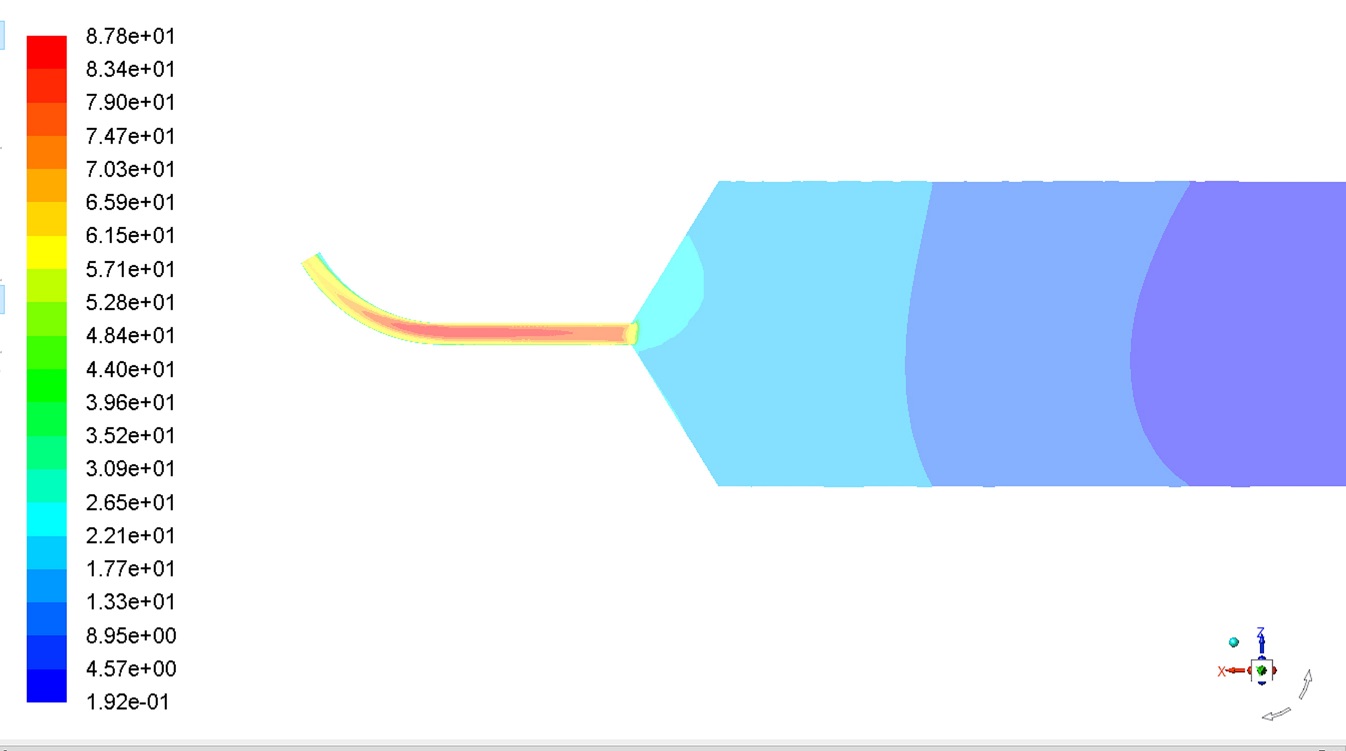  (3) | 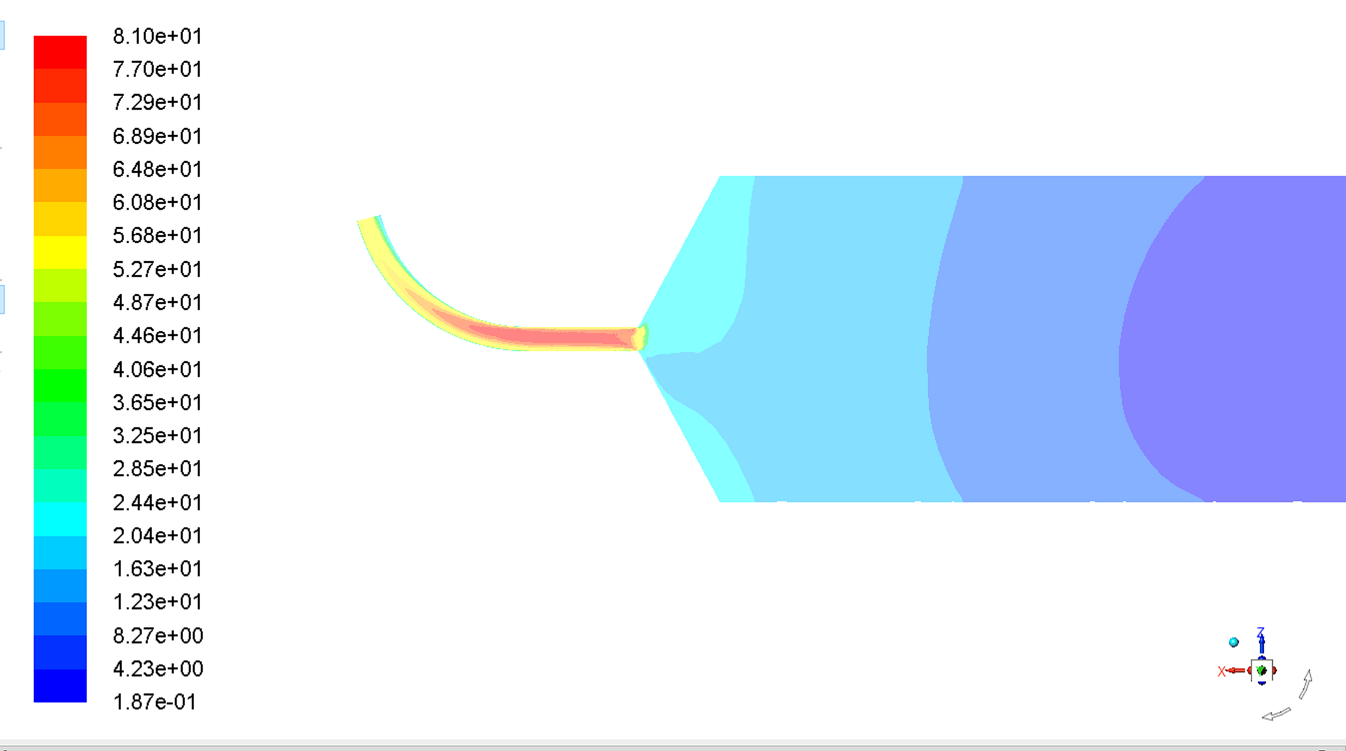  (4) |
| --- | --- | --- | --- |
| 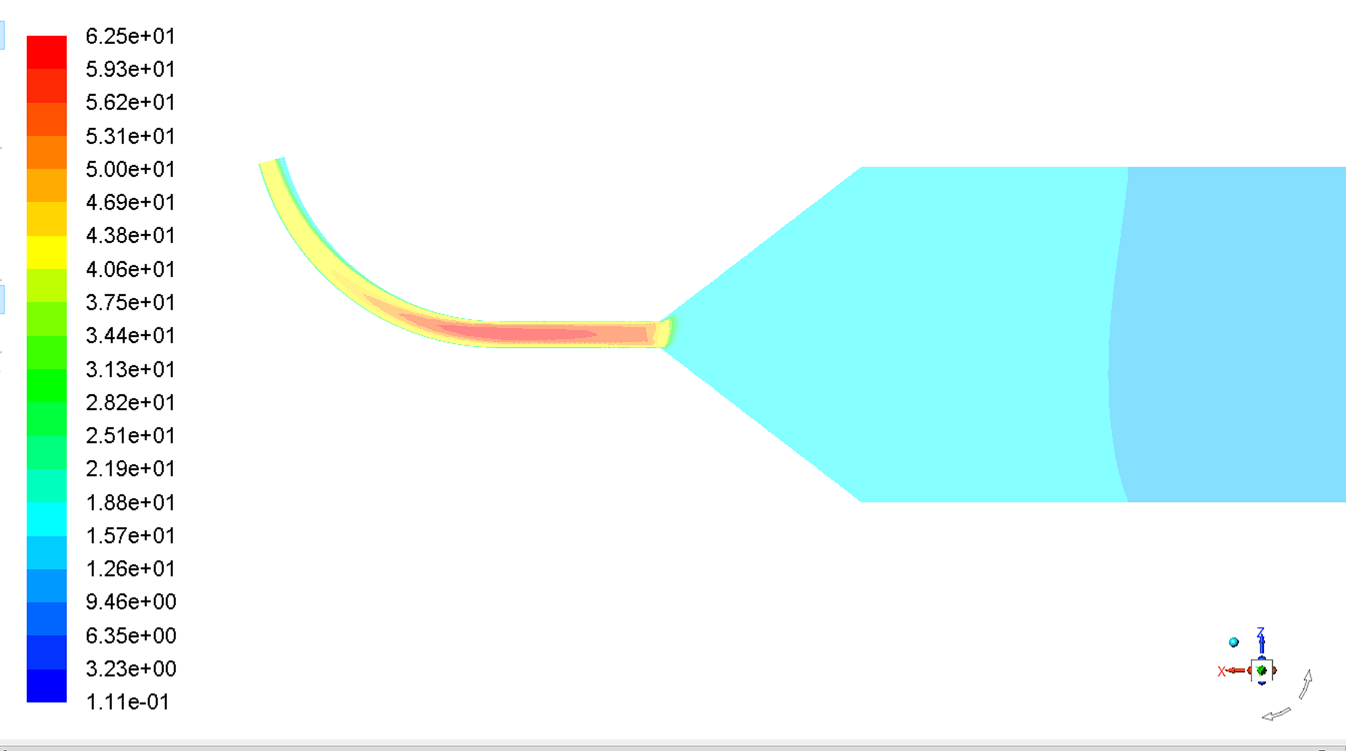  (5) | 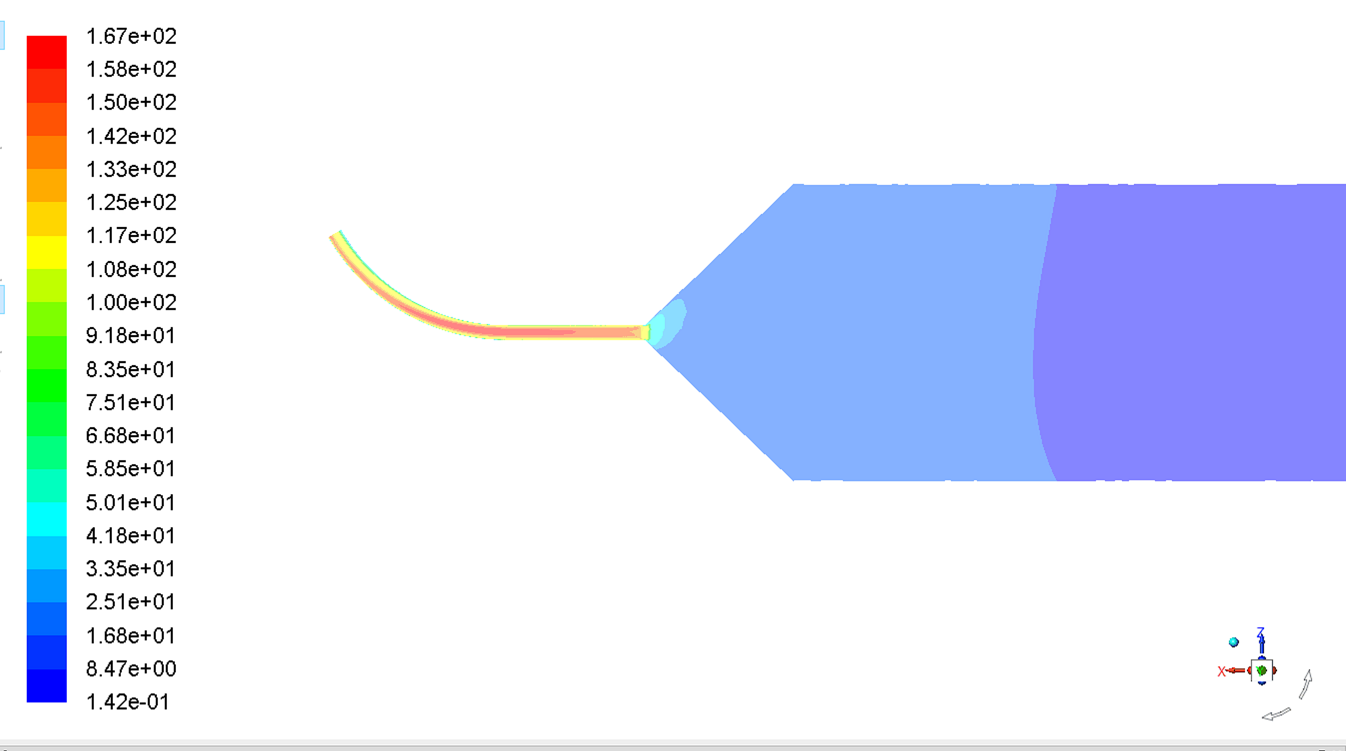  (6) | 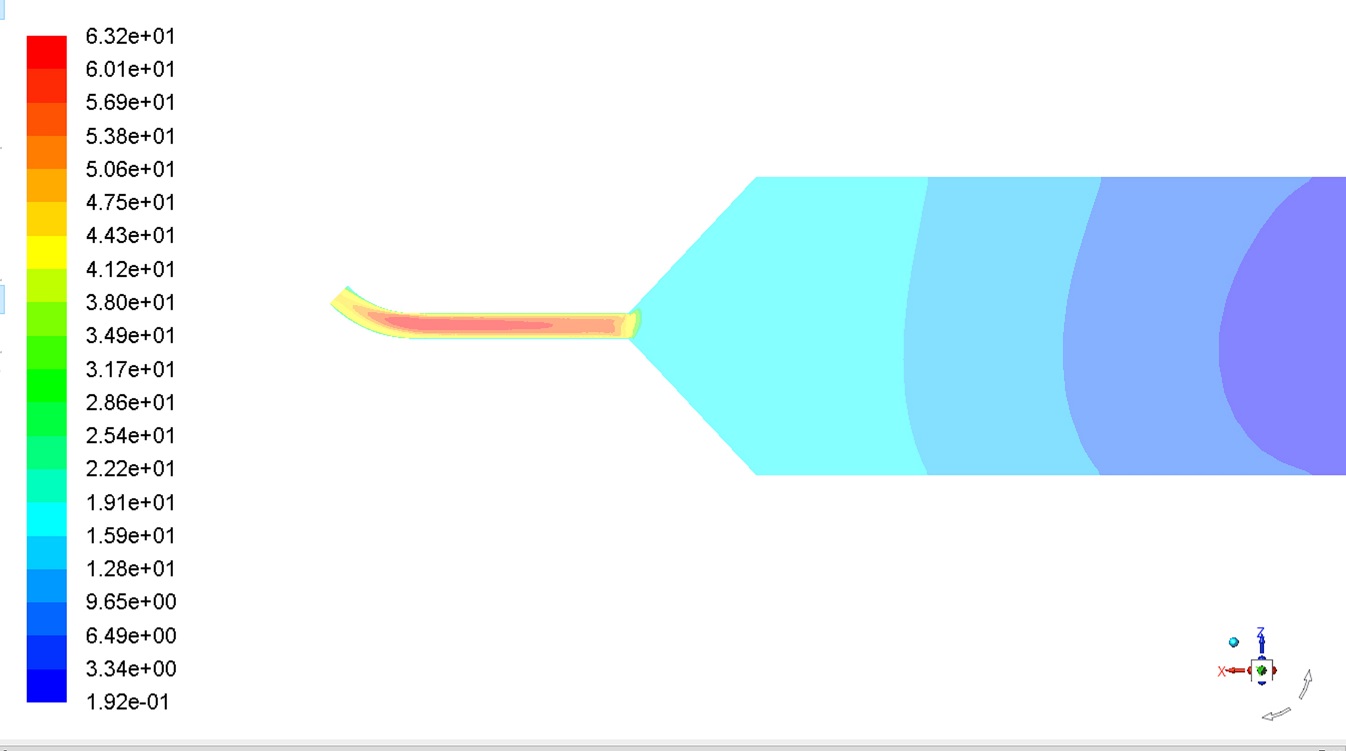  (7) | 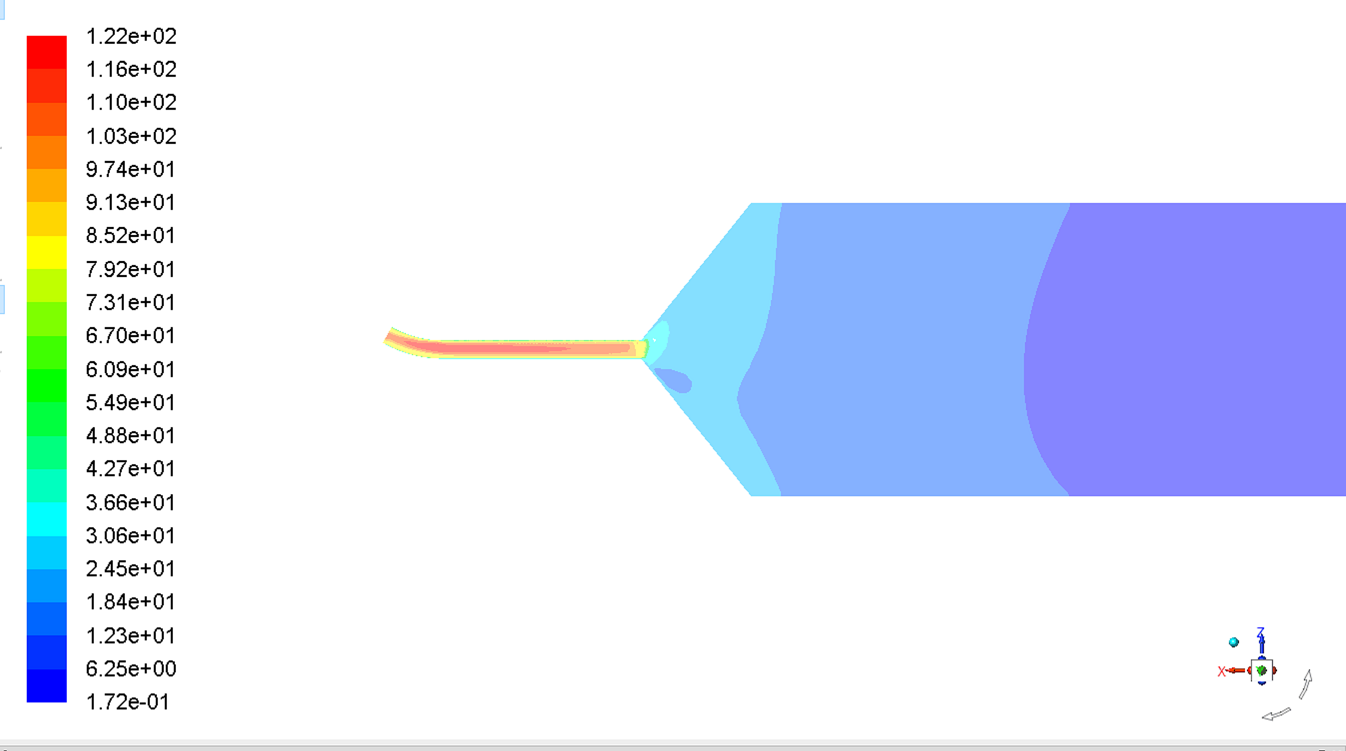  (8) |
| 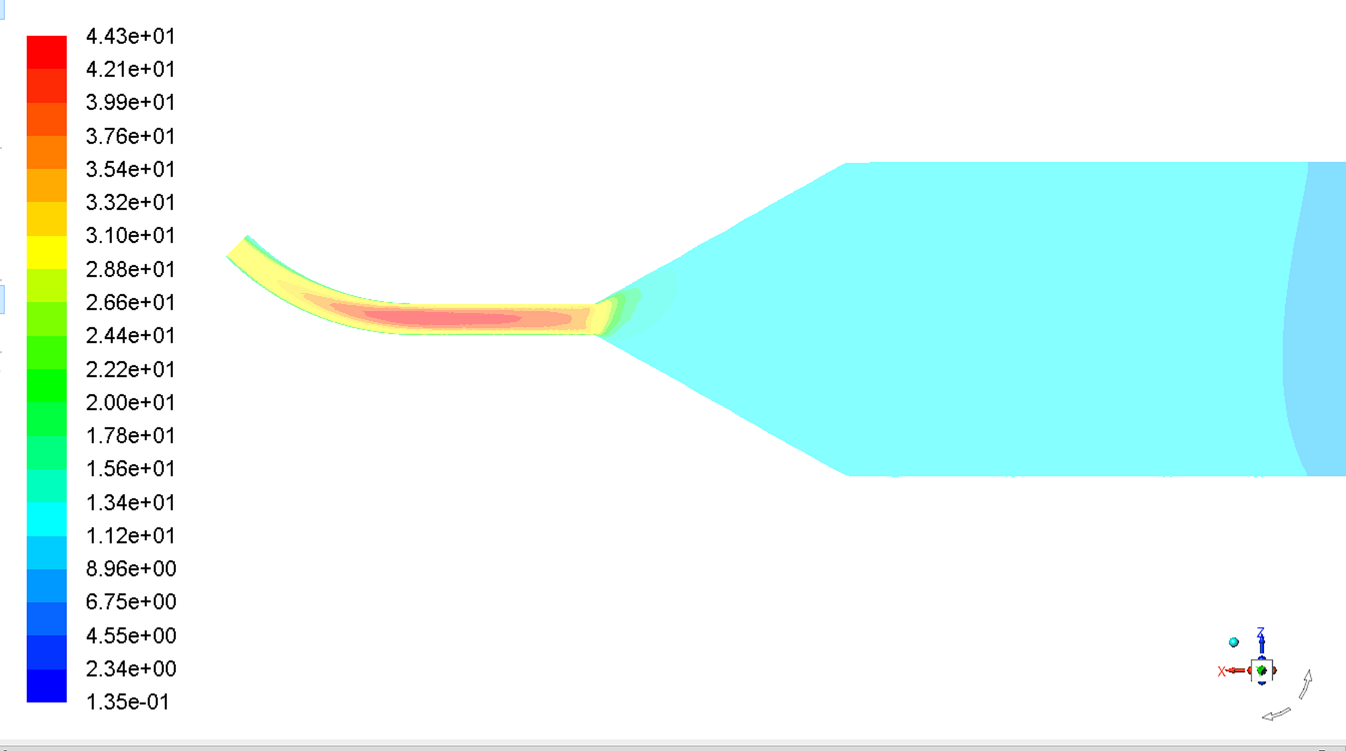  (9) | 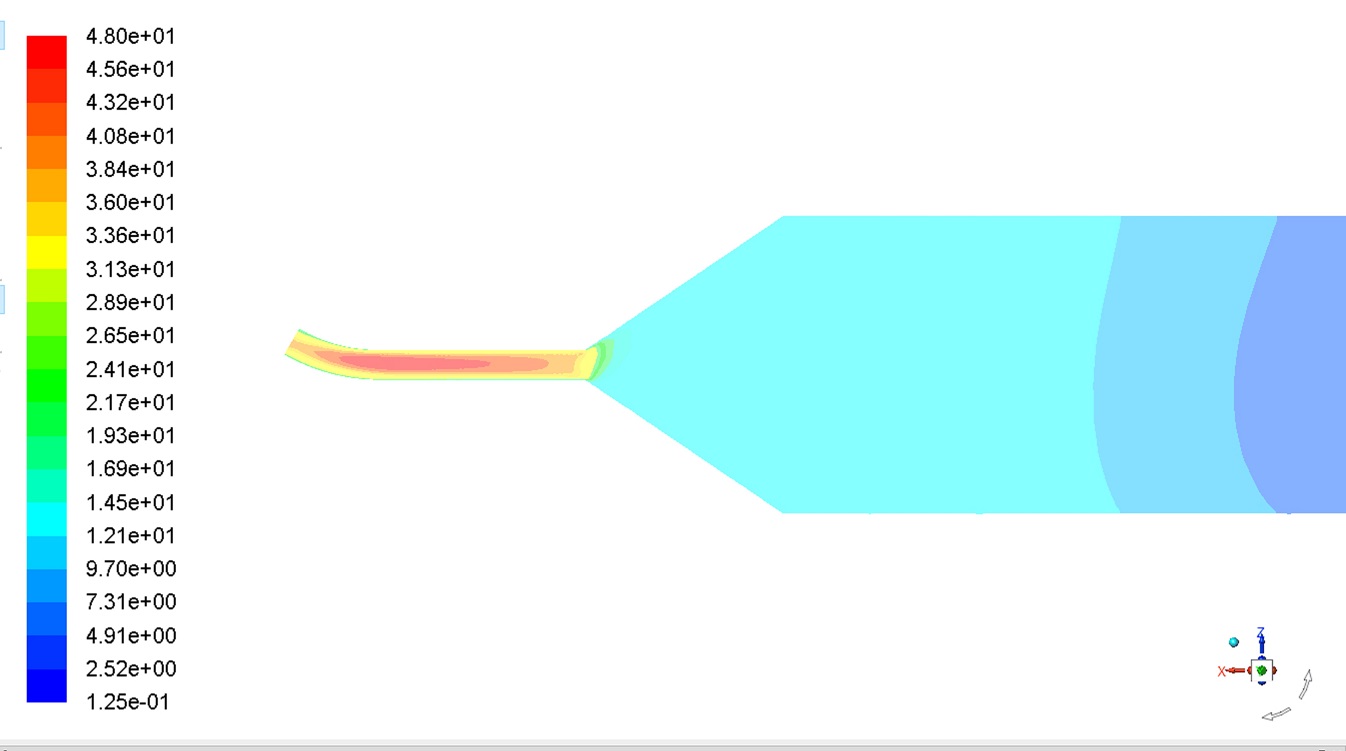  (10) | 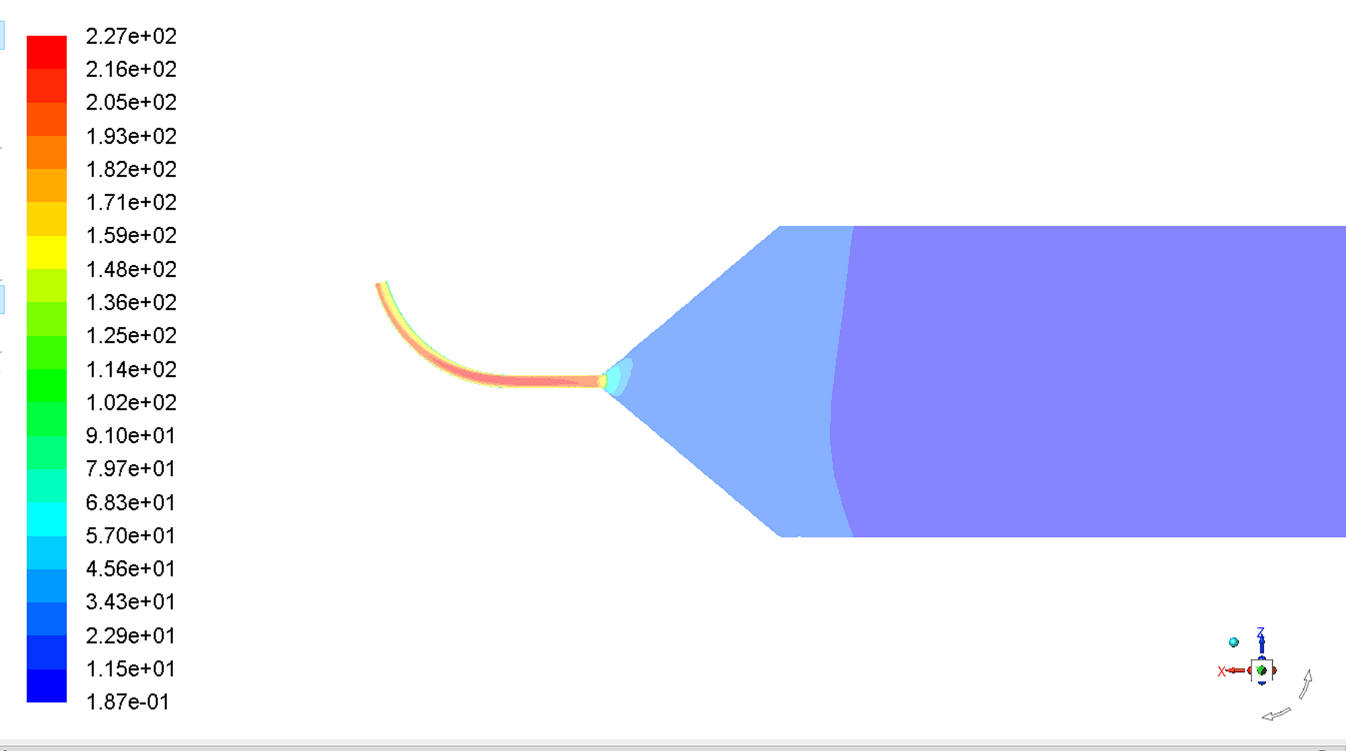  (11) | 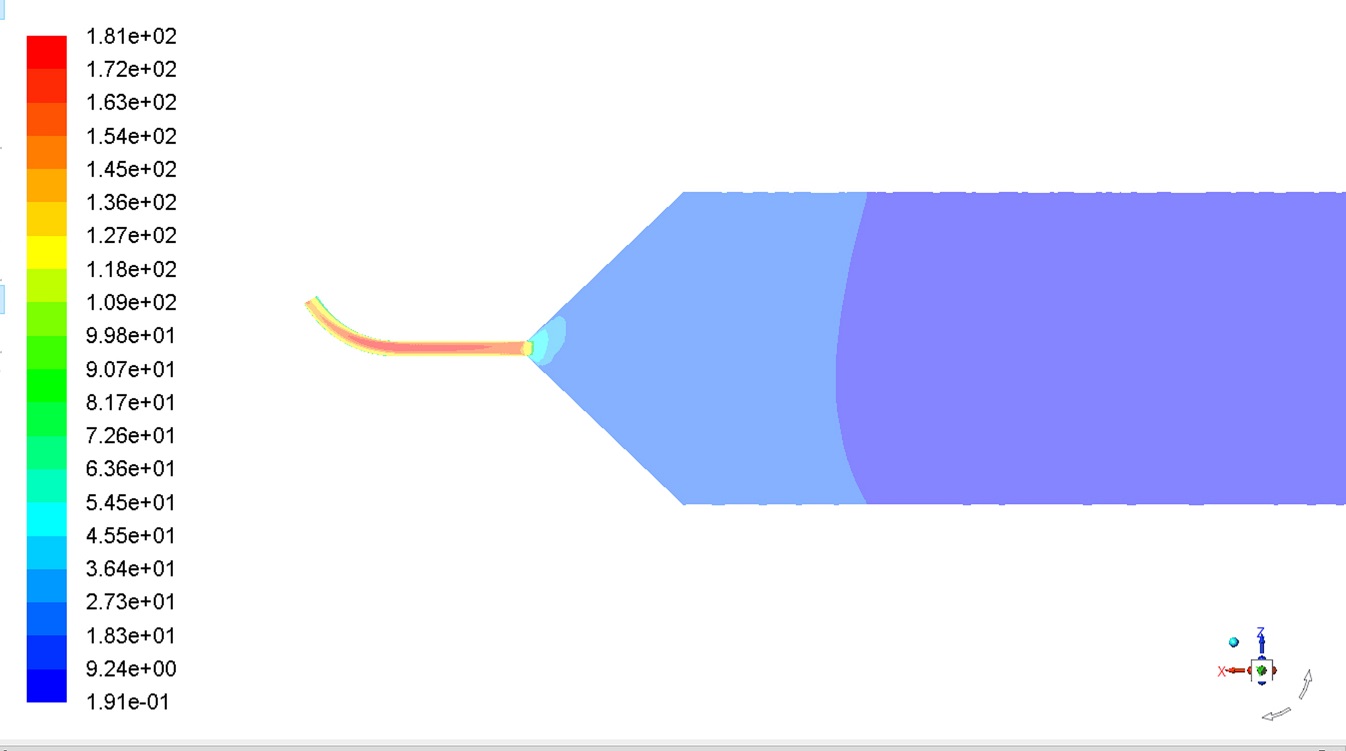  (12) |
| 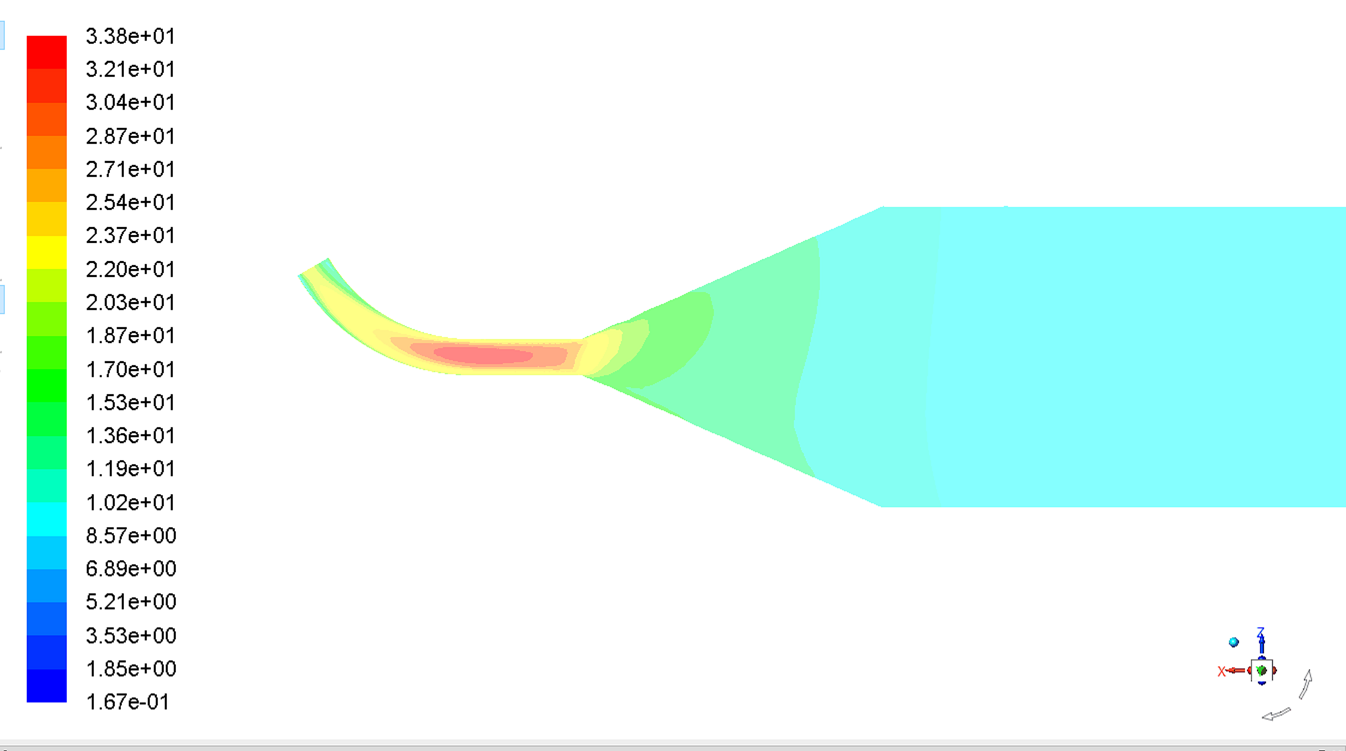  (13) | 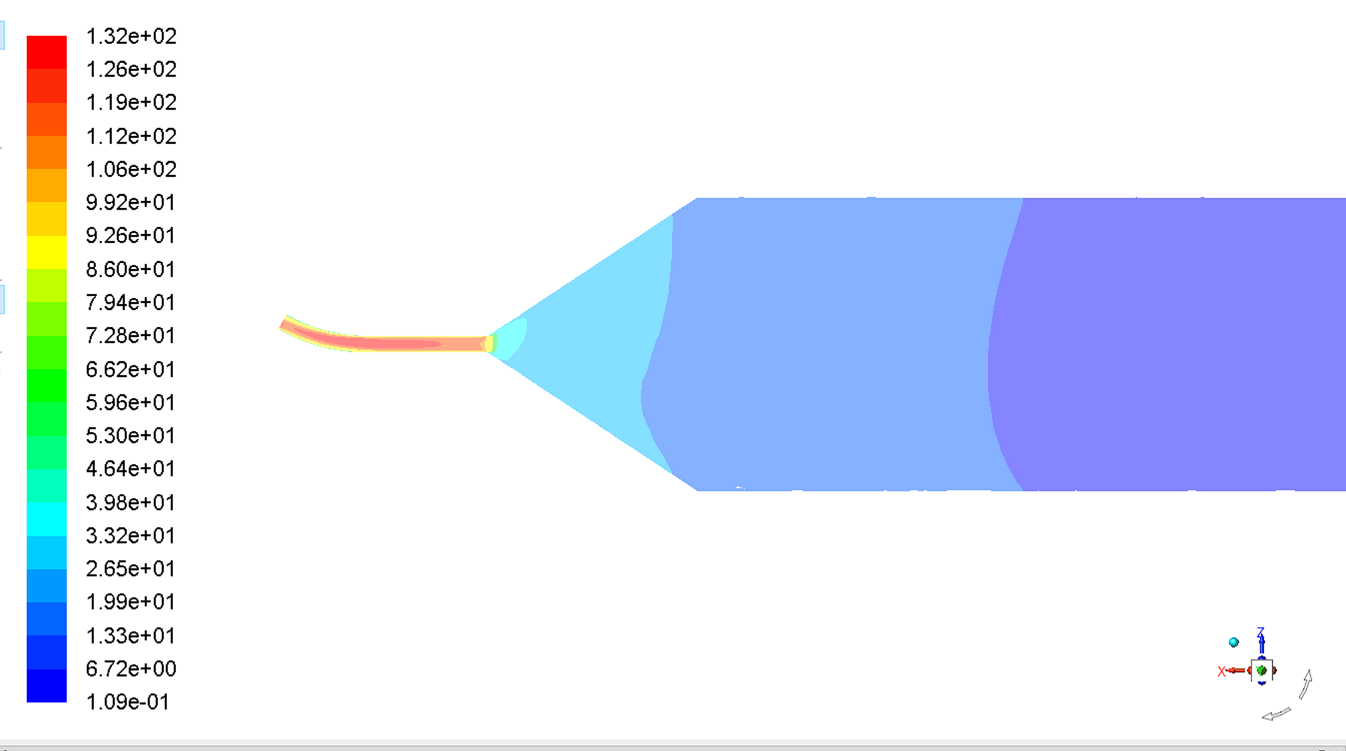  (14) | 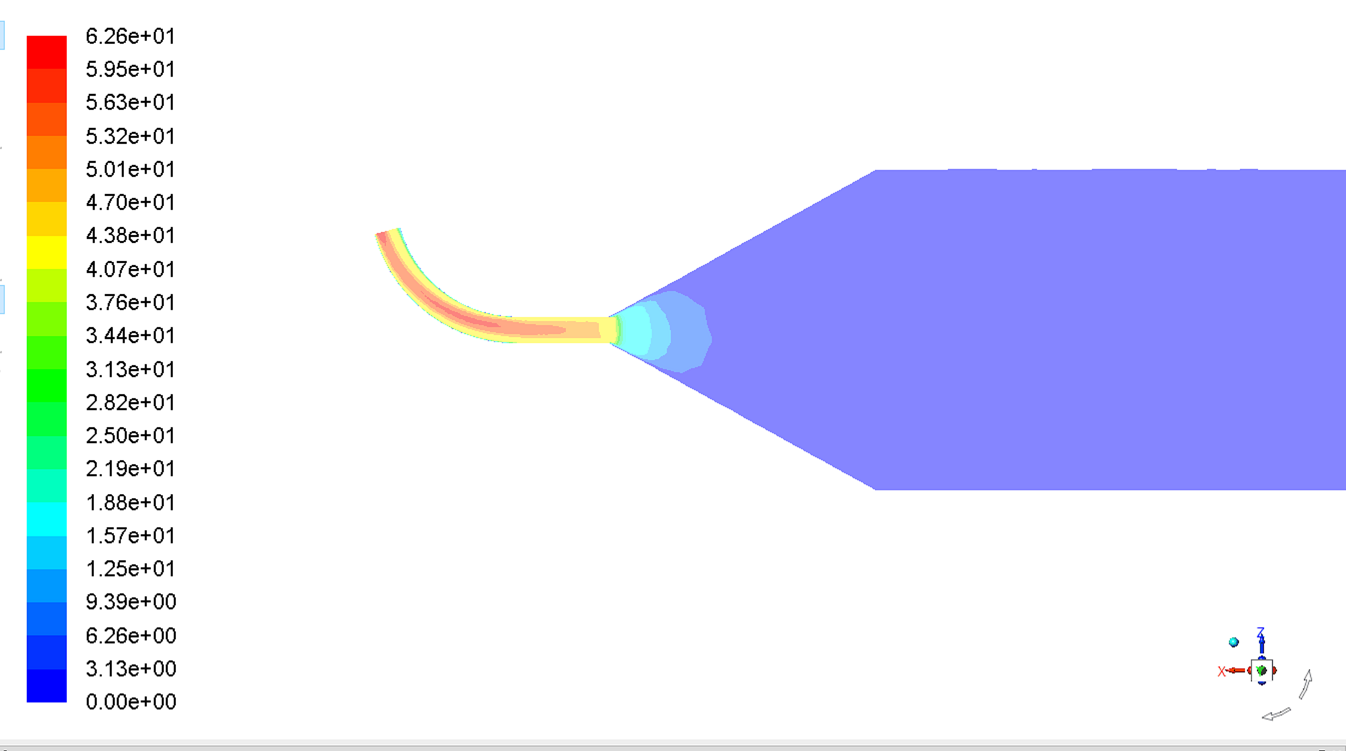  (15) | 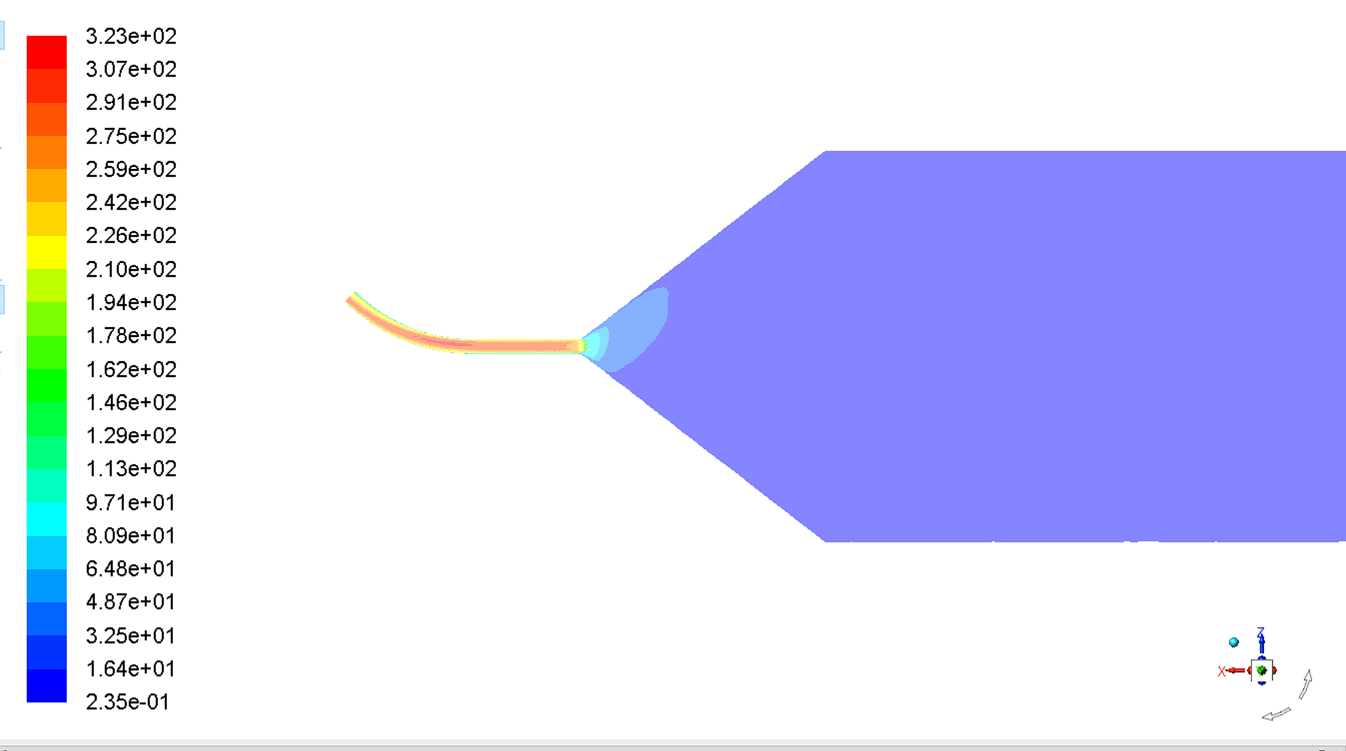  (16) |
| **Figure S15 |** Cloud diagram of solution velocity distribution in curved-pipe nozzles of 16 tests | | | |

| 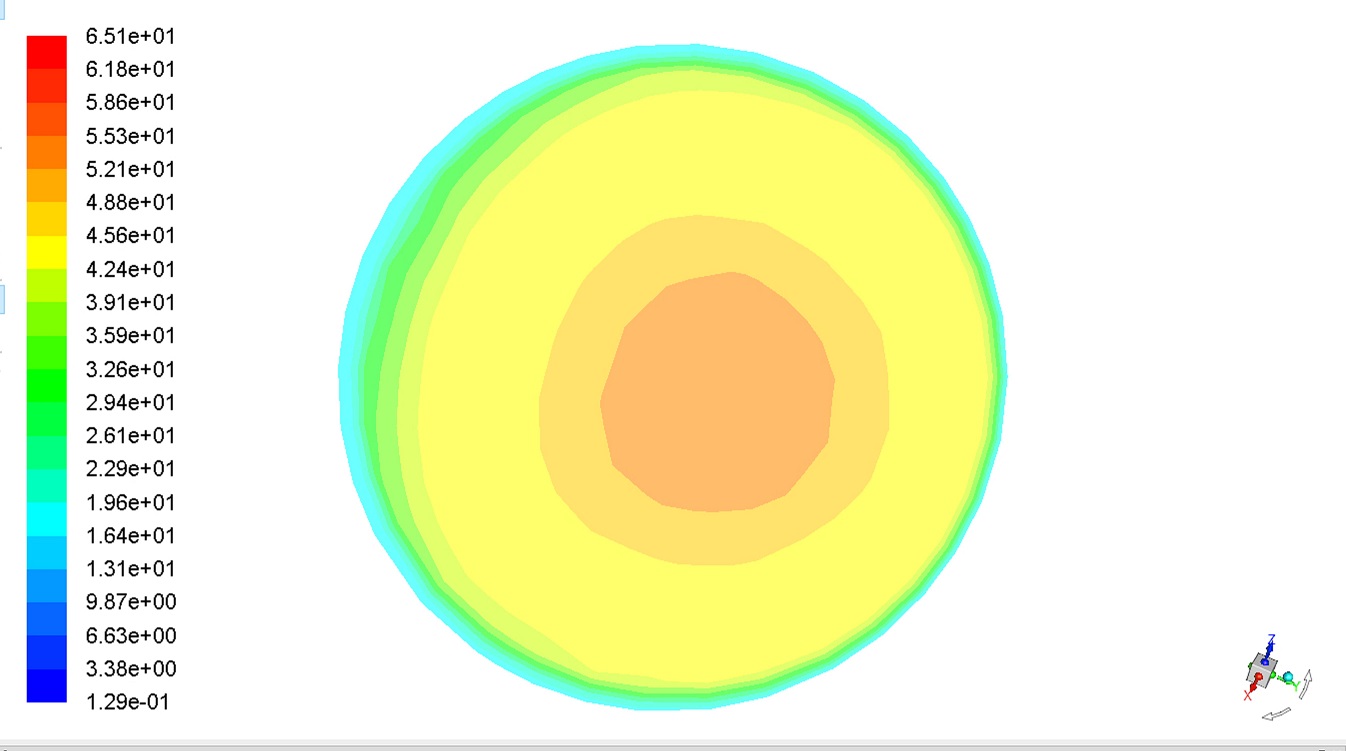  (1) | 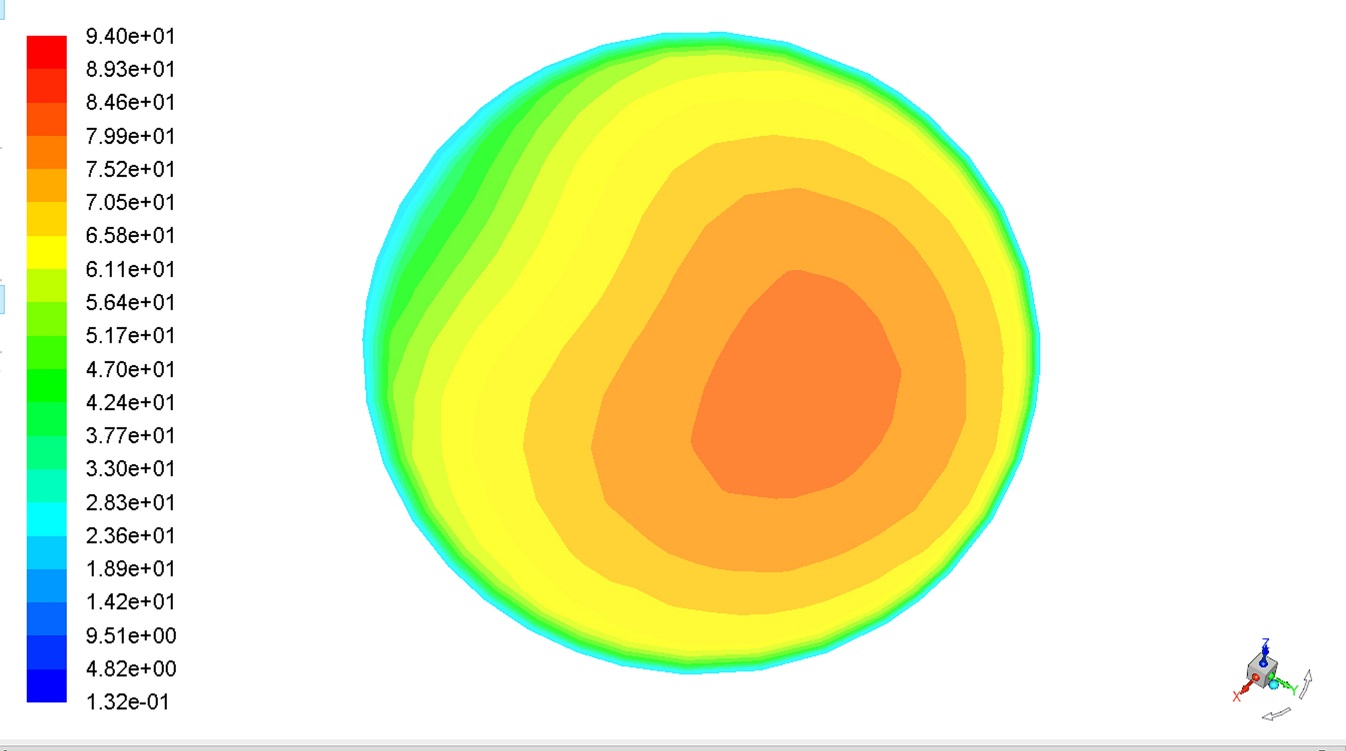  (2) | 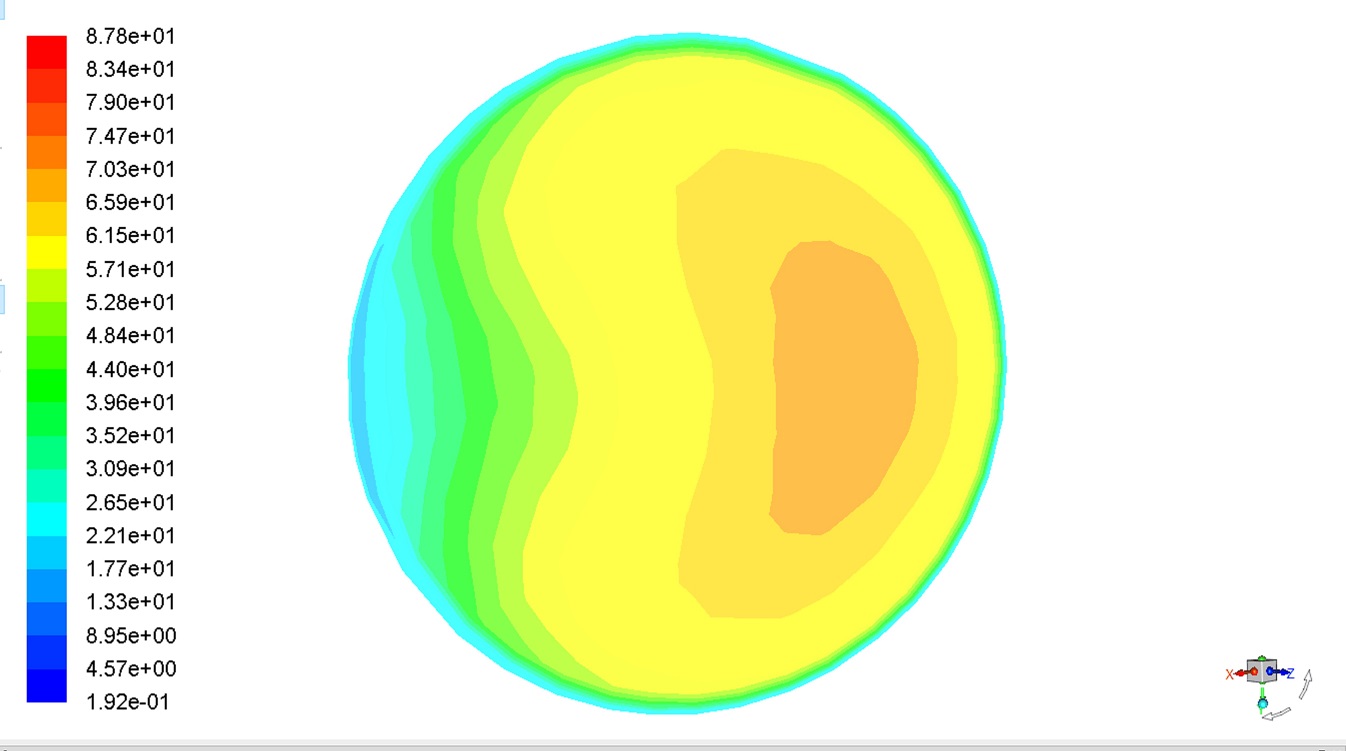  (3) | 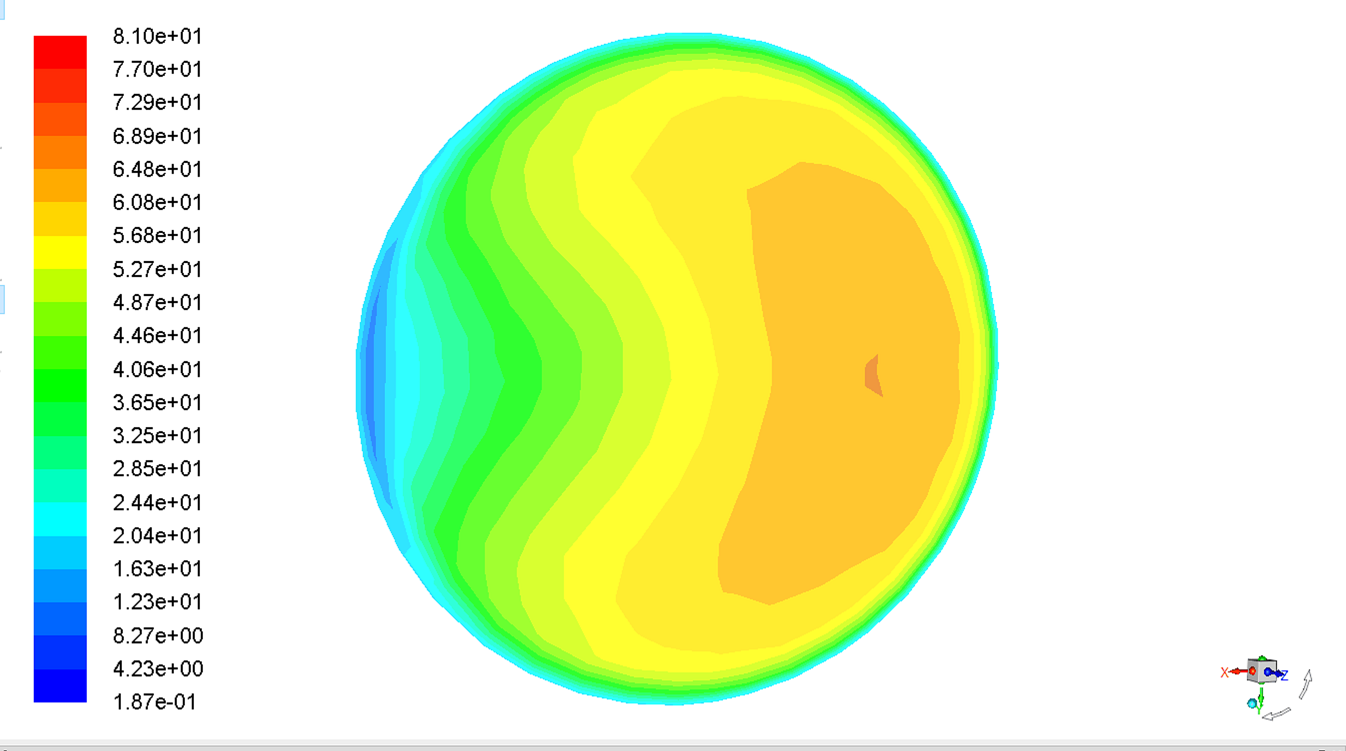  (4) |
| --- | --- | --- | --- |
| 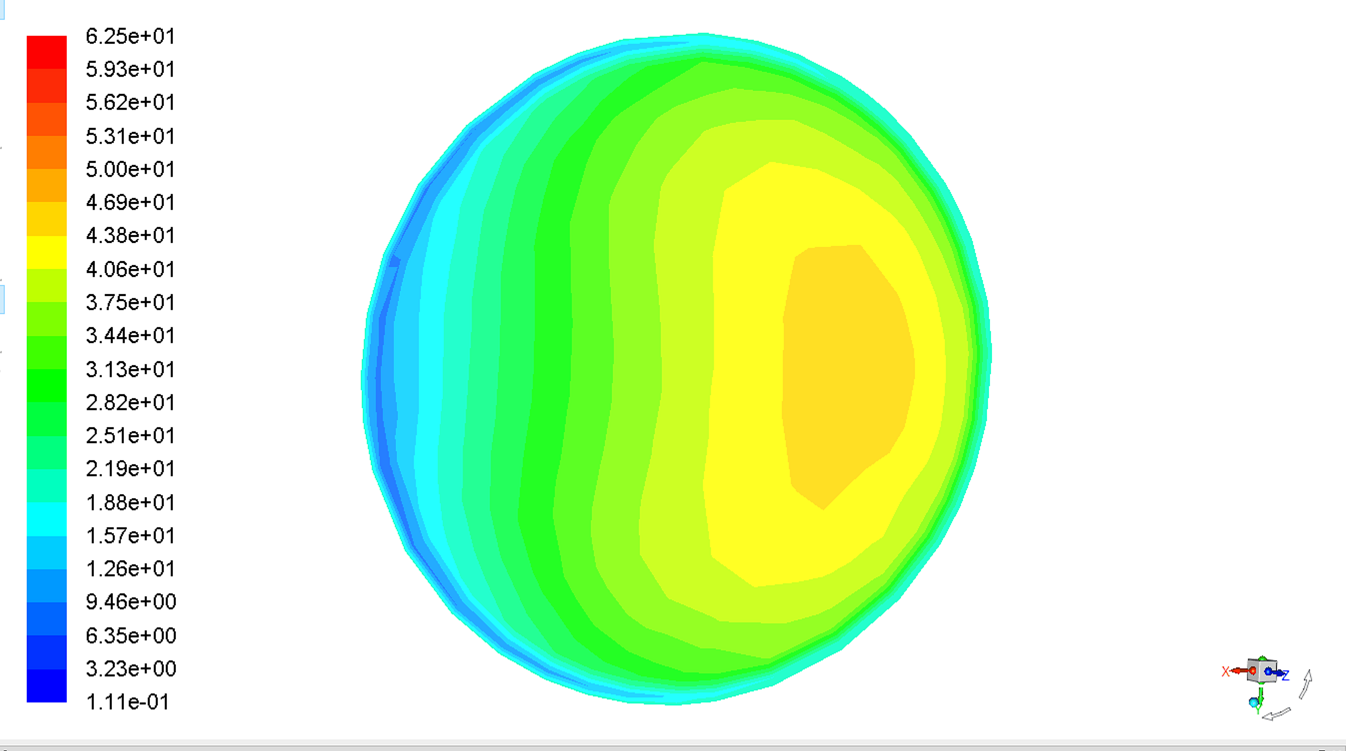  (5) | 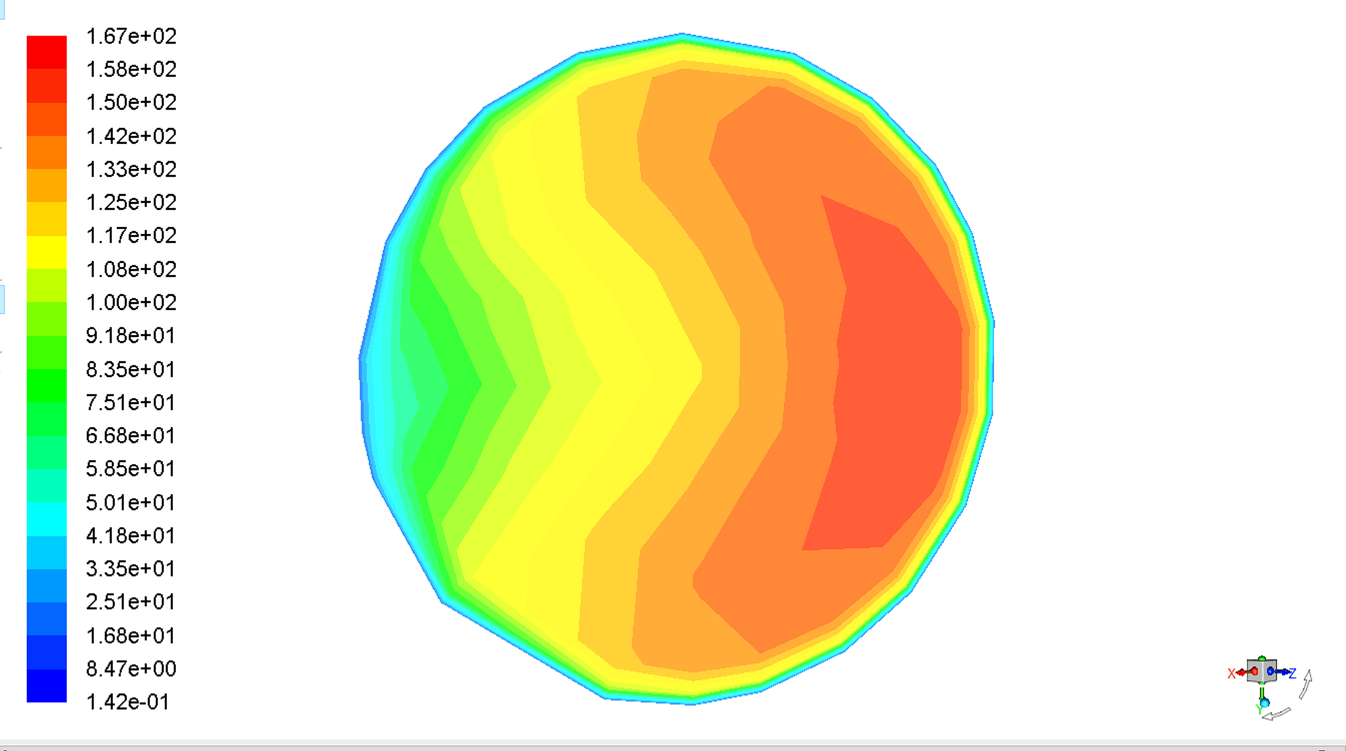  (6) | 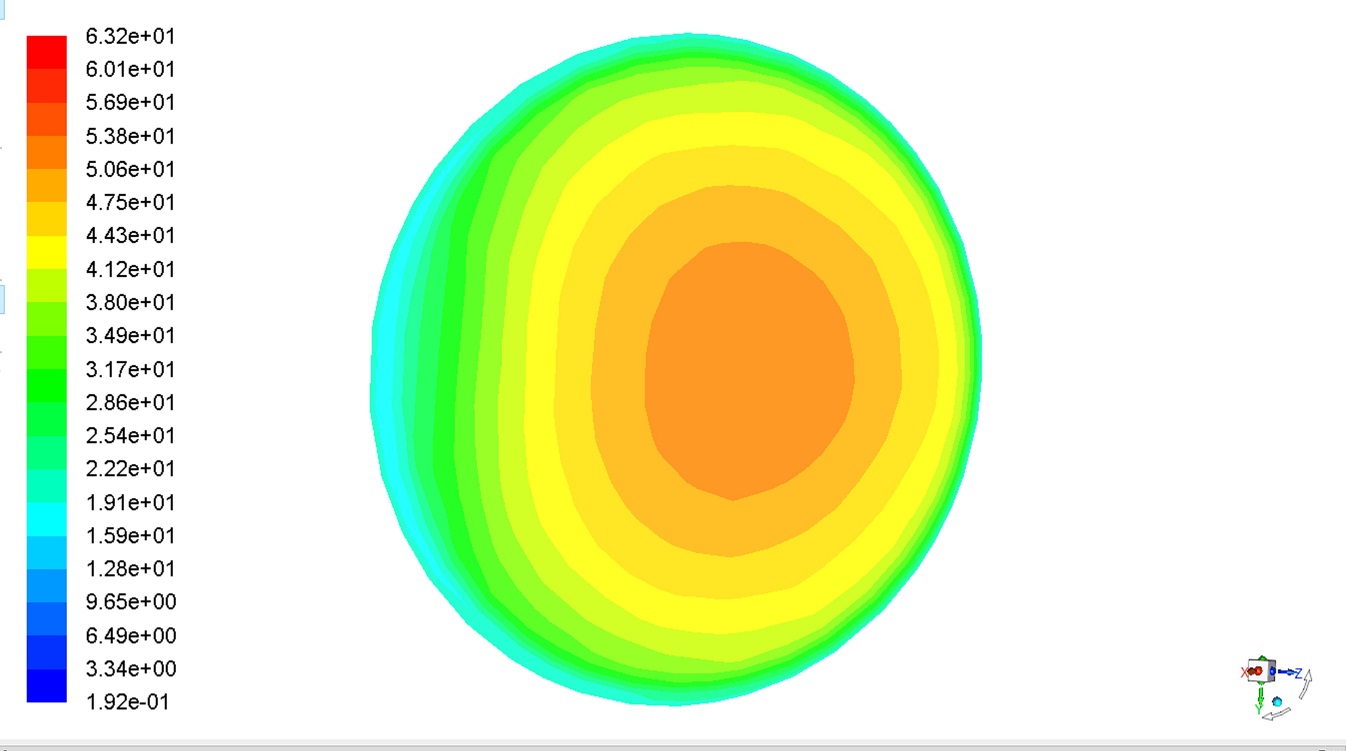  (7) | 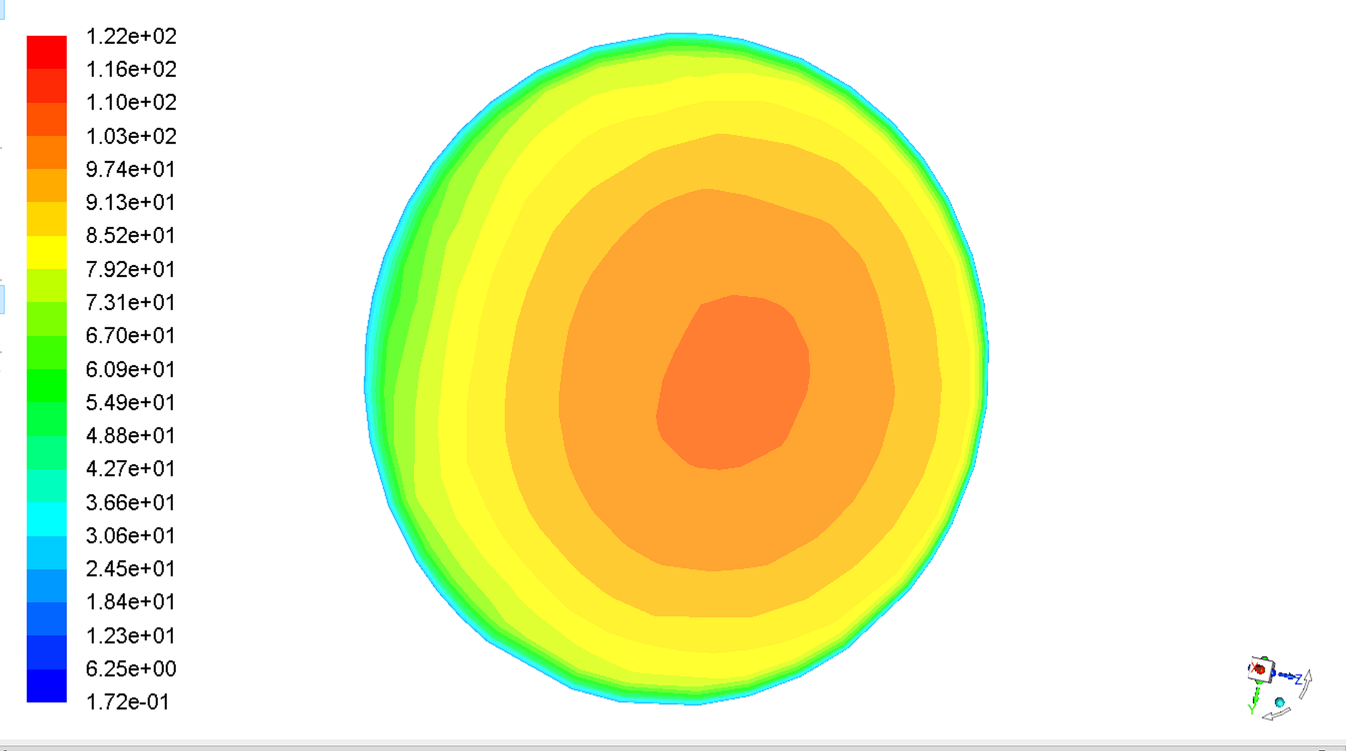  (8) |
| 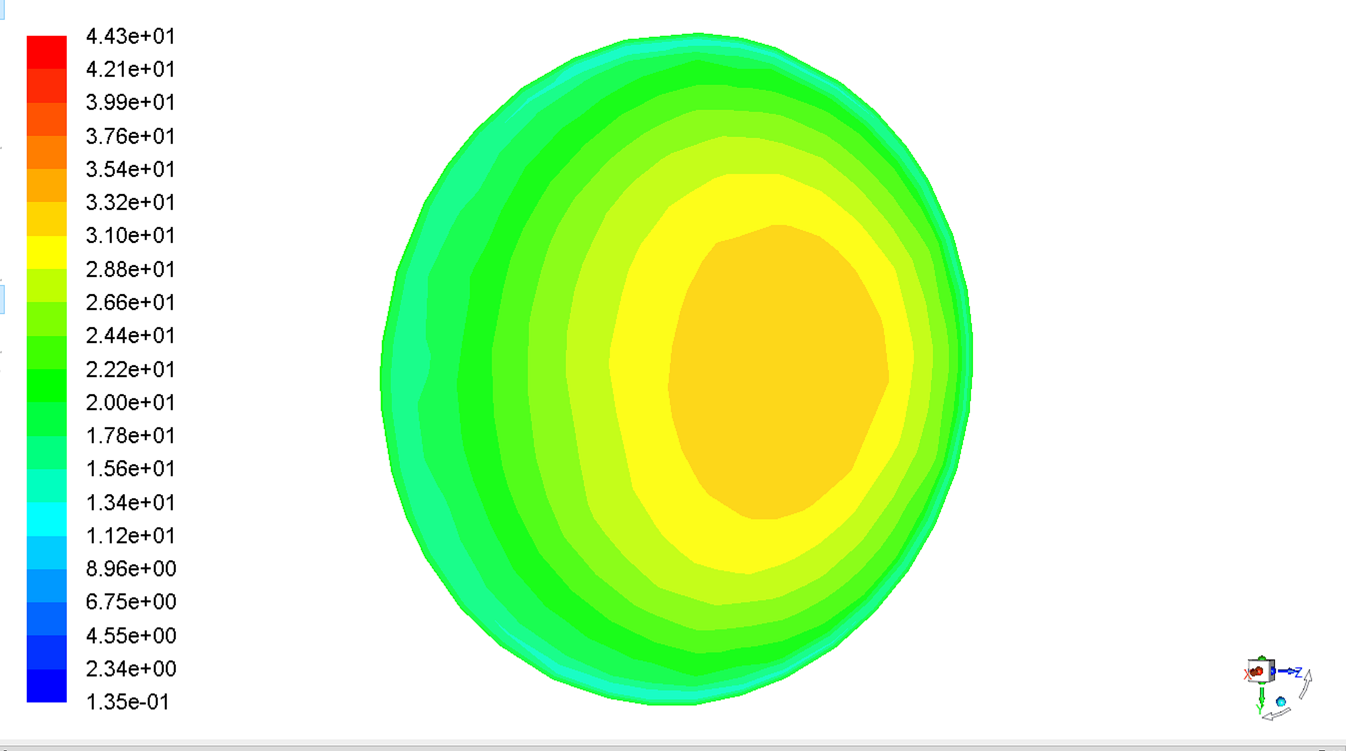  (9) | 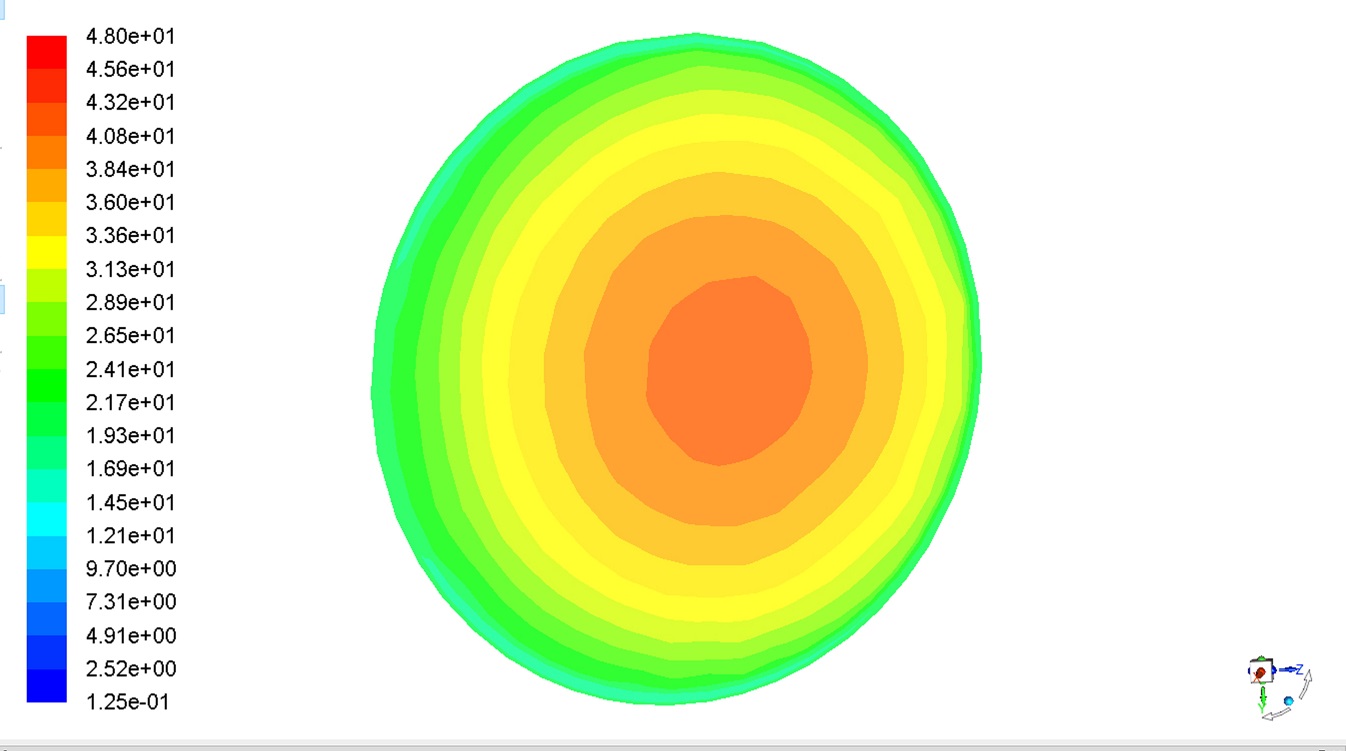  (10) | 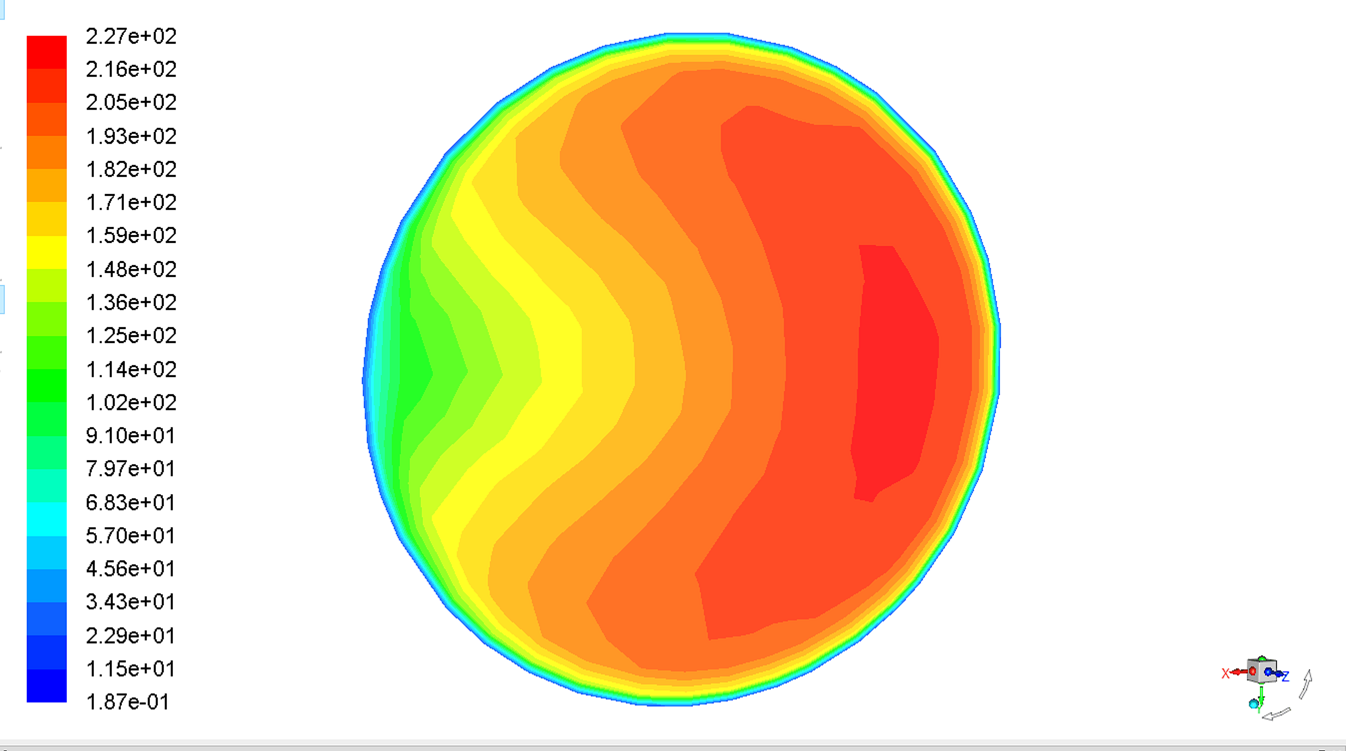  (11) | 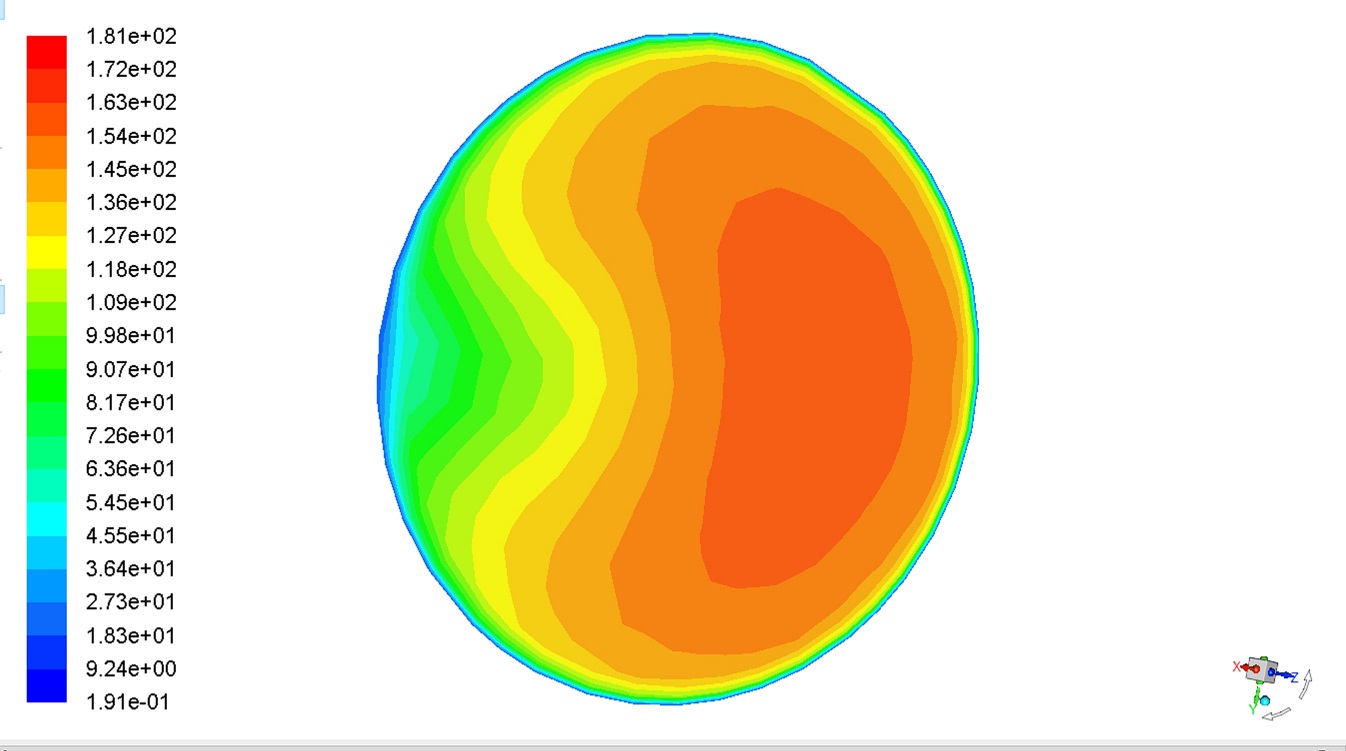  (12) |
| 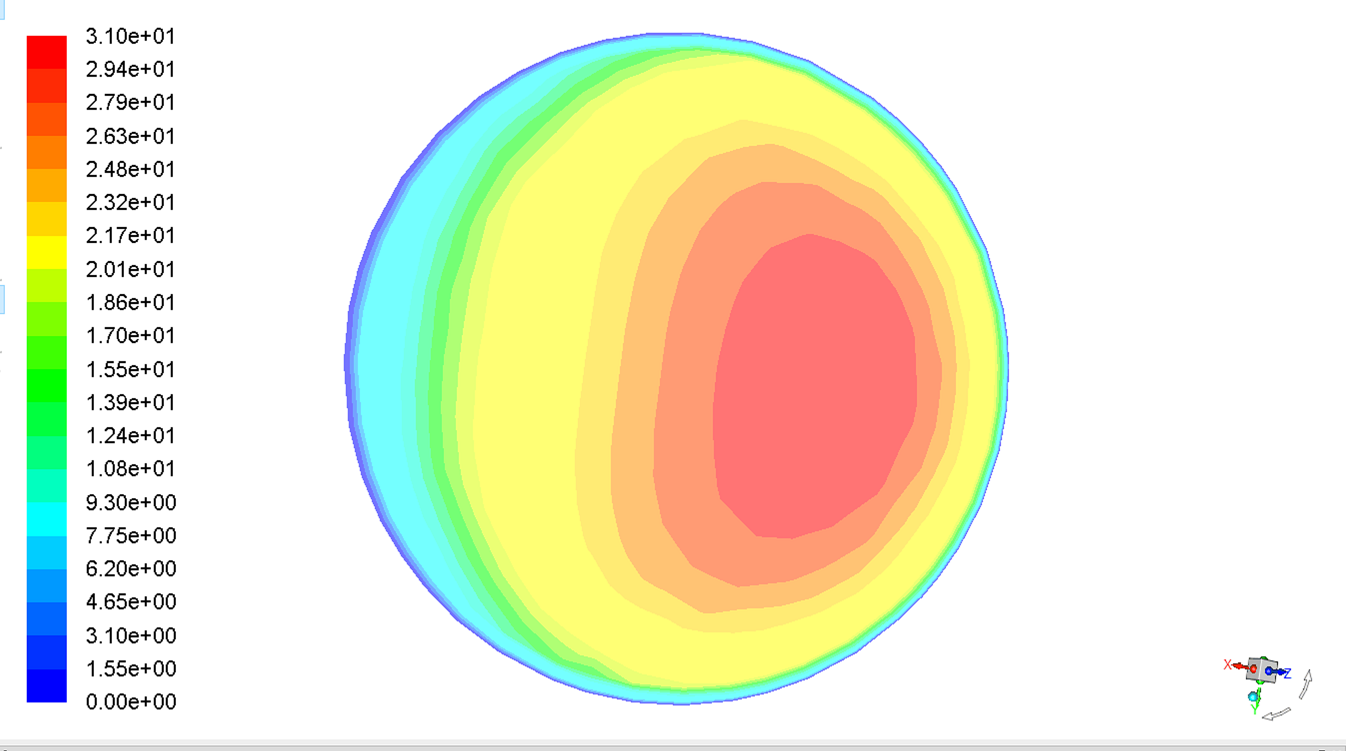  (13) | 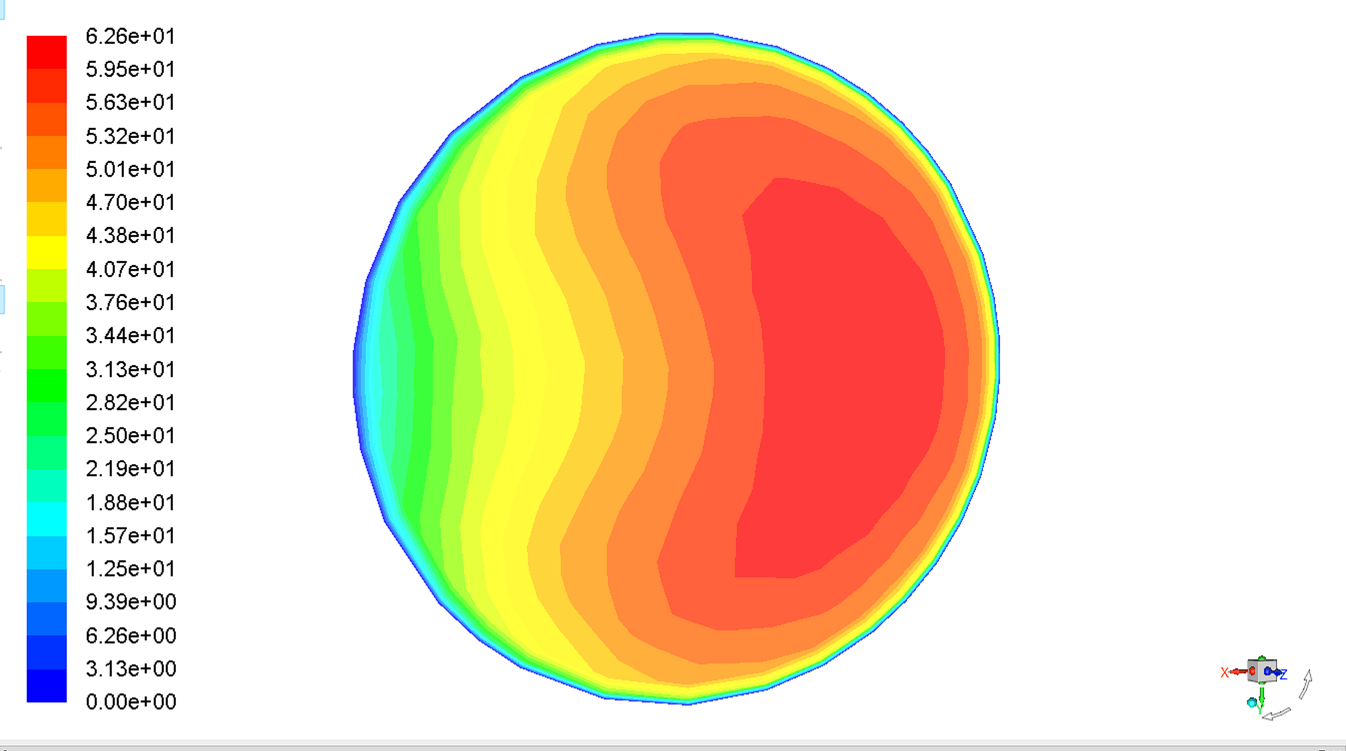  (14) | (15) | (16) |
| **Figure S16 |** Solution velocity distribution at the outlet section of curved-pipe nozzles in 16 tests | | | |

| **Table S1 Range analysis of orthogonal test about stepped nozzles** | | | | |
| --- | --- | --- | --- | --- |
|  | A | B | C | D |
| K_1_ | 41.6674 | 45.0275 | 35.4096 | 83.1865 |
| K_2_ | 57.1826 | 59.5631 | 49.3541 | 40.9823 |
| K_3_ | 53.6907 | 47.9503 | 67.7770 | 28.3719 |
| R | 15.5152 | 14.5356 | 32.3674 | 54.8146 |

| **Table S2 Range analysis of orthogonal test about conical-straight nozzles** | | | | |
| --- | --- | --- | --- | --- |
|  | A | B | C | D |
| K_1_ | 81.9978 | 86.7413 | 61.5587 | 144.2915 |
| K_2_ | 103.4238 | 107.2733 | 91.9358 | 83.4524 |
| K_3_ | 98.5525 | 89.9595 | 130.4796 | 56.2301 |
| R | 21.4260 | 20.5320 | 68.9209 | 88.0614 |

| **Table S3** Range analysis of orthogonal test about conical nozzles | | | |
| --- | --- | --- | --- |
|  | A | B | C |
| K_1_ | 87.8202 | 61.8953 | 145.8432 |
| K_2_ | 107.985 | 92.5386 | 84.7445 |
| K_3_ | 91.0856 | 132.4569 | 56.3031 |
| R | 20.1648 | 70.5616 | 89.5401 |

| **Table S4 Range analysis of orthogonal test about curved-pipe nozzles** | | | | | |
| --- | --- | --- | --- | --- | --- |
|  | A | B | C | D | E |
| K_1_ | 63.703 | 38.334 | 161.627 | 72.908 | 77.096 |
| K_2_ | 72.548 | 64.314 | 85.860 | 88.989 | 98.455 |
| K_3_ | 94.535 | 96.499 | 51.846 | 95.829 | 83.977 |
| K_4_ | 103.586 | 135.226 | 35.040 | 76.648 | 74.845 |
| R | 39.882 | 96.891 | 126.586 | 22.920 | 23.610 |
